# Partial Dominance, Overdominance, Epistasis and QTL by Environment Interactions Contribute to Heterosis in Two Upland Cotton Hybrids

**DOI:** 10.1534/g3.115.025809

**Published:** 2015-12-29

**Authors:** Lianguang Shang, Yumei Wang, Shihu Cai, Xiaocui Wang, Yuhua Li, Abdugheni Abduweli, Jinping Hua

**Affiliations:** * Department of Plant Genetics and Breeding, Key Laboratory of Crop Heterosis and Utilization of Ministry of Education, Beijing Key Laboratory of Crop Genetic Improvement, China Agricultural University, Beijing 100193, China; † Research Institute of Cash Crops, Hubei Academy of Agricultural Sciences, Wuhan 430064, Hubei, China

**Keywords:** QTLs, heterosis, partial dominance, overdominace, epistasis, Upland cotton

## Abstract

Based on two recombinant inbred line (RIL) populations, two corresponding backcross (BC) populations were constructed to elucidate the genetic basis of heterosis in Upland cotton (*Gossypium hirsutum* L.). The yield, and yield components, of these populations were evaluated in three environments. At the single-locus level, 78 and 66 quantitative trait loci (QTL) were detected using composite interval mapping in RIL and BC populations, respectively, and 29 QTL were identified based on mid-parental heterosis (MPH) data of two hybrids. Considering all traits together, a total of 50 (64.9%) QTL with partial dominance effect, and 27 (35.1%) QTL for overdominance effect were identified in two BC populations. At the two-locus level, 120 and 88 QTL with main effects (M-QTL), and 335 and 99 QTL involved in digenic interactions (E-QTL), were detected by inclusive composite interval mapping in RIL and BC populations, respectively. A large number of QTL by environment interactions (QEs) for M-QTL and E-QTL were detected in three environments. For most traits, average E-QTL explained a larger proportion of phenotypic variation than did M-QTL in two RIL populations and two BC populations. It was concluded that partial dominance, overdominance, epistasis, and QEs all contribute to heterosis in Upland cotton, and that partial dominance resulting from single loci and epistasis play a relatively more important role than other genetic effects in heterosis in Upland cotton.

Heterosis, defined as when the F_1_ generation hybrid tends to better performance than its homozygous parents (Shull 1908; East 1908), is widely exploited in crop plants; however, the molecular genetic mechanisms remain enigmatic (Schnable and Springer 2013). The development of molecular quantitative genetics has facilitated the study of the genetic basis of heterosis in crops (Paterson *et al.* 1988; Stuber *et al.* 1992), and three major hypotheses have been suggested to explain the underlying genetic mechanism: the dominance, overdominance and epistasis hypotheses. The dominance theory results from the complementation of slightly deleterious recessive alleles that exist in the inbred parents (Jones 1917). A host of studies suggested that the genetic basis of heterosis might be attributable to dominance (Xiao *et al.* 1995; Cockerham and Zeng 1996; Swanson-Wagner *et al.* 2009). The single locus over-dominance hypothesis attributes heterosis to the superior performance of heterozygosity over homozygous genotypes (Shull 1908; East 1908). Support for overdominance as the primary genetic basis of heterosis is available in different crops (Stuber *et al.* 1992; Li *et al.* 2001; Luo *et al.* 2001; Mei *et al.* 2005; Semel *et al.* 2006; Krieger *et al.* 2010). Epistasis refers to the interaction between alleles from different loci (Richey 1942; Williams 1959). Epistasis was confirmed to explain heterosis using molecular quantitative genetics methods and statistics (Yu *et al.* 1997; Hua *et al.* 2002, 2003; Luo *et al.* 2009; Tang *et al.* 2010; Zhou *et al.* 2012). Studies from various crops have thus generally indicated that the genetic mechanism of heterosis is complex, and there is no single explanation for hybrid vigor.

Several genetic populations have been developed to study heterosis in crops (Schnable and Springer 2013). F_2_ populations are mainly used to study heterosis; however, they do not allow repeated experiments under multiple environmental conditions to be conducted (Yu *et al.* 1997). Other attempts to construct new populations have included the following: (1) a backcross population (BC) developed from a recombinant inbred line (RIL) population (Xiao *et al.* 1995; Mei *et al.* 2005); (2) an “immortalized F_2_” (IF_2_) population derived from pair crosses of RILs (Hua *et al.* 2002, 2003). A lot of heterosis studies have been carried out in different crops, such as rice (Xiao *et al.* 1995; Li *et al.* 2001; Li *et al.* 2008; Luo *et al.* 2009; Jiang *et al.* 2014), maize (Frascaroli *et al.* 2007; Lariepe *et al.* 2012), and rape (Radoev *et al.* 2008; Basunanda *et al.* 2010) using a backcross design to develop experimental populations. However, reports on backcross populations are not available in Upland cotton (*Gossypium hirsutum* L.). Garcia *et al.* (2008) found that the genetic basis of yield heterosis is quite different for maize and rice. This distinction seems to be related to open- or self-pollination of the respective species. It is indispensable to systemically study the genetic mechanism of heterosis in Upland cotton (cross pollination).

Upland cotton is cultivated widely, and contributes most of the natural commercial textile fiber in the world. So far, more than 2274 cotton QTL are available in Cotton QTLdb (Said *et al.* 2015). Sequences of the D_5_ genome (*Gossypium raimondii*), A_2_ genome (*Gossypium arboreum*), and AD_1_ genome (*G. hirsutum*) are available (Paterson *et al.* 2012; Wang *et al.* 2012; F. Li *et al.* 2014, 2015; Zhang *et al.* 2015), and will facilitate development of new markers for high-resolution genetic map construction, further fine mapping, and candidate gene cloning in Upland cotton in the near future.

The mechanisms of the genetic basis of heterosis differ in different crops (Schnable and Springer 2013). Significant heterosis exists in Upland cotton for yield, as well as yield components (Meredith and Bridge 1972; Galanopoulou-Sendouca and Roupakias 1999; Liu *et al.* 2012; Liang *et al.* 2015). Studies in Upland cotton have yielded various results based on different genetic backgrounds and populations. Recently, Liu *et al.* (2012) developed both the RIL and IF_2_ populations to conduct QTL mapping for yield traits. The results revealed that both dominance and overdominance contributed to heterosis of the XZM 2 hybrid, and dominance played a more important role in cotton yield performance. Mapping heterotic QTLs (hQTLs) for yield and agronomic traits using chromosome segment introgression lines of cotton revealed that the overdominant effect mainly contributed to heterosis (Guo *et al.* 2013). Ning *et al.* (2014) developed a population of 180 RILs from a cross between ‘Prema’ and Chinese cultivar ‘86-1’, and identified 13 QTL for seed-cotton yield, nine QTL for lint percentage, and 12 nonredundant QTL for boll weight. More recently, 70 QTL for yield components were identified using 178 RILs derived from a cross between acc ‘DH962’ and cv ‘Jimian 5′, and, among these, four QTL were detected in more than one environment (Wang *et al.* 2015). More attention has been paid to studies on mapping QTL of yield, fiber quality, and other agronomical traits in Upland cotton by developing intraspecific genetic maps (Sun *et al.* 2012; Zhang *et al.* 2012; Liang *et al.* 2013, 2014; Yu *et al.* 2014; Tang *et al.* 2015; Shang *et al.* 2015a). The study of heterosis has hitherto been neglected in Upland cotton. To elucidate the genetic basis of Upland cotton heterosis, we developed two BC populations based on two RILs, and conducted QTL genetic analysis for yield traits and heterosis performance under multiple environmental conditions. The hQTL and the genetic effects of both the homozygous and heterozygous genotypes were explored using composite interval mapping, and inclusive composite interval mapping. This study will provide new insights into our understanding of the genetic mechanism of heterosis in Upland cotton.

## Materials and Methods

### Plant materials and population construction

Two hybrids were employed in the present research. One is ‘Xinza 1’ (*G. hirsutum*) (Liang *et al.* 2013, 2014, 2015; Shang *et al.* 2015a, 2015b, 2016; hereafter referred to as ‘XZ hybrid’), derived from a cross of ‘GX1135’ and ‘GX100-2’. The other has a common female parent with ‘Xinza 1’, derived from a cross between ‘GX1135’ and ‘VGX100-2’ (hereafter referred to as “XZV” hybrid). ‘VGX100-2’ was selected from ‘GX100-2’ and has significantly different agronomy performance compared with ‘GX100-2’.

Totally, four populations were used based on experimental design. (1) The first population was an RIL population; a 177 RIL population of the F_10_ generation was developed by single seed descent from the F_1_ of ‘Xinza 1’ (Shang *et al.* 2015a). (2) The second population was an RILV population from an XZV hybrid; 180 RILs were developed through 10 consecutive selfing generations. (3) The third population was a backcross developed from a RIL population of the XZ hybrid; 177 BCF_1_ hybrids were obtained, each from a cross where one RIL was used as the female parent, and the common parent, GX1135, was used as the male parent. (4) The fourth population was another backcross population (XZV); 180 BCF_1_ hybrids were developed from crosses between RILs from the RILV population used as the female parent, and the shared parent, GX1135, was used as the male parent.

Two commercial hybrids of Upland cotton (*G. hirsutum*) were used as controls (Shang *et al.* 2015a). The F_1_ hybrid ‘Ruiza 816’ was used as control (CK_1_) at two locations, Handan (E1), Hebei Province, and Cangzhou (E2), Hebei Province. It was bred by the Cotton Research Institute of Dezhou (Shandong Province, China) and Biotechnology Research Institute, Chinese Academy of Agricultural Sciences, and released as a cultivar in Shandong Province, as well as in the cotton-growing area in Yellow River Region, China, in 2007. The F_1_ hybrid “Ezamian 10” was used as control (CK_2_) at location Xiangyang (E3), Hubei Province. It was bred by the Huimin Seed Company, Limited (Hubei Province, China), and Biotechnology Research Institute, Chinese Academy of Agricultural Sciences in 2005, and released in Hubei Province as well as in the Yangtze River Region.

Additionally, two special plots, each consisting of two rows of XZ hybrid, ‘Xinza 1’ F_1_, and its parents GX1135 and GX100-2, respectively, were used as controls in the experiment with populations 1 and 3. Similar controls were set for experiments with populations 2 and 4; each plot consisting of the XZV hybrid F_1_, and its parents GX1135 and VGX100-2.

### Field arrangements and trait evaluation

The four populations, control, and control hybrids, were planted at three locations in 2012. As described above, CK_1_ is ‘Ruiza 816’ at E1 and E2; and CK_2_ is ‘Ezamian 10’ at E3. In the experiment with populations 1 and 2, two-row plots with each line were used. But in the experiment with populations 3 and 4, six-row plots with each plot consisting of two rows of BCF_1_ hybrid [RIL(V) × GX1135] in the middle, and two rows at both sides for the corresponding parents: one side with two rows for the corresponding RIL(V)′, and the other two rows for GX1135. The trait values for each BCF_1_ hybrid, RIL(V)′ × GX1135, were calculated based on the corresponding female RIL(V)′, and the common parent, GX1135, in each plot to control for error. Each line in the RIL(V)′ population was used as the female parent in the BC(V) population, and was the same one in the RIL(V) population. For ease of description, we will refer to the RIL(V)s in the BC(V) population as the RIL(V)′ population, respectively. So, in both populations 3 and 4, the females were all marked as RIL(V)′.

The field planting followed a randomized complete block design with two replications at each location. The plant densities were about 51,000 individuals per hectare at E1 and E2, and 37,000 individuals per hectare at E3. Field management followed the local conventional standard field practices. Bolls from eight plants, including four consecutive plants starting from the second individual in the first row, and four plants in the second rows of each block, were sampled at three locations, respectively. Boll samples were ginned for seed-cotton yield per plant (SY, grams per plant), lint yield per plant (LY, grams per plant), bolls/plant (BNP), boll weight (BW, grams), and lint percentage (LP, %).

### DNA isolation, genotype analysis, and linkage map construction

Young leaves were collected from labeled parents, two RILs, and F_1_ individuals, frozen in liquid nitrogen and stored at –80°. Extraction of individual genomic DNA and population genotype analysis were carried out following the methods of Liang *et al.* (2013). A total of 48,836 pairs of SSR primers was used to screen polymorphic loci between three parents. Totally, 653 polymorphic loci for XZ hybrid, and 382 polymorphic loci for XZV hybrid were acquired and used to conduct genotype analysis of the population. MAPMAKER 3.0 was used to construct a genetic linkage map using the *Kosambi* mapping function (Lander *et al.* 1987).

### Data analysis

Midparent heterosis (MPH) of each BC(V)F_1_ was calculated as follows: MPH = F_1_ – MP, where F_1_ was the mean trait value of the BC(V)F_1_, and MP = [RIL(V)′ + GX1135] / 2 were the midparental trait values of the corresponding female RIL(V)′ and the recurrent parent. The genotype for each BC(V)F_1_ was deduced on the basis of the RIL(V)′s genotype used as the parent for the cross. For XZ and XZV hybrids, QTL analysis was carried out separately for the RIL(V) and BC(V)F_1_ populations. For the RIL(V) and RIL(V)′ populations, the mean trait values from two replications were used as raw data at each location. For each of the BC(V)F_1_ populations, the mean trait values and MPH of the BC(V)F_1_s were used independently as raw data (Mei *et al.* 2005) at three locations. Single-locus QTL were conducted using composite interval mapping by WinQTL Cartographer 2.5 in RIL(V)′, RIL(V), BC(V)F_1_, and MPH data (Zeng 1994; Wang *et al.* 2005). A stringent LOD threshold of 3.0 was used to declare suggestive QTL, whereas the same QTL in another environment or population with LOD of at least 2.0 was considered to be a common QTL (Liang *et al.* 2013). The graphic representation of the linkage group and QTL marker were created by Map Chart 2.2 (Voorrips 2002). QTL nomenclature used in rice was employed (McCouch *et al.* 1997). QTL detected simultaneously in different data sets allowed an assessment of the degree of dominance (Radoev *et al.* 2008). At the single-locus level, the genetic effects of BCF_1_s were defined as follows: a = (P1P1 – P2P2) / 2; MPH = d = (BCF_1_ – (P1P1 + P2P2) / 2) and BCF_1_ = (a + d) (P1 is the recurrent parent). QTL detected only in RIL or BCF_1_ and not for MPH were considered as additive. QTL with d/a ≤ 1 were referred to as being complete or partial dominant loci. QTL with d/a > 1, or only detectable for MPH data, were referred to as over-dominant loci. Two-locus analysis that tests the main-effect QTL (M-QTL), and digenic epistatic QTL (E-QTL), and their environmental interactions (QTL × environment, QE), was conducted using RIL(V), BC(V)F_1_, and MPH data by the software ICIMapping 4.0 (www.isbreeding.net). A threshold of LOD ≥ 2.5 was used for declaring the presence of M-QTL and LOD ≥5.0 E-QTL was used for declaring the presence of E-QTL. Basic statistical analysis was implemented by the software SPSS, version 19.0 (SPSS, Chicago).

### Data availability

All of our raw data are available as Supporting Information Table S9 and Table S10, which include genotypes and traits of two hybrids.

## Results

### Performance of yield and yield components

Measurements of yield and yield components were made for RIL(V), BC(V)F_1_ and MPH data in three environments in XZ and XZV hybrids (Table 1). In XZ and XZV hybrids, the parent GX1135 had a significantly higher phenotypic value than GX100-2 and VGX100-2 for seed cotton yield (SY), lint yield (LY), bolls per plant (BNP), and lint percent (LP), respectively. Significant heterosis for SY and LY was observed in two hybrids. A wide range of variation was observed for yield and yield component traits in the RIL(V), BC(V)F_1_, and MPH data. The SY and LY traits showed a high level of heterosis; however, other yield component traits were relatively low in the three environments. Obviously, the SY and LY traits at E3 showed the highest level of heterosis compared with E1 and E2. This conforms to the fact that hybrid cotton is planted widely in the Yangtze River Valley (E3) in China. The means of the BC(V)F_1_ population were higher than those of the RIL(V)s for most traits in the three environments. The means of the MPH data for SY and LY were high. Nevertheless, the mean values of the MPH data for other traits were relatively low, whereas the means of the MPH data and low MPH (%) of F_1_ were observed at E1, probably due to high rainfall, which gave rise to boll rot during experiments; the performance of CK at E1 was also in agreement with this. The order of the mean values of yield and yield components in MPH data was SY > LY > BNP > BW > LP. Most of the trait values of extreme lines in the RIL(V) and BC(V)F_1_ populations exceeded those of hybrids and CK at three locations, Meanwhile, many individuals showed higher MPH in the BCF_1_ population than in the two hybrids (Table 1).

**Table 1:** Summary statistics on yield and yield components of RIL, BC, and MPH data in two hybrids

Trait	Env.	Mean			Min			Max			Parents			MPH	CK_1_
		RIL	BC	MPH	RIL	BC	MPH	RIL	BC	MPH	♀	♂	F_1_	%	CK_2_
XZ hybrid															
SY (g)	E1	57.27	70.14	6.30	28.23	47.37	−12.33	93.74	90.37	27.44	71.23	69.99	72.35	2.46	88.36
	E2	65.92	79.69	9.13	33.12	53.56	−18.02	90.46	101.17	30.87	75.75	68.85	97.21	34.45	97.62
	E3	81.64	95.06	12.93	38.22	57.25	−11.78	125.31	144.69	42.59	80.81	73.44	116.59	51.17	98.09
LY (g)	E1	22.39	27.55	2.35	10.72	17.65	−4.99	37.71	37.78	12.65	28.29	23.74	27.98	7.55	35.68
	E2	25.63	30.83	3.56	11.40	21.52	−7.00	36.36	40.10	12.35	30.18	22.43	38.12	44.92	40.55
	E3	31.07	36.79	5.16	14.45	21.14	−5.45	52.24	55.21	16.16	31.33	25.45	44.44	56.53	40.96
BNP	E1	16.50	18.96	0.80	7.25	12.60	−3.81	28.06	25.00	8.03	19.34	16.56	19.97	11.25	16.28
	E2	20.63	23.90	1.25	11.63	19.00	−4.94	27.06	29.31	6.16	24.63	21.59	27.63	19.56	20.34
	E3	21.16	21.25	0.61	14.06	15.38	−7.03	30.75	29.50	7.14	20.38	17.66	21.81	14.67	21.69
BW (g)	E1	4.84	4.90	0.30	3.46	4.24	−0.33	6.39	5.55	0.99	4.71	5.23	5.19	4.43	6.37
	E2	4.22	4.54	0.27	3.02	3.70	−0.42	5.14	5.21	0.93	4.50	4.86	4.97	6.20	6.41
	E3	4.77	5.12	0.33	3.48	4.32	−0.81	5.80	5.82	1.31	5.03	5.12	5.35	5.42	5.73
LP (%)	E1	39.16	39.27	−0.18	33.40	35.60	−3.02	45.45	44.06	3.50	39.68	33.89	38.67	5.12	40.39
	E2	38.93	38.70	0.08	34.39	35.11	−2.33	43.42	42.47	3.37	39.78	32.50	39.17	8.38	41.49
	E3	37.95	38.69	0.23	31.42	34.10	−2.89	43.77	43.05	4.38	38.80	34.70	38.09	3.65	41.72
XZV hybrid															
SY (g)	E1	58.00	55.56	−4.62	30.24	25.50	−31.49	96.26	86.77	26.82	63.76	55.38	69.95	17.42	82.64
	E2	63.79	84.95	13.49	20.08	60.18	−11.55	103.82	107.16	37.31	88.28	64.64	91.72	19.96	108.32
	E3	67.09	105.49	27.29	13.69	64.94	−14.10	132.75	145.06	79.72	66.13	51.52	102.88	74.89	106.69
LY (g)	E1	22.15	20.56	−1.37	10.85	9.44	−11.17	36.97	33.91	11.27	23.78	20.22	27.42	24.64	33.45
	E2	24.44	31.91	5.16	7.66	21.07	−4.28	44.20	40.69	13.37	33.90	24.29	34.93	20.05	46.02
	E3	26.96	43.42	11.84	6.05	26.51	−5.97	57.54	59.68	33.57	25.59	19.73	42.83	89.01	45.91
BNP	E1	15.08	17.68	2.19	8.50	12.13	−2.53	21.56	23.69	7.97	16.15	15.28	19.63	24.91	15.75
	E2	20.32	24.71	3.49	7.18	18.69	−3.38	32.63	31.69	12.47	26.47	18.34	26.08	16.40	21.34
	E3	15.81	20.97	4.27	4.89	15.13	−5.31	29.13	29.56	13.61	15.09	13.15	20.88	47.88	20.66
BW (g)	E1	4.41	4.71	0.22	2.94	3.90	−0.55	5.57	5.49	1.11	4.46	4.71	5.11	11.45	6.57
	E2	4.21	4.67	0.21	3.29	4.18	−0.46	5.47	5.59	1.27	4.25	4.48	4.62	5.84	6.56
	E3	5.00	5.14	0.21	3.72	4.00	−0.88	6.43	6.10	1.54	4.92	4.86	5.42	10.84	5.69
LP (%)	E1	38.10	36.97	0.50	28.08	32.38	−2.17	43.49	43.99	7.04	37.48	36.66	39.18	5.69	40.46
	E2	38.32	37.54	0.07	27.48	33.28	−3.43	43.92	42.01	2.88	38.49	37.59	38.04	0.00	42.44
	E3	39.97	41.13	0.84	31.40	38.39	−2.31	46.96	44.91	4.15	38.99	38.40	41.56	7.40	43.06

MPH was calculated as follows: MPH = F_1_ – MP, where F_1_ was the mean trait value of the BC(V)F_1_, and MP = [RIL(V)’ + GX1135]/2 was the mid-parental trait values of the corresponding female RIL(V)’ and the recurrent parent. MPH (%) = (F_1_ – MP) / MP%, where F_1_ was the mean trait value of, XZ or XZV hybrid and MP = (GX1135 + GX100 – 2(V)) / 2 was the mid-parental trait values of parents of XZ or XZV hybrid. CK_1_ is ‘Ruiza 816’ at Handan (E1) and Cangzhou (E2); CK_2_ is ‘Ezamian 10’ at Xiangyang (E3). Env., Environment; MPH, mid-parental heterosis; RIL, recombinant inbred line population; BC, backcross population; SY, seed cotton yield; E1, Handan; E2, Cangzhou; E3, Xiangyang; LY, lint yield; BNP, bolls per plant; BW, boll weight; LP, lint percent.

### Relationships between the means of RILs, MPH, and BCF_1_ performance

For XZ hybrid and XZV hybrids, the correlation coefficients among the mean values of BC(V)F_1_s, their MPH, and the means of RILs for the yield and yield components are shown in Table 2. Significant high positive correlations between MPH and BC(V)F_1_s performance were observed for all traits in the three environments. Most of the trait values of the RIL(V)s, and that of their BC(V)F_1_s, showed significant positive correlation. Population performance of the BC(V)F_1_ for most of traits was determined largely by performance of the parental RIL(V)s. There were negative correlations between most trait values of RIL(V)s and MPH in all three environments.

**Table 2:** Correlations between RIL, BC, and MPH data in two backcross populations

Trait	Env.	Between RILs and BC		Between RILs and MPH		Between BC and MPH	
		XZ Hybrid	XZV Hybrid	XZ Hybrid	XZV Hybrid	XZ Hybrid	XZV Hybrid
SY	E1	0.24**	0.29**	−0.09	0.11	0.67**	0.77**
	E2	0.23**	0.26**	−0.09	−0.37**	0.72**	0.57**
	E3	0.38**	0.21**	−0.16*	−0.67**	0.65**	0.41**
LY	E1	0.19*	0.32**	−0.05	0.11	0.65**	0.76**
	E2	0.25**	0.30**	−0.03	−0.32**	0.76**	0.57**
	E3	0.42**	0.20**	−0.13	−0.69**	0.67**	0.42**
BNP	E1	0.16*	0.36**	−0.15	−0.22**	0.72**	0.52**
	E2	0.07	0.22**	−0.24**	−0.44**	0.63**	0.57**
	E3	0.30**	0.25**	−0.09	−0.74**	0.73**	0.32**
BW	E1	0.42**	0.54**	−0.13	−0.04	0.62**	0.54**
	E2	0.53**	0.40**	−0.23**	−0.16*	0.51**	0.59**
	E3	0.54**	0.38**	−0.05	−0.05	0.56**	0.62**
LP	E1	0.44**	0.44**	0.09	−0.21**	0.42**	0.53**
	E2	0.66**	0.46**	−0.03	−0.02	0.58**	0.46**
	E3	0.66**	0.46**	−0.22**	−0.19*	0.40**	0.40**

Env., Environment; RIL, recombinant inbred line population; BC, backcross population; MPH, mid-parental heterosis; SY, seed cotton yield; E1, Handan; E2, Cangzhou; E3, Xiangyang; LY, lint yield; BNP, bolls per plant; BW, boll weight; LP, lint percent.

* *P* < 0.05; ** *P* < 0.01.

### Relationship between whole-genome heterozygosity and performance

For most traits, a poor relationship was observed between heterozygosity of whole-genome and the performance of BC(V)F_1_ and MPH data in terms of yield and yield components in two hybrids (Table 3). The result suggested that overall genome heterozygosity alone had little effect on trait performance, and that heterosis might be derived from just a small amount of genome heterozygosity in the BC(V)F_1_ data. The low correlation coefficients may be the result of low density loci for the whole genome, and only half the heterozygosity of the whole genome existed in BC. These results verified previous reports (Hua *et al.* 2002; Mei *et al.* 2005; Luo *et al.* 2009).

**Table 3:** Correlation coefficients between genotypic heterozygosity and trait performance in BC and MPH data in two hybrids

Trait	BC						MPH					
	E1		E2		E3		E1		E2		E3	
	XZ	XZV	XZ	XZV	XZ	XZV	XZ	XZV	XZ	XZV	XZ	XZV
SY	0.05	0.02	0.04	−0.06	0.08	0.05	−0.01	−0.04	0.02	−0.07	0.12	0.10
LY	0.05	0.04	0.07	−0.04	0.09	0.09	−0.02	−0.05	0.04	−0.07	0.12	0.12
BNP	0.00	0.11	0.11	0.00	0.02	−0.03	0.00	0.19*	0.04	−0.00	−0.03	0.07
BW	0.04	0.13	0.10	0.12	0.00	0.06	0.14	0.14	0.17*	0.08	−0.05	−0.07
LP	0.01	0.07	0.12	0.05	0.04	0.25**	−0.07	−0.06	0.05	−0.01	−0.02	0.00

BC, backcross population; MPH, mid-parental heterosis; E1, Handan; E2, Cangzhou; E3, Xiangyang; XZ, XZ hybrid; XZV, XZV hybrid; SY, seed cotton yield; LY, lint yield; BNP, bolls per plant; BW, boll weight; LP, lint percent.

* *P* < 0.05; ** *P* < 0.01.

### Maps and single-locus QTL controlling yield and yield components

Genetic maps for the two hybrid populations were constructed based on the polymorphic loci identified (Supporting Information, Figure S1). For XZ hybrid, 623 loci were mapped to 32 linkage groups, and the genetic map spanned 3889.9 cM with an average distance of about 6.2 cM between adjacent markers. For XZV hybrid, 308 loci were mapped to 39 linkage groups, and the genetic map spanned 3048.4 cM with an average distance about 9.9 cM between adjacent markers. QTL detected using composite interval mapping for yield and yield components in XZ and XZV hybrids are shown in Table S1. Table 4 lists numbers for different effects of QTL identified by composite interval mapping in three environments in the backcross population. A total of 91 and 54 QTL were detected for yield and yield components in five data sets of XZ and XZV hybrids, respectively. For seed cotton yield (SY), in the XZ hybrid, a total of 20 QTL were detected in four data sets, among which 11, 10, nine and three QTL were identified in the RIL′s, RILs, BCF_1_ hybrids, and MPH data, respectively. Twelve QTL were identified in more than two environments or populations. In the backcross population, three QTL for an additive effect, three for a partial dominant (PD) effect, and six for an overdominant (OD) effect, were observed (Table 4). In the XZV hybrid, a total of 13 QTL were detected, among which eight, eight, three and five QTL were identified in the RILV′s, RILVs, BCVF_1_ hybrids, and MPH data, respectively. Eight QTL were identified in more than two environments or populations. In the backcross population, one QTL with an additive effect, five with a PD effect, and two with an OD effect were found (Table 4).

**Table 4:** Effects of QTL identified for yield traits by composite interval mapping in three environments

Trait	XZ Hybrid			XZV Hybrid		
	A	PD	OD	A	PD	OD
SY	3	3	6	1	5	2
LY	3	5	6	3	2	0
BNP	3	2	3	1	4	1
BW	2	5	3	0	4	0
LP	3	12	3	4	8	3
Total	14	27	21	9	23	6

A, additive effect; PD, partial dominant effect; OD, overdominant effect; SY, seed cotton yield; LY, lint yield; BNP, bolls per plant; BW, boll weight; LP, lint percent.

For lint yield (LY), in the XZ hybrid, a total of 19 QTL were detected in four data sets, among which 11, 10, 10 and five QTL were identified in the RIL′s, RILs, BCF_1_ hybrids, and MPH data, respectively. Thirteen QTL were identified in more than two environments or populations. In the backcross population, three QTL for an additive effect, five for a PD effect, and six for an OD effect were observed. In the XZV hybrid, a total of ten QTL were detected, among which six, six, four and one QTL were identified in the RILV′s, RILVs, BCVF_1_ hybrids, and MPH data, respectively. Six QTL were identified in more than two environments or populations. In the backcross population, three QTL for an additive effect, two for a PD effect, and none having an OD effect were found.

For bolls per plant (BNP), in the XZ hybrid, a total of 11 QTL were detected. Seven QTL were identified in more than two environments or populations. Three QTL for an additive effect, two for a PD effect, and three for an OD effect were observed. In the XZV hybrid, seven QTL were detected, and five QTL were identified in more than two environments or populations. In the backcross population, one QTL with an additive effect, four with a PD effect, and one with an OD effect were identified.

For boll weight (BW), in the XZ hybrid, a total of 22 QTL were detected, and 15 QTL were detected in more than two environments or populations. Two QTL for an additive effect, five for a PD effect, and three for an OD effect were observed. In the XZV hybrid, nine QTL were detected, and five QTL were identified in more than two environments or populations. In the backcross population, four QTL for an additive effect were identified. No QTL were detected for PD or OD effect.

For lint percent (LP), in the XZ hybrid, a total of 19 QTL were detected. Eleven QTL were identified in more than two environments or populations. Three QTL for an additive effect, 12 for a PD effect, and three for an OD effect were observed. In the XZV hybrid, 15 QTL were detected, and 11 QTL were identified in more than two environments or populations. In the backcross population, four QTL for an additive effect, eight for a PD effect, and three for an OD effect were identified.

### QTL and QE interactions resolved by two-locus analyses in the RIL(V)s and BC(V) populations

A total of 147 and 101 M-QTL and QEs were detected by inclusive composite interval mapping (ICIM) in five traits of XZ and XZV hybrids, respectively (Table S2, Table S3, and Table S4). In the XZ hybrid, a total of 75, 52 and 20 M-QTL and QEs were detected in the RILs, BCF_1_ hybrids, and MPH data, respectively. On average, M-QTL explained 2.28%, 2.09%, and 1.10% of the phenotype variation, and the QE explained 0.63%, 0.74%, and 1.30% of the phenotype variation in the RILs, BCF_1_ hybrids, and MPH data, respectively. In the XZV hybrid, a total of 45, 36 and 20 M-QTL and QEs were detected in the RILVs, BCVF_1_ hybrids, and MPH data, respectively. On average, M-QTL explained 2.01%, 1.21%, and 1.38% of the phenotype variation, and the QE explained 0.70%, 1.26%, and 1.19% of the phenotype variation in the RILVs, BCVF_1_ hybrids, and MPH data, respectively.

Totally, 216 and 287 E-QTL and QEs were detected by ICIM in five data sets of XZ and XZV hybrids, respectively (Table S5, Table S6, and Table S7). In the XZ hybrid, a total of 121, 78, and 17 E-QTL and QEs were detected in the RILs, BCF_1_ hybrids, and MPH data, respectively. On average, E-QTL explained 3.30%, 2.88%, and 2.63% of the phenotype variation, and the QE explained 0.33%, 0.91%, and 1.14% of the phenotype variation in the RILs, BCF_1_ hybrids, and MPH data, respectively. In the XZV hybrid, a total of 214, 21, and 52 E-QTL and QEs were detected in the RILVs, BCVF_1_ hybrids, and MPH data, respectively. On average, E-QTL explained 3.09%, 1.87%, and 1.94% of the phenotype variation, and the QE explained 0.77%, 1.79%, and 2.10% of the phenotype variation in the RILVs, BCVF_1_ hybrids, and MPH data, respectively.

## Discussion

### The importance of partial dominance and overdominance conditioning heterosis

At the single-locus level, the QTL detected simultaneously in RIL(V)s, BC(V)F_1_ and MPH data allowed an assessment of the degree of dominance in the XZ and XZV hybrids (Radoev *et al.* 2008). In the XZ hybrid, considering all traits together, a total of 27 (56.3%) QTL for a PD effect, and 21 (43.8%) QTL for an OD effect, were identified in the backcross population (Table 4). In the XZV hybrid, a total of 23 (79.3%) QTL for a PD effect, and six (20.7%) QTL for an OD effect, were identified in the backcross population. The importance of dominance and overdominance controlling the heterosis of XZ and XZV hybrids seemed different. These results revealed that all levels of dominance play a role in controlling the expression of hybrid vigor in Upland cotton. In addition, for almost all traits, partial dominance played a relatively more important role than overdominance. In maize, the phenomenon that QTL for traits with low heterosis existed mainly in the additive to dominance range, and QTL for traits with high heterosis had effects in the dominance to overdominance range, was observed (Frascaroli *et al.* 2007). In a study of heterosis in rapeseed, grain yield with the highest level of heterosis tends to possess the largest number of loci exhibiting overdominance in all traits measured (Radoev *et al.* 2008). This result was in harmony with our present study of QTL detected in the XZ hybrid, while it was not obvious in the XZV hybrid.

### Feature of heterotic QTL

Heterotic QTL (hQTLs) can be identified directly using MPH data (Hua *et al.* 2002). In the present research, we detected a total of 17 and 12 hQTLs for yield and yield components, respectively, using MPH data in XZ and XZV hybrids by composite interval mapping (Figure S1, Table S9 and Table S10). In these hQTLs, four obvious features of heterotic QTL were observed. The first feature implies that the hQTLs are sensitive to the environmental conditions, because several hQTLs showed opposite genetic effects in different environments. Genotype by environment interaction was important for the stability of hQTLs, and should be taken into account in plant breeding programs (Xing *et al.* 2002). The second feature was that relatively fewer QTL were identified with the MPH data than with RIL(V)s and BC(V)F_1_ data. The reason for this is probably that these QTL have an intermediate mode of inheritance, and such QTL lacking dominance could not be detected in MPH data. Moreover, QTL with additive effects larger than dominance effects were less likely to be identified in MPH data than in RILs and BCF_1_ data (Radoev *et al.* 2008). The third feature is that the effect of hQTLs is pleiotropic. The phenomenon that various quantitative traits were controlled by the same QTL was universally observed (Liang *et al.* 2013; Shang *et al.* 2015a); however, reports of pleiotropic hQTLs are few. In present study, the QTL *qSY-Chr1-3* and *qLY-Chr1-4*, which were identified using MPH data at E2, were mapped in the same marker interval, suggesting that these heterosis traits were controlled by the same hQTLs, and that the effect of these hQTLs was pleiotropic. These pleiotropic hQTLs also showed the same overdominance effect direction. A similar phenomenon was also observed in the QTL *qSY-Chr1-5* and *qLY-Chr1-6* (Table S1). Also, Frascaroli *et al.* (2007) found that some chromosome regions presented overlaps of overdominant QTL for different traits, suggesting pleiotropic effects on overall plant vigor. The final feature was that hQTLs in the BCF_1_ population were not independent. In the XZ hybrid, six (35.3%) hQTLs were detected independently in MPH data, while 11 out of 17 (64.7%) hQTLs detected in the MPH data set overlapped with QTL in the RIL and BCF_1_ populations (Table 4). In the XZV hybrid, five (41.7%) hQTLs were detected independently in MPH data, while seven out of 12 (58.3%) hQTLs detected in the MPH data set overlapped with QTL in the RILV and BCVF_1_ populations. Previous studies have suggested that hQTLs of yield-related traits were independent, and were different between QTL and hQTLs of yield traits (Hua *et al.* 2003; Tang *et al.* 2010; Guo *et al.* 2013). A recent study showed that dozens of QTL detected for both grain yield and ear length in the MPH data set overlapped with QTL in the IF_2_ population, and that hQTLs were not independent (Guo *et al.* 2014). In the present study, we found that hQTLs were not independent in the BCF_1_ population, which suggested that phenotypes and heterotic traits in Upland cotton might be jointly controlled by several shared loci.

### Whole-genome heterozygosity and trait performance

In the present study, we found that not all the traits possessed higher phenotypes in heterozygotes (BCF_1_s) than in their respective homozygote (RILs). QTL with different levels of negative overdominance were identified for some traits, demonstrating that heterozygosity was not always necessarily advantageous for the expression of the trait and heterosis. It was obvious that the BCF_1_ population, which possessed only half of the possible whole-genome heterozygosity, had fewer heterozygous loci than the hybrid ‘Xinza 1.’ Most of the extreme lines in the RIL(V) and BC(V)F_1_ populations exceeded those of hybrid and CK under different environmental conditions, and dozens of individuals showed higher MPH in the BC(V)F_1_ population than that of the hybrid (Table 1). The level of heterozygous advantage had only a poor correlation between the whole-genome marker heterozygosity and performance in the BC(V)F_1_ population (Table 3). In addition, we observed that double heterozygote genotypes did not show the best phenotypic trait value (data not shown). These results indicated that high hybrid vigor arose mainly from certain loci heterozygosity rather than whole-genome heterozygosity. Similar results were found in rice (Hua *et al.* 2002; Mei *et al.* 2005; Luo *et al.* 2009; Jiang *et al.* 2014). It is noted that these results might be not accurate due to using only a few markers in present genetic maps, compared with using whole-genome markers. However, large-scale sequencing showed recently that overall heterozygosity made little contribution to heterosis in rice, and the accumulation of numerous rare superior alleles with positive dominance is an important contributor to the expression of heterosis in rice hybrids (Huang *et al.* 2015).

### QTL across multiple populations and environments

In our previous study, we identified 13 QTL, including two for SY, two for LY, three for BNP, three for BW, and three for LP in F_2: 3_ and F_2: 4_ populations derived from cross GX1135 (P1) × GX100-2 (P2) (*G. hirsutum* L.) (Table S8; Liang *et al*. 2015). The present RIL population of the F_9_ generation, consisting of 177 RILs along with corresponding backcross populations, was developed from the F_2:3_ population by single seed descent and used in the present research. These 13 QTL for yield traits identified previously were once again detected simultaneously using RIL and backcross population (Table S8). As a good illustration of this, the two closest markers of *qLP-Chr5-1*, GH260 and NAU6240, identified in F_2:3_ and F_2:4_ populations under E1 and E2 environments overlapped the interval of the two closest markers of *qLP-Chr5-2*, SWU20917 and NAU6240, in the present study. In other words, three new primers, PGML0120, SWU20914, and SWU20917, were added to the interval of GH260 and NAU6240 for the QTL *qLP-chr5-1*, and the genetic distance of intervals was reduced in the RIL and backcross populations, which made it more precise than ever before. Unfortunately, we did not detect many identical M-QTL and E-QTL between the XZ and XZV hybrids. One possible reason was that the density of the genetic map of XZV was low compared with the XZ hybrid. The QTL *qLP-Chr4-1* for lint percentage located on chromosome 4, which contained the same marker SWU16783, was detected in the backcross population of both XZ and XZV hybrids. Simultaneously, the marker interval of the QTL *qLP-Chr4-1* was next to the QTL *qLP-Chr4-1*, which was identified in F_2: 3_ and F_2: 4_ populations. In addition, it is worth noting that the QTL *qLP-Chr5-2*, which was detected across six populations and three environments, showed an exceedingly high LOD value with a mean of 7.39, and phenotypic variation with an average of 18.72%. These stable QTL across multiple populations and environments will be important for motivating further interest in implementation of MAS or fine mapping of yield traits. Although several QTL for cotton yield traits were detected previously using an intra-specific map, few shared markers have been used in previous studies and present research (Yu *et al.* 2014). It is difficult to search for the same QTL, because maps, population types, population structure, and environments differ, affecting the comparison of common QTL (Shang *et al.* 2015a).

### Genetic basis of heterosis in Upland cotton

The relative importance of partial dominance and overdominance in XZ and XZV hybrids could be assessed by comparing the genetic effects identified in the RIL(V)s, BC(V)F_1_, and MPH data (Table 5). Totally, at two-locus levels, the number and PV of M-QTL, and E-QTL for most traits identified in RIL(V)s and BC(V)F_1_ data were, on average, larger than those of the M-QTL and E-QTL identified in MPH data, indicating that partial dominance was more important than overdominance. Recently, a large-scale sequencing and phenotyping experiment in hybrid rice varieties revealed that incomplete dominance was a more important contributor to heterosis than overdominance (Huang *et al.* 2015; Birchler 2015). However, overdominance as the primary genetic basis in heterosis has been observed in rice (Li *et al.* 2001; Luo *et al.* 2001) and tomato (Krieger *et al.* 2010). A study mapping hQTLs for yield and agronomic traits using chromosome segment introgression lines showed that the overdominant effect made the main contribution to heterosis in cotton (Guo *et al.* 2013). These results show that the genetic basis of yield heterosis is complex. In MPH data, a greater percentage in QEs of M-QTL and E-QTL in the XZV hybrid than the XZ hybrid was also observed, showing that the heterosis performance of the backcross population was more susceptible in the XZV hybrid.

**Table 5:** Summary of M-QTL and E-QTL detected controlling yield traits by inclusive composite interval mapping in RIL(V), BC(V)F_1_, and MPH data

Trait	RIL			BCF_1_			MPH			RILV			BCVF_1_			MPHV		
M-QTL	*n**^a^*	P(A)*^b^*	P(AE)	*n*	P(A)	P(AE)	*n*	P(A)	P(AE)	*n*	P(A)	P(AE)	*n*	P(A)	P(AE)	*n*	P(A)	P(AE)
SY	13	2.31	0.84	5	1.99	1.33	3	1.05	1.22	6	1.27	1.10	5	0.72	1.54	5	0.57	1.81
LY	15	2.03	0.94	7	1.64	1.05	3	0.81	1.95	7	1.52	0.88	8	0.90	1.59	3	1.28	2.10
BNP	13	2.22	0.49	8	2.11	0.56	4	1.69	0.63	7	2.17	0.67	3	0.47	1.84	6	2.51	0.58
BW	16	2.36	0.43	16	2.26	0.39	5	0.82	1.57	13	2.56	0.41	12	1.94	0.67	5	1.18	1.03
LP	18	2.48	0.47	16	2.45	0.38	5	1.12	1.13	12	2.54	0.47	8	2.02	0.64	1	1.37	0.45
Mean	15.0	2.28	0.63	10.4	2.09	0.74	4.0	1.10	1.30	9.0	2.01	0.70	7.2	1.21	1.26	4.0	1.38	1.19
E-QTL	*n*	P(AA)	P(AAE)	*n*	P(AA)	P(AAE)	*n*	P(AA)	P(AAE)	*n*	P(AA)	P(AAE)	*n*	P(AA)	P(AAE)	*n*	P(AA)	P(AAE)
SY	12	3.06	0.30	8	2.16	1.82	4	2.45	1.90	59	2.17	1.40	2	1.22	3.10	14	1.12	2.89
LY	10	3.05	0.36	10	2.77	1.16	4	2.43	1.90	55	2.32	1.25	7	1.64	2.11	9	0.97	2.89
BNP	15	3.18	0.64	7	3.12	0.96	3	3.33	0.73	67	3.53	0.78	2	0.60	2.39	24	2.51	0.92
BW	36	3.54	0.24	26	3.21	0.41	2	1.49	0.43	11	3.68	0.20	6	3.16	0.57	4	3.11	1.58
LP	48	3.67	0.12	27	3.16	0.21	4	3.48	0.75	22	3.72	0.24	4	2.75	0.81	1	2.01	2.24
Mean	24.2	3.30	0.33	15.6	2.88	0.91	3.4	2.63	1.14	42.8	3.09	0.77	4.2	1.87	1.79	10.4	1.94	2.10

M-QTL, quantitative trait loci with main effects; E-QTL, quantitative trait loci involved in digenic interactions; RIL, recombinant inbred line population; BC, backcross population; MPH, mid-parental heterosis; SY, seed cotton yield; LY, lint yield; BNP, bolls per plant; BW, boll weight; LP, lint percent. Mean, equal to the mean of rows.

a The number of QTL identified.

b P (in %) was average trait phenotypic variances explained.

In the XZ and XZV hybrids, a large number of epistatic interactions and QEs, which were detected by inclusive composite interval mapping, were observed with the three data sets. The number of epistatic interactions was significantly greater than those of M-QTL. On average, for most traits, the variation explained by E-QTL was larger than that explained by M-QTL (Table 5). The present finding is in agreement with a multitude of previous studies reporting the importance of epistatic interactions in controlling the expression of heterosis (Yu *et al.* 1997; Li *et al.* 2001; Hua *et al.* 2002; Luo *et al.* 2009). Compared to the epistatic effect and QTL identified in the two hybrids, we observed that the number of interactions and the variation explained were different in XZ and XZV hybrids, which indicated that the two hybrids possessed a different genetic structure of yield traits. It should be noted that our results are the same as those of a recent heterosis study in two rice hybrids, which is that the relative contributions of the genetic components varies with traits, and the genetic basis of the two hybrids was different (L. Li *et al.* 2015). Based on the reported method, epistasis was classified into three types in Table 6: (I) two loci with M-QTL; (II) a locus with M-QTL, and a locus without significant M-QTL; and (III) two loci without significant M-QTL (Li *et al.* 2001). In the present study, we collectively detected that 31 of 503 (6.2%) epistatic interactions were type II, and the remaining 472 (93.8%) were type III for yield and yield components in the XZ and XZV hybrid. No type I interactions were observed (Table 6). It was obvious that most digenic interactions that occurred were between complementary loci, with a few detectable main effects also being observed as in previous studies (Yu *et al.* 1997; Luo *et al.* 2001; Li *et al.* 2001; Mei *et al.* 2005; Radoev *et al.* 2008; Luo *et al.* 2009). Like pleiotropic QTL, many E-QTL also showed a pleiotropic effect (Table S7). In addition, statistics of pleiotropic E-QTL in different yield traits revealed that most types of pleiotropic E-QTL occurred among SY, LY and BNP traits in the two hybrids. This result also suggested that the BNP trait was the biggest contributor to yield and yield heterosis, compared with other yield components. This result was consistent with performance of the BNP trait, which showed the biggest MPH percentage in yield components (Table 1).

**Table 6:** Type of epistatic interactions detected in two hybrids

Trait	Data set	Type of epistasis						Sum		Range of the effects			
		I		II		III				XZ		XZV	
		XZ	XZV	XZ	XZV	XZ	XZV	XZ	XZV	Min	Max	Min	Max
SY	RIL	0	0	1	4	11	55	12	59	−2.48	2.64	−4.70	5.43
	BC	0	0	0	1	8	1	8	2	−1.64	1.77	−1.54	0.94
	MPH	0	0	0	1	4	13	4	14	−1.42	1.97	−2.82	2.02
LY	RIL	0	0	1	1	9	54	10	55	−1.05	1.06	−1.88	2.08
	BC	0	0	0	1	10	6	10	7	−0.68	0.85	−0.69	0.83
	MPH	0	0	0	1	4	8	4	9	−0.59	0.85	−1.11	0.83
BNP	RIL	0	0	0	2	15	65	15	67	−0.70	0.63	−1.16	1.27
	BC	0	0	0	0	7	2	7	2	−0.49	0.53	0.17	0.28
	MPH	0	0	0	1	3	23	3	24	−0.43	0.45	−0.73	0.56
BW	RIL	0	0	4	1	32	10	36	11	−0.10	0.10	−0.09	0.10
	BC	0	0	1	2	25	4	26	6	−0.06	0.06	−0.05	0.06
	MPH	0	0	0	0	2	4	2	4	−0.02	0.05	−0.06	0.06
LP	RIL	0	0	4	4	44	18	48	22	−0.53	0.51	−0.52	0.96
	BC	0	0	1	0	26	4	27	4	−0.49	0.29	−0.23	0.23
	MPH	0	0	0	0	4	1	4	1	−0.20	0.22	−0.17	−0.17

Type of epistasis: (I) two loci with main-effect QTL, (II) a locus with main-effect QTL and a locus without significant main-effect QTL, and (III) two loci without significant main-effect QTL. Sum: Total number of epistatic interactions. XZ, XZ hybrid; XZV, XZV hybrid; SY, seed cotton yield; RIL, recombinant inbred line population; BC, backcross population; MPH, mid-parental heterosis; LY, lint yield; BNP, bolls per plant; BW, boll weight; LP, lint percent.

Generally, the genetic basis of heterosis was complicated, involving a large number of loci, their different genetic effects, epistatic interactions, and QTL by environmental interactions. Our research strongly supported previous results in maize hybrids that found that the genetic basis of grain yield heterosis relied on the cumulative effects of dominance, overdominance, and epistasis, and that dominance was more important to heterosis than other genetic effects (Guo *et al.* 2014). In addition, it is worth noting that the importance of QEs was also proportional to the amount of heterosis.

## Supplementary Material

Supporting Information
