# Supplementary material for: Partial Dominance, Overdominance, Epistasis and QTL by Environment Interactions Contribute to Heterosis in Two Upland Cotton Hybrids
Source: G3 (Bethesda). 2015 Dec 29;6(3):499–507. doi: 10.1534/g3.115.025809 (PMC4777113; doi:10.1534/g3.115.025809)
Supplement: Supporting Information [file supp_6_3_499__index.html]

Partial Dominance, Overdominance, Epistasis and QTL by Environment Interactions Contribute to Heterosis in Two Upland Cotton Hybrids — Supporting Information 

# Partial Dominance, Overdominance, Epistasis and QTL by Environment Interactions Contribute to Heterosis in Two Upland Cotton Hybrids

## Supporting Information for Shang *et al.*, 2016

**Files in this Data Supplement:**

- Figure S1 - Locations of QTLs controlling yield and yield components traits identified in two hybrids. (.pdf, 2103 KB)
- Table S3 - Main effects and environmental interactions detected for yield and yield components in BCF1 and BCVF1 populations by inclusive composite interval mapping (.doc, 176 KB)
- Table S4 - Main effects and environmental interactions detected for yield and yield components in two MPH data by inclusive composite interval mapping. (.doc, 88 KB)
- Table S5 - Epistatic effects and environmental interactions detected for yield and yield components in RIL and RILV populations using two-locus analysis by inclusive composite interval mapping. (.doc, 805 KB)
- Table S6 - Epistatic effects and environmental interactions detected for yield and yield components in BCF1 and BCVF1 populations using two-locus analysis by inclusive composite interval mapping (.doc, 242 KB)
- Table S7 - Epistatic effects and environmental interactions detected for yield and yield components in two MPH data using two-locus analysis by inclusive composite interval mapping. (.doc, 172 KB)
- Table S8 - Same QTLs for yield traits compared with results of F2:3 and F2:3 populations. (.doc, 138 KB)
- Table S9 - Genotypes and traits of XZ hybrid used in this work. (.xls, 2456 KB)
- Table S10 - Genotypes and traits of XZV hybrid used in this work. (.xls, 1540 KB)
- Table S1 - QTLs identified for yield and yield components of RIL(V)′, RIL(V), BC(V)F1 and MPH data by composite interval mapping in two hybrids (.doc, 756 KB)
- Table S2 - Main effects and environmental interactions detected for yield and yield components in RIL and RILV populations by inclusive composite interval mapping. (.doc, 217 KB)
