# Supplementary material for: Partial Dominance, Overdominance, Epistasis and QTL by Environment Interactions Contribute to Heterosis in Two Upland Cotton Hybrids
Source: G3 (Bethesda). 2015 Dec 29;6(3):499–507. doi: 10.1534/g3.115.025809 (PMC4777113; doi:10.1534/g3.115.025809)
Supplement: Supporting Information [file supp_g3.115.025809_FigureS1.pdf]

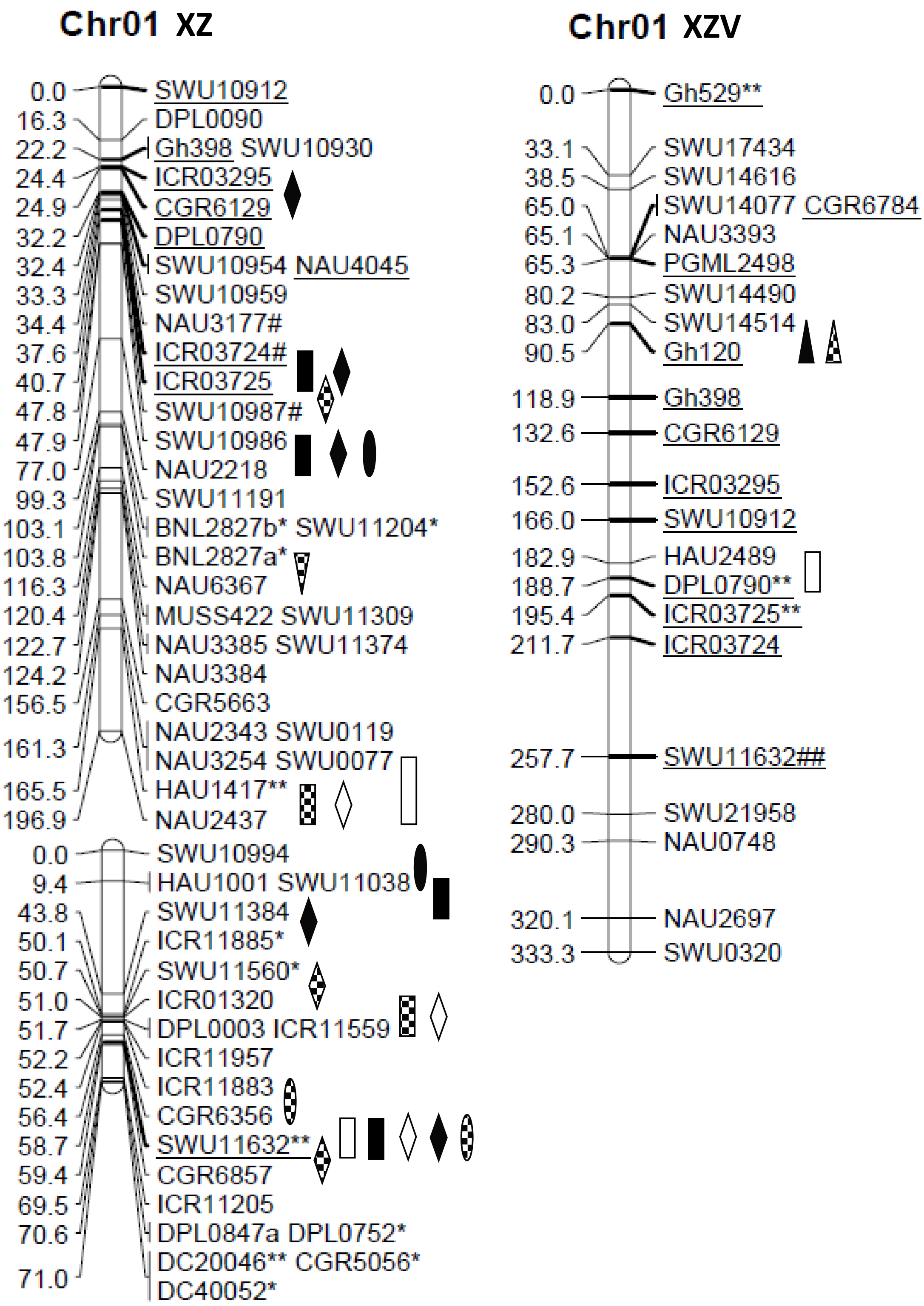
Chr01 XZ
0.0 SWU10912
16.3 DPL0090
22.2 Gh398 SWU10930
24.4 ICR03295
24.9 CGR6129
32.2 DPL0790
32.4 SWU10954 NAU4045
33.3 SWU10959
34.4 NAU3177#
37.6 ICR03724#
40.7 ICR03725
47.8 SWU10987#
47.9 SWU10986
77.0 NAU2218
99.3 SWU11191
103.1 BNL2827b* SWU11204*
103.8 BNL2827a*
116.3 NAU6367
120.4 MUSS422 SWU11309
122.7 NAU3385 SWU11374
124.2 NAU3384
156.5 CGR5663
161.3 NAU2343 SWU0119
NAU3254 SWU0077
165.5 HAU1417**
196.9 NAU2437
0.0 SWU10994
9.4 HAU1001 SWU11038
43.8 SWU11384
50.1 ICR11885*
50.7 SWU11560*
51.0 ICR01320
51.7 DPL0003 ICR11559
52.2 ICR11957
52.4 ICR11883
56.4 CGR6356
58.7 SWU11632**
59.4 CGR6857
69.5 ICR11205
70.6 DPL0847a DPL0752*
71.0 DC20046** CGR5056*
DC40052*
Chr01 XZV
0.0 Gh529**
33.1 SWU17434
38.5 SWU14616
65.0 SWU14077 CGR6784
65.1 NAU3393
65.3 PGML2498
80.2 SWU14490
83.0 SWU14514
90.5 Gh120
118.9 Gh398
132.6 CGR6129
152.6 ICR03295
166.0 SWU10912
182.9 HAU2489
188.7 DPL0790**
195.4 ICR03725**
211.7 ICR03724
257.7 SWU11632##
280.0 SWU21958
290.3 NAU0748
320.1 NAU2697
333.3 SWU0320

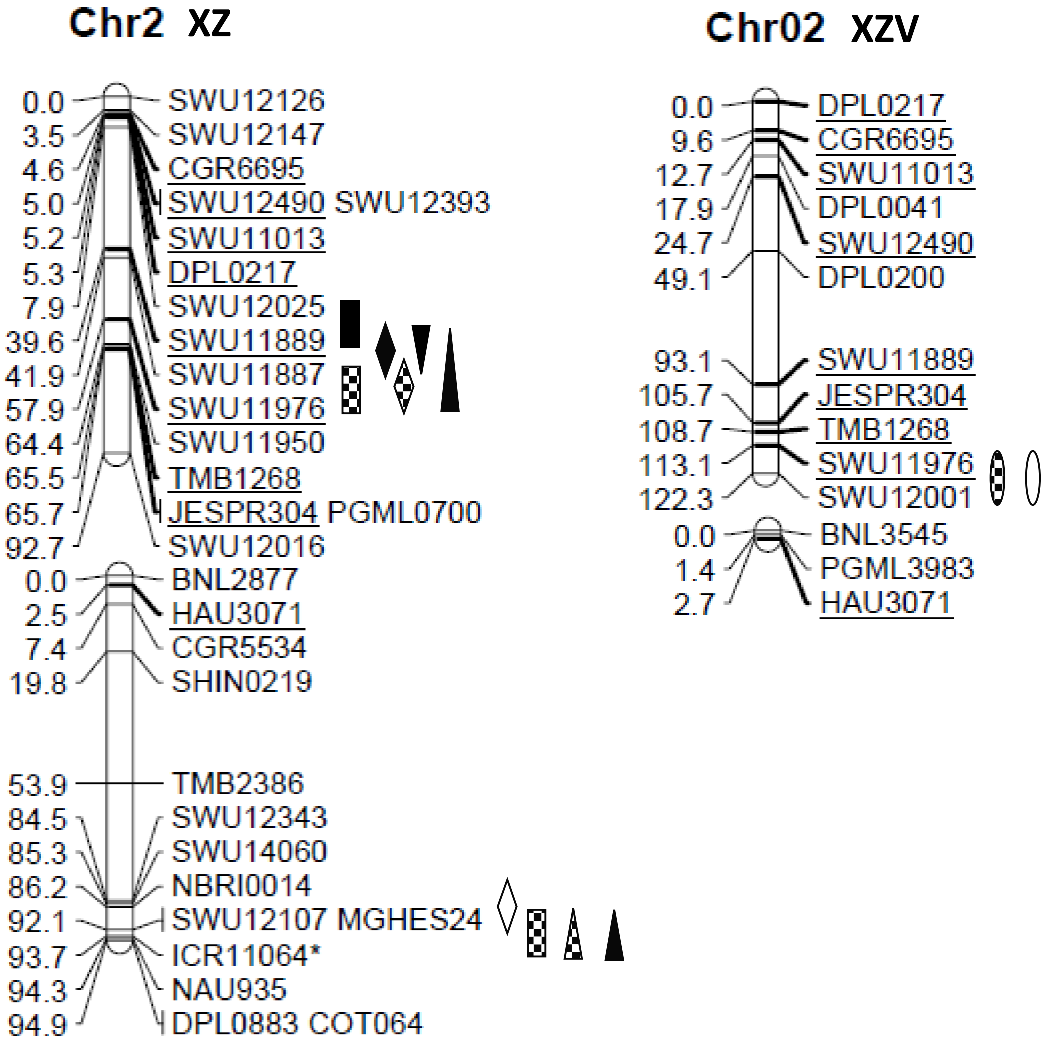

Chr2 XZ
0.0 SWU12126
3.5 SWU12147
4.6 CGR6695
5.0 SWU12490 SWU12393
5.2 SWU11013
5.3 DPL0217
7.9 SWU12025
39.6 SWU11889
41.9 SWU11887
57.9 SWU11976
64.4 SWU11950
65.5 TMB1268
65.7 JESPR304 PGML0700
92.7 SWU12016
0.0 BNL2877
2.5 HAU3071
7.4 CGR5534
19.8 SHIN0219
53.9 TMB2386
84.5 SWU12343
85.3 SWU14060
86.2 NBRI0014
92.1 SWU12107 MGHES24
93.7 ICR11064*
94.3 NAU935
94.9 DPL0883 COT064
Chr02 XZV
0.0 DPL0217
9.6 CGR6695
12.7 SWU11013
17.9 DPL0041
24.7 SWU12490
49.1 DPL0200
93.1 SWU11889
105.7 JESPR304
108.7 TMB1268
113.1 SWU11976
122.3 SWU12001
0.0 BNL3545
1.4 PGML3983
2.7 HAU3071

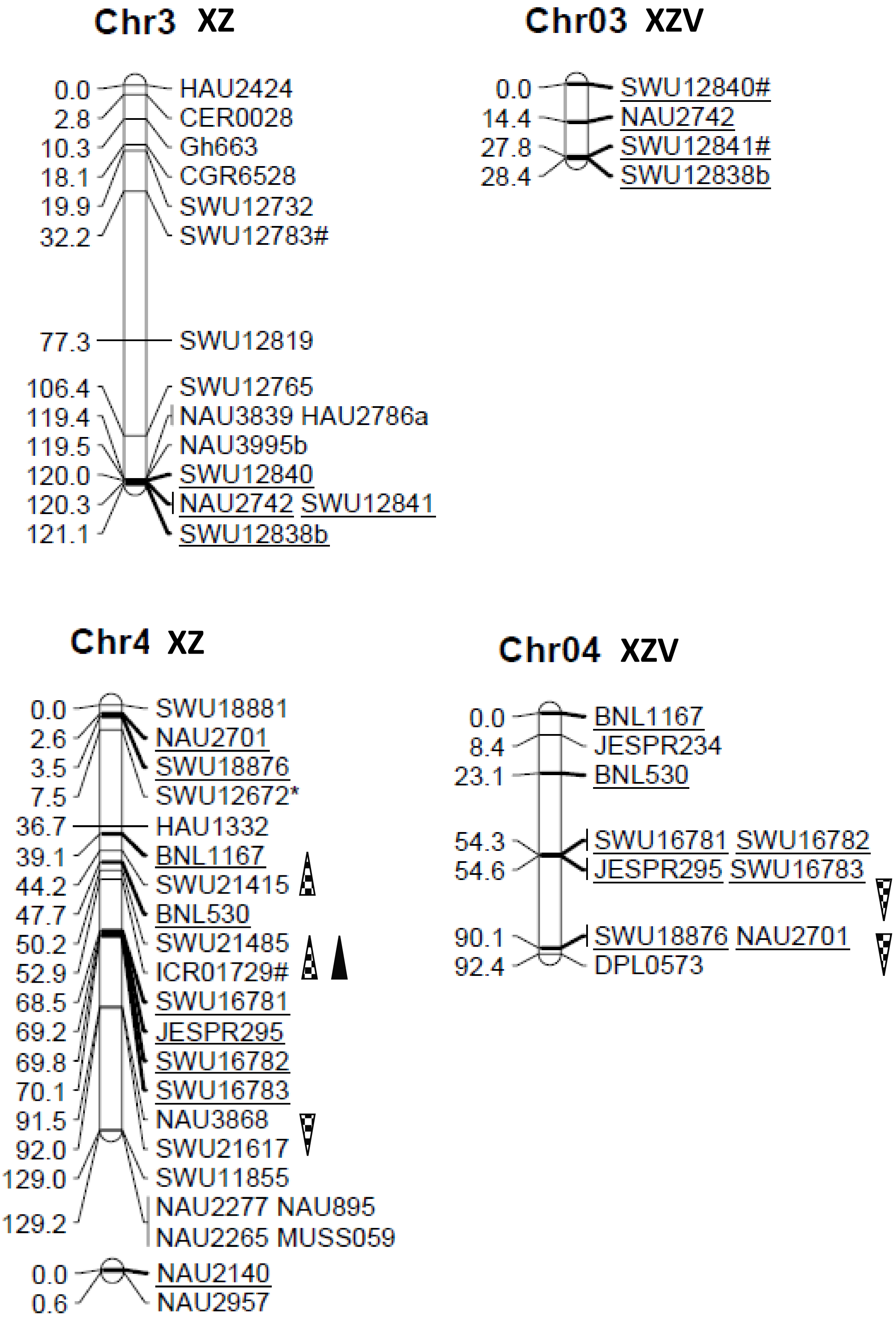
Chr3 XZ
0.0 HAU2424
2.8 CER0028
10.3 Gh663
18.1 CGR6528
19.9 SWU12732
32.2 SWU12783#
77.3 SWU12819
106.4 SWU12765
119.4 NAU3839 HAU2786a
119.5 NAU3995b
120.0 SWU12840
120.3 NAU2742 SWU12841
121.1 SWU12838b
Chr03 XZV
0.0 SWU12840#
14.4 NAU2742
27.8 SWU12841#
28.4 SWU12838b
Chr4 XZ
0.0 SWU18881
2.6 NAU2701
3.5 SWU18876
7.5 SWU12672*
36.7 HAU1332
39.1 BNL1167
44.2 SWU21415
47.7 BNL530
50.2 SWU21485
52.9 ICR01729#
68.5 SWU16781
69.2 JESPR295
69.8 SWU16782
70.1 SWU16783
91.5 NAU3868
92.0 SWU21617
129.0 SWU11855
129.2 NAU2277 NAU895
NAU2265 MUSS059
0.0 NAU2140
0.6 NAU2957
Chr04 XZV
0.0 BNL1167
8.4 JESPR234
23.1 BNL530
54.3 SWU16781 SWU16782
54.6 JESPR295 SWU16783
90.1 SWU18876 NAU2701
92.4 DPL0573

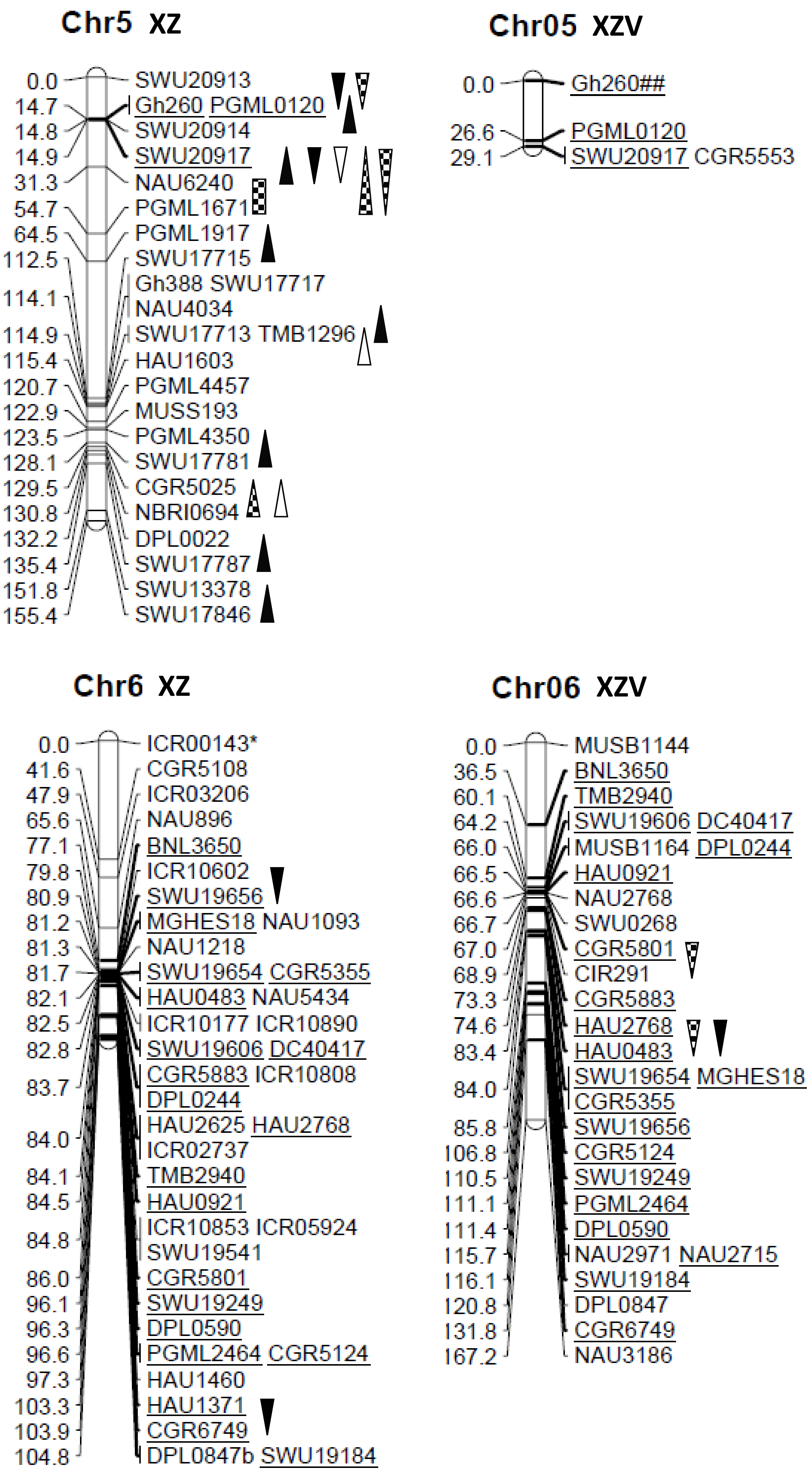
Chr5 XZ
0.0 SWU20913
14.7 Gh260 PGML0120
14.8 SWU20914
14.9 SWU20917
31.3 NAU6240
54.7 PGML1671
64.5 PGML1917
112.5 SWU17715
Gh388 SWU17717
114.1 NAU4034
114.9 SWU17713 TMB1296
115.4 HAU1603
120.7 PGML4457
122.9 MUSS193
123.5 PGML4350
128.1 SWU17781
129.5 CGR5025
130.8 NBRI0694
132.2 DPL0022
135.4 SWU17787
151.8 SWU13378
155.4 SWU17846
Chr05 XZV
0.0 Gh260##
26.6 PGML0120
29.1 SWU20917 CGR5553
Chr6 XZ
0.0 ICR00143*
41.6 CGR5108
47.9 ICR03206
65.6 NAU896
77.1 BNL3650
79.8 ICR10602
80.9 SWU19656
81.2 MGHES18 NAU1093
81.3 NAU1218
81.7 SWU19654 CGR5355
82.1 HAU0483 NAU5434
82.5 ICR10177 ICR10890
82.8 SWU19606 DC40417
CGR5883 ICR10808
83.7 DPL0244
HAU2625 HAU2768
84.0 ICR02737
84.1 TMB2940
84.5 HAU0921
ICR10853 ICR05924
84.8 SWU19541
86.0 CGR5801
96.1 SWU19249
96.3 DPL0590
96.6 PGML2464 CGR5124
97.3 HAU1460
103.3 HAU1371
103.9 CGR6749
104.8 DPL0847b SWU19184
Chr06 XZV
0.0 MUSB1144
36.5 BNL3650
60.1 TMB2940
64.2 SWU19606 DC40417
66.0 MUSB1164 DPL0244
66.5 HAU0921
66.6 NAU2768
66.7 SWU0268
67.0 CGR5801
68.9 CIR291
73.3 CGR5883
74.6 HAU2768
83.4 HAU0483
SWU19654 MGHES18
84.0 CGR5355
85.8 SWU19656
106.8 CGR5124
110.5 SWU19249
111.1 PGML2464
111.4 DPL0590
115.7 NAU2971 NAU2715
116.1 SWU19184
120.8 DPL0847
131.8 CGR6749
167.2 NAU3186

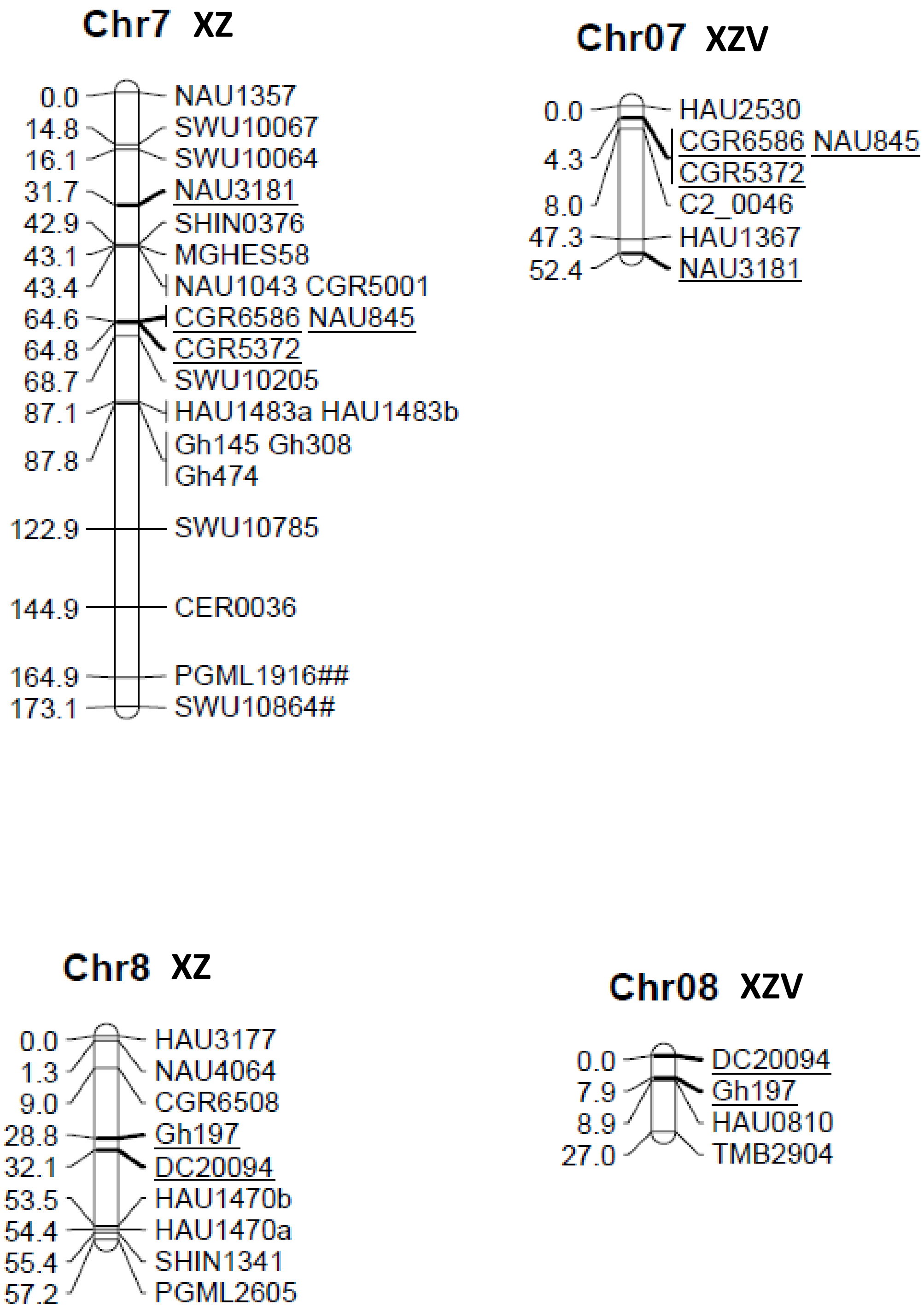

Chr7 XZ
0.0 NAU1357
14.8 SWU10067
16.1 SWU10064
31.7 NAU3181
42.9 SHIN0376
43.1 MGHES58
43.4 NAU1043 CGR5001
64.6 CGR6586 NAU845
64.8 CGR5372
68.7 SWU10205
87.1 HAU1483a HAU1483b
87.8 Gh145 Gh308 Gh474
122.9 SWU10785
144.9 CER0036
164.9 PGML1916##
173.1 SWU10864#
Chr07 XZV
0.0 HAU2530
4.3 CGR6586 NAU845 CGR5372
8.0 C2_0046
47.3 HAU1367
52.4 NAU3181
Chr8 XZ
0.0 HAU3177
1.3 NAU4064
9.0 CGR6508
28.8 Gh197
32.1 DC20094
53.5 HAU1470b
54.4 HAU1470a
55.4 SHIN1341
57.2 PGML2605
Chr08 XZV
0.0 DC20094
7.9 Gh197
8.9 HAU0810
27.0 TMB2904

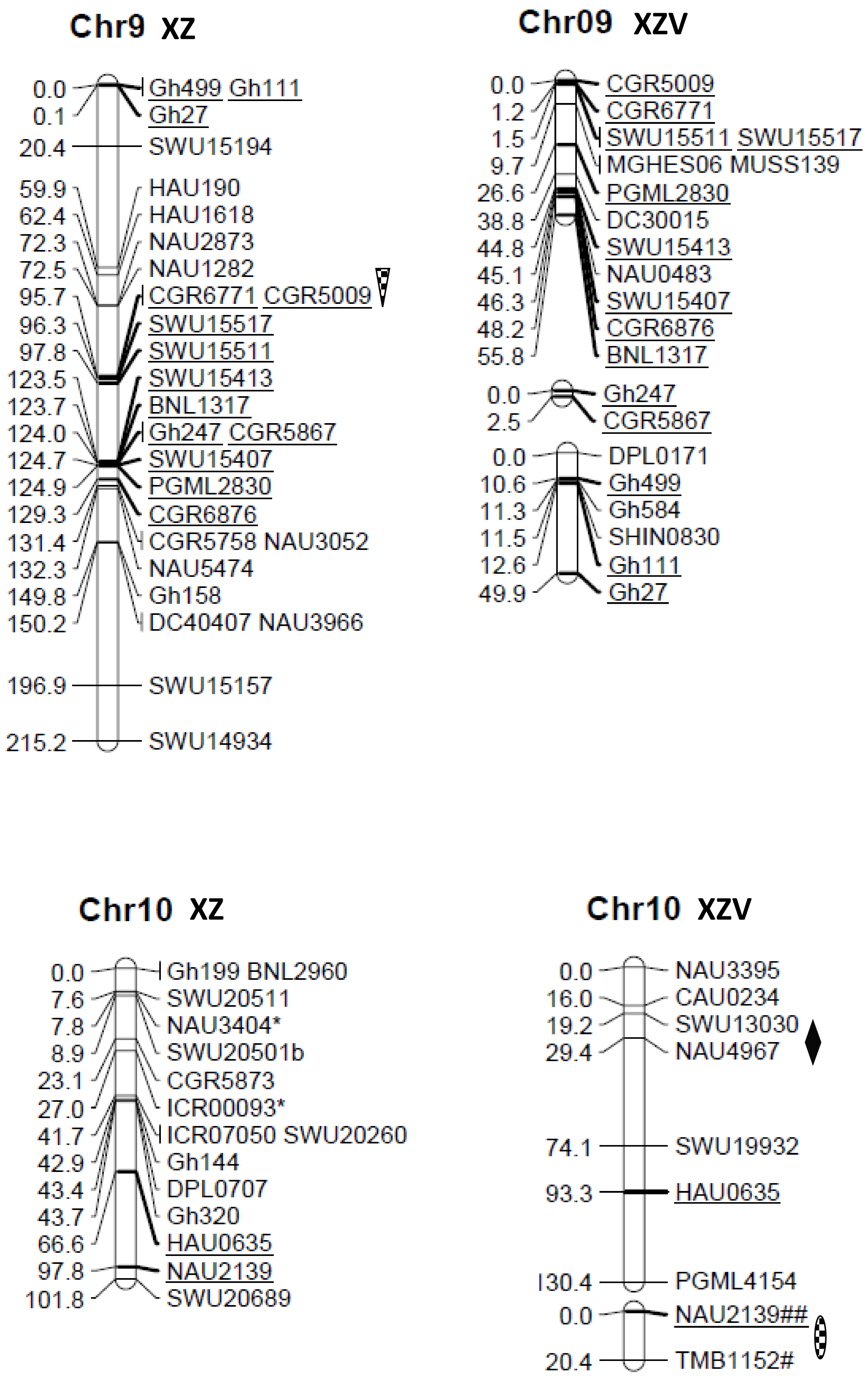

Chr9 XZ
0.0 Gh499 Gh111
0.1 Gh27
20.4 SWU15194
59.9 HAU190
62.4 HAU1618
72.3 NAU2873
72.5 NAU1282
95.7 CGR6771 CGR5009
96.3 SWU15517
97.8 SWU15511
123.5 SWU15413
123.7 BNL1317
124.0 Gh247 CGR5867
124.7 SWU15407
124.9 PGML2830
129.3 CGR6876
131.4 CGR5758 NAU3052
132.3 NAU5474
149.8 Gh158
150.2 DC40407 NAU3966
196.9 SWU15157
215.2 SWU14934
Chr09 XZV
0.0 CGR5009
1.2 CGR6771
1.5 SWU15511 SWU15517
9.7 MGHES06 MUSS139
26.6 PGML2830
38.8 DC30015
44.8 SWU15413
45.1 NAU0483
46.3 SWU15407
48.2 CGR6876
55.8 BNL1317
0.0 Gh247
2.5 CGR5867
0.0 DPL0171
10.6 Gh499
11.3 Gh584
11.5 SHIN0830
12.6 Gh111
49.9 Gh27
Chr10 XZ
0.0 Gh199 BNL2960
7.6 SWU20511
7.8 NAU3404*
8.9 SWU20501b
23.1 CGR5873
27.0 ICR00093*
41.7 ICR07050 SWU20260
42.9 Gh144
43.4 DPL0707
43.7 Gh320
66.6 HAU0635
97.8 NAU2139
101.8 SWU20689
Chr10 XZV
0.0 NAU3395
16.0 CAU0234
19.2 SWU13030
29.4 NAU4967
74.1 SWU19932
93.3 HAU0635
130.4 PGML4154
0.0 NAU2139##
20.4 TMB1152#

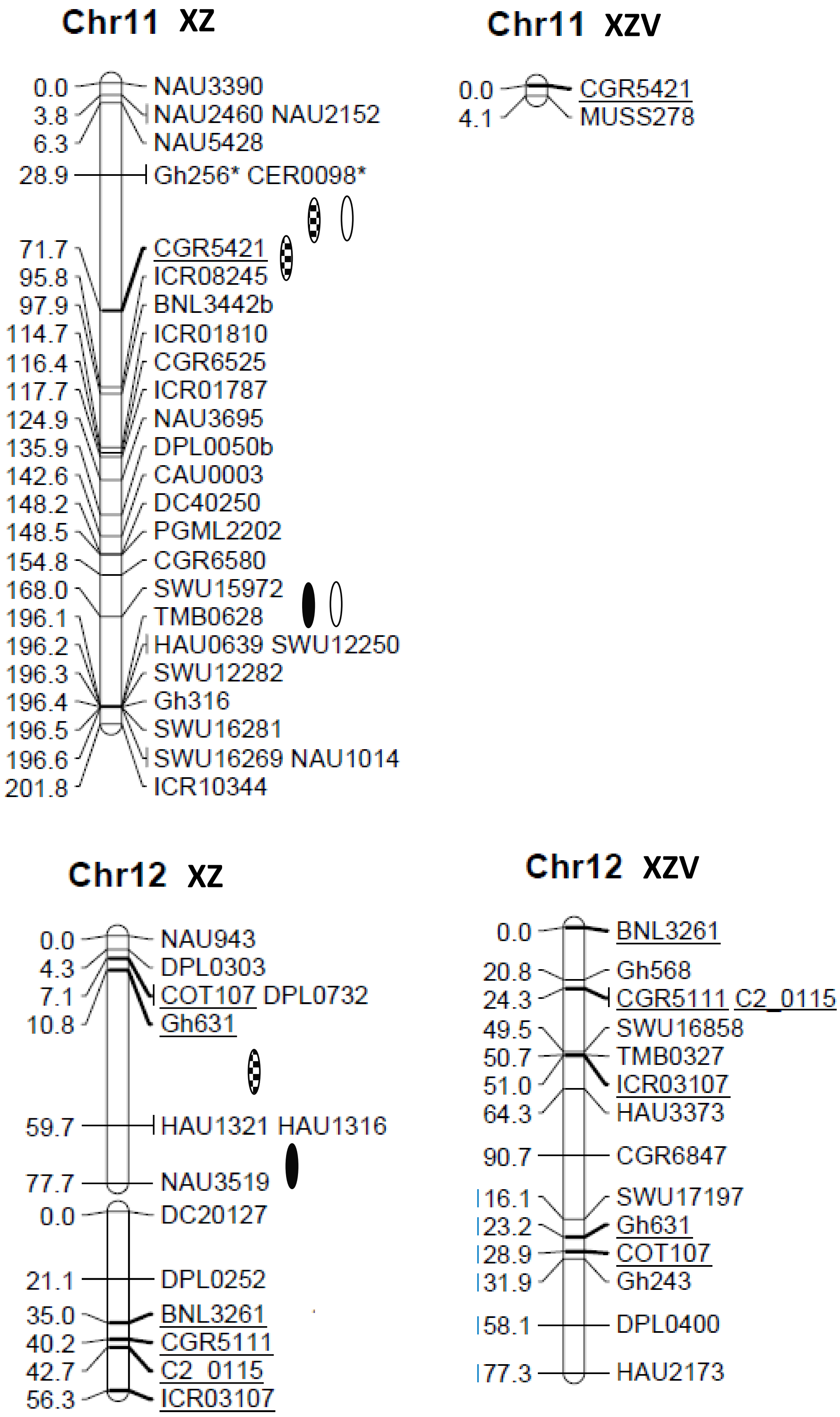

Chr11 XZ
0.0 NAU3390
3.8 NAU2460 NAU2152
6.3 NAU5428
28.9 Gh256* CER0098*
71.7 CGR5421
95.8 ICR08245
97.9 BNL3442b
114.7 ICR01810
116.4 CGR6525
117.7 ICR01787
124.9 NAU3695
135.9 DPL0050b
142.6 CAU0003
148.2 DC40250
148.5 PGML2202
154.8 CGR6580
168.0 SWU15972
196.1 TMB0628
196.2 HAU0639 SWU12250
196.3 SWU12282
196.4 Gh316
196.5 SWU16281
196.6 SWU16269 NAU1014
201.8 ICR10344
Chr11 XZV
0.0 CGR5421
4.1 MUSS278
Chr12 XZ
0.0 NAU943
4.3 DPL0303
7.1 COT107 DPL0732
10.8 Gh631
59.7 HAU1321 HAU1316
77.7 NAU3519
0.0 DC20127
21.1 DPL0252
35.0 BNL3261
40.2 CGR5111
42.7 C2_0115
56.3 ICR03107
Chr12 XZV
0.0 BNL3261
20.8 Gh568
24.3 CGR5111 C2_0115
49.5 SWU16858
50.7 TMB0327
51.0 ICR03107
64.3 HAU3373
90.7 CGR6847
116.1 SWU17197
123.2 Gh631
128.9 COT107
131.9 Gh243
158.1 DPL0400
177.3 HAU2173

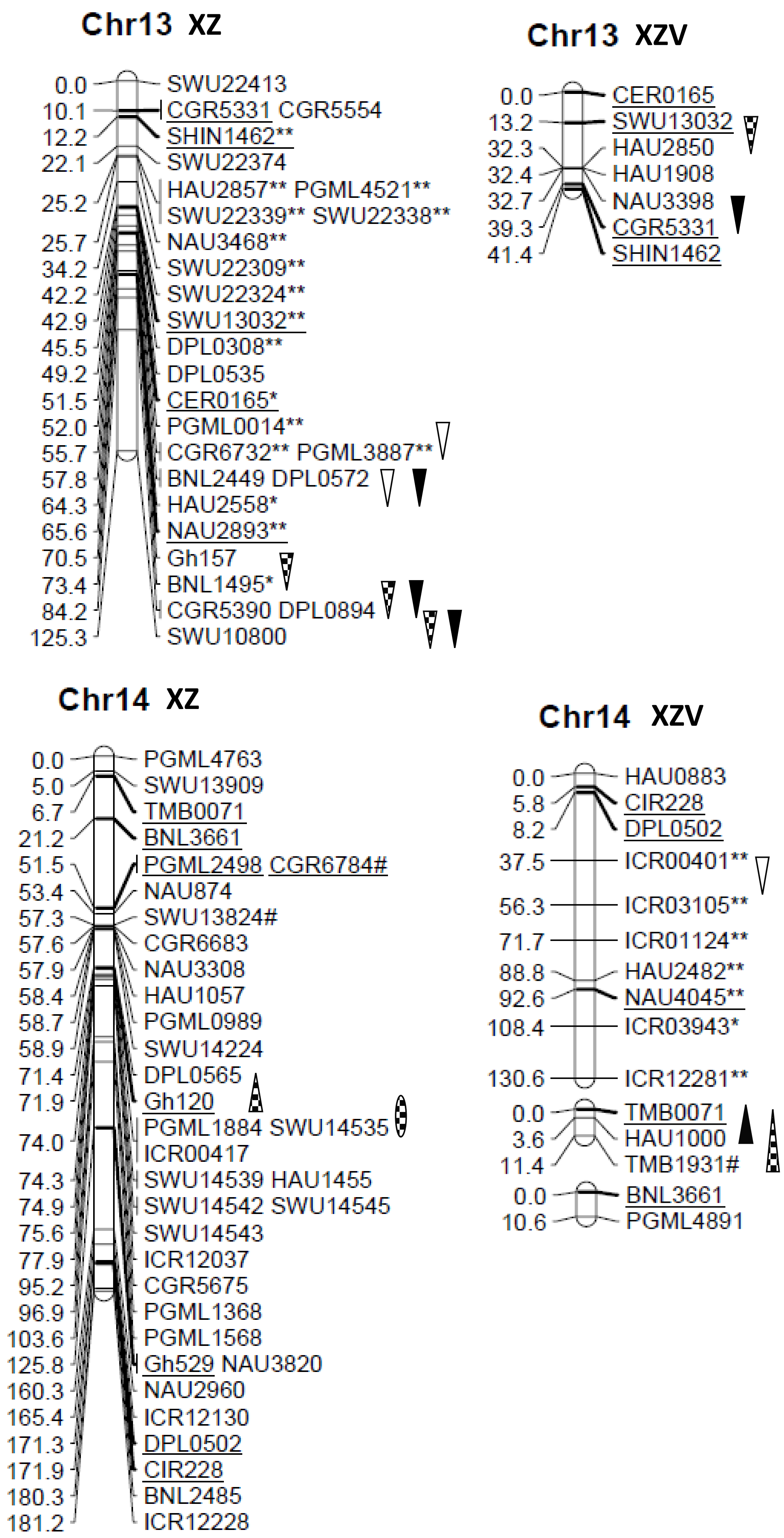

Chr13 XZ
0.0 SWU22413
10.1 CGR5331 CGR5554
12.2 SHIN1462**
22.1 SWU22374
25.2 HAU2857** PGML4521** SWU22339** SWU22338**
25.7 NAU3468**
34.2 SWU22309**
42.2 SWU22324**
42.9 SWU13032**
45.5 DPL0308**
49.2 DPL0535
51.5 CER0165*
52.0 PGML0014**
55.7 CGR6732** PGML3887**
57.8 BNL2449 DPL0572
64.3 HAU2558*
65.6 NAU2893**
70.5 Gh157
73.4 BNL1495*
84.2 CGR5390 DPL0894
125.3 SWU10800
Chr13 XZV
0.0 CER0165
13.2 SWU13032
32.3 HAU2850
32.4 HAU1908
32.7 NAU3398
39.3 CGR5331
41.4 SHIN1462
Chr14 XZ
0.0 PGML4763
5.0 SWU13909
6.7 TMB0071
21.2 BNL3661
51.5 PGML2498 CGR6784#
53.4 NAU874
57.3 SWU13824#
57.6 CGR6683
57.9 NAU3308
58.4 HAU1057
58.7 PGML0989
58.9 SWU14224
71.4 DPL0565
71.9 Gh120
74.0 PGML1884 SWU14535 ICR00417
74.3 SWU14539 HAU1455
74.9 SWU14542 SWU14545
75.6 SWU14543
77.9 ICR12037
95.2 CGR5675
96.9 PGML1368
103.6 PGML1568
125.8 Gh529 NAU3820
160.3 NAU2960
165.4 ICR12130
171.3 DPL0502
171.9 CIR228
180.3 BNL2485
181.2 ICR12228
Chr14 XZV
0.0 HAU0883
5.8 CIR228
8.2 DPL0502
37.5 ICR00401**
56.3 ICR03105**
71.7 ICR01124**
88.8 HAU2482**
92.6 NAU4045**
108.4 ICR03943*
130.6 ICR12281**
0.0 TMB0071
3.6 HAU1000
11.4 TMB1931#
0.0 BNL3661
10.6 PGML4891

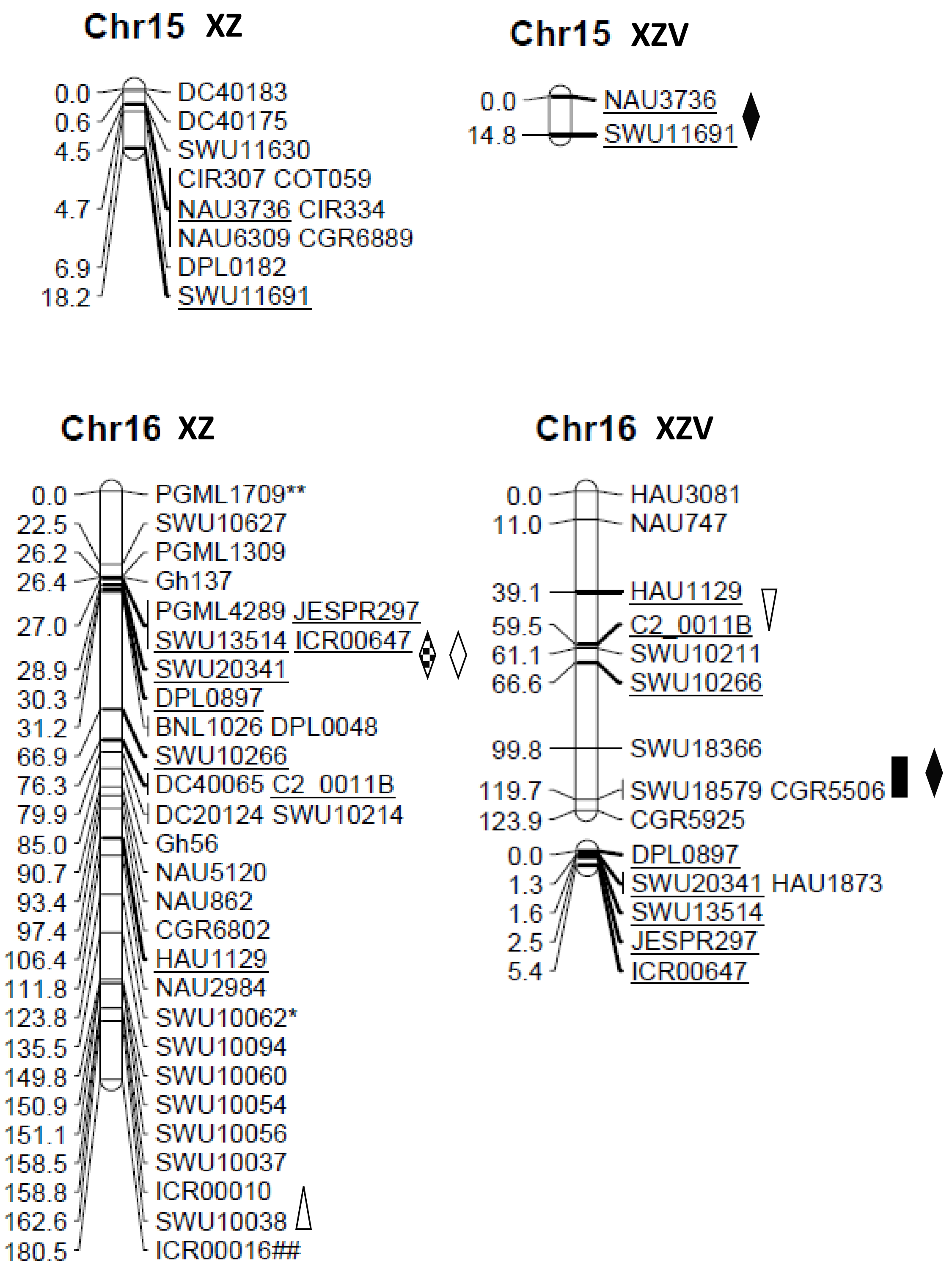
Chr15 XZ
0.0 DC40183
0.6 DC40175
4.5 SWU11630
CIR307 COT059
4.7 NAU3736 CIR334
NAU6309 CGR6889
6.9 DPL0182
18.2 SWU11691
Chr15 XZV
0.0 NAU3736
14.8 SWU11691
Chr16 XZ
0.0 PGML1709**
22.5 SWU10627
26.2 PGML1309
26.4 Gh137
PGML4289 JESPR297
27.0 SWU13514 ICR00647
28.9 SWU20341
30.3 DPL0897
31.2 BNL1026 DPL0048
66.9 SWU10266
76.3 DC40065 C2_0011B
79.9 DC20124 SWU10214
85.0 Gh56
90.7 NAU5120
93.4 NAU862
97.4 CGR6802
106.4 HAU1129
111.8 NAU2984
123.8 SWU10062*
135.5 SWU10094
149.8 SWU10060
150.9 SWU10054
151.1 SWU10056
158.5 SWU10037
158.8 ICR00010
162.6 SWU10038
180.5 ICR00016##
Chr16 XZV
0.0 HAU3081
11.0 NAU747
39.1 HAU1129
59.5 C2_0011B
61.1 SWU10211
66.6 SWU10266
99.8 SWU18366
119.7 SWU18579 CGR5506
123.9 CGR5925
0.0 DPL0897
1.3 SWU20341 HAU1873
1.6 SWU13514
2.5 JESPR297
5.4 ICR00647

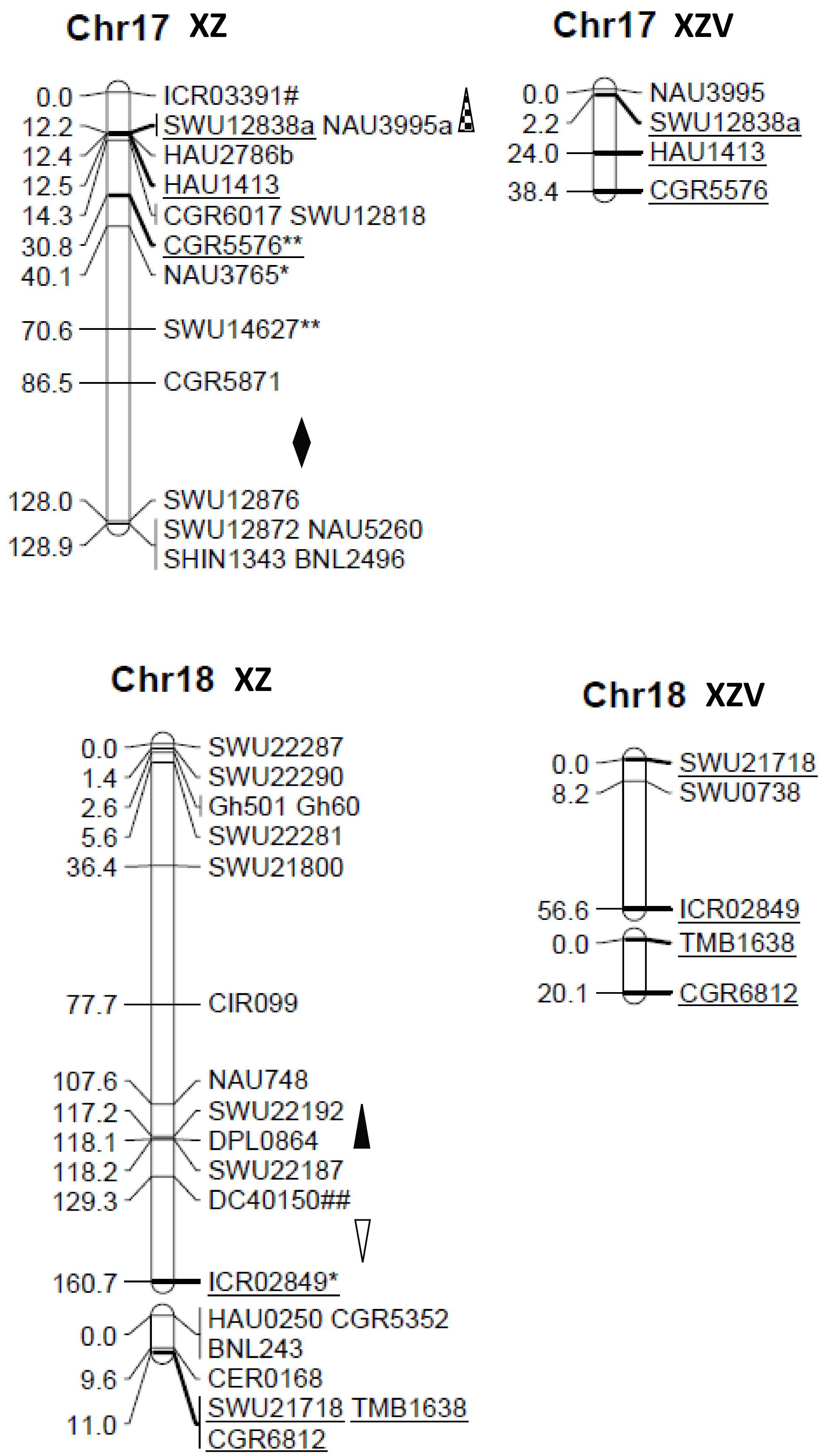

Chr17 XZ
0.0 ICR03391#
12.2 SWU12838a NAU3995a
12.4 HAU2786b
12.5 HAU1413
14.3 CGR6017 SWU12818
30.8 CGR5576**
40.1 NAU3765*
70.6 SWU14627**
86.5 CGR5871
128.0 SWU12876
128.9 SWU12872 NAU5260
SHIN1343 BNL2496
Chr17 XZV
0.0 NAU3995
2.2 SWU12838a
24.0 HAU1413
38.4 CGR5576
Chr18 XZ
0.0 SWU22287
1.4 SWU22290
2.6 Gh501 Gh60
5.6 SWU22281
36.4 SWU21800
77.7 CIR099
107.6 NAU748
117.2 SWU22192
118.1 DPL0864
118.2 SWU22187
129.3 DC40150##
160.7 ICR02849*
0.0 HAU0250 CGR5352
BNL243
9.6 CER0168
11.0 SWU21718 TMB1638
CGR6812
Chr18 XZV
0.0 SWU21718
8.2 SWU0738
56.6 ICR02849
0.0 TMB1638
20.1 CGR6812

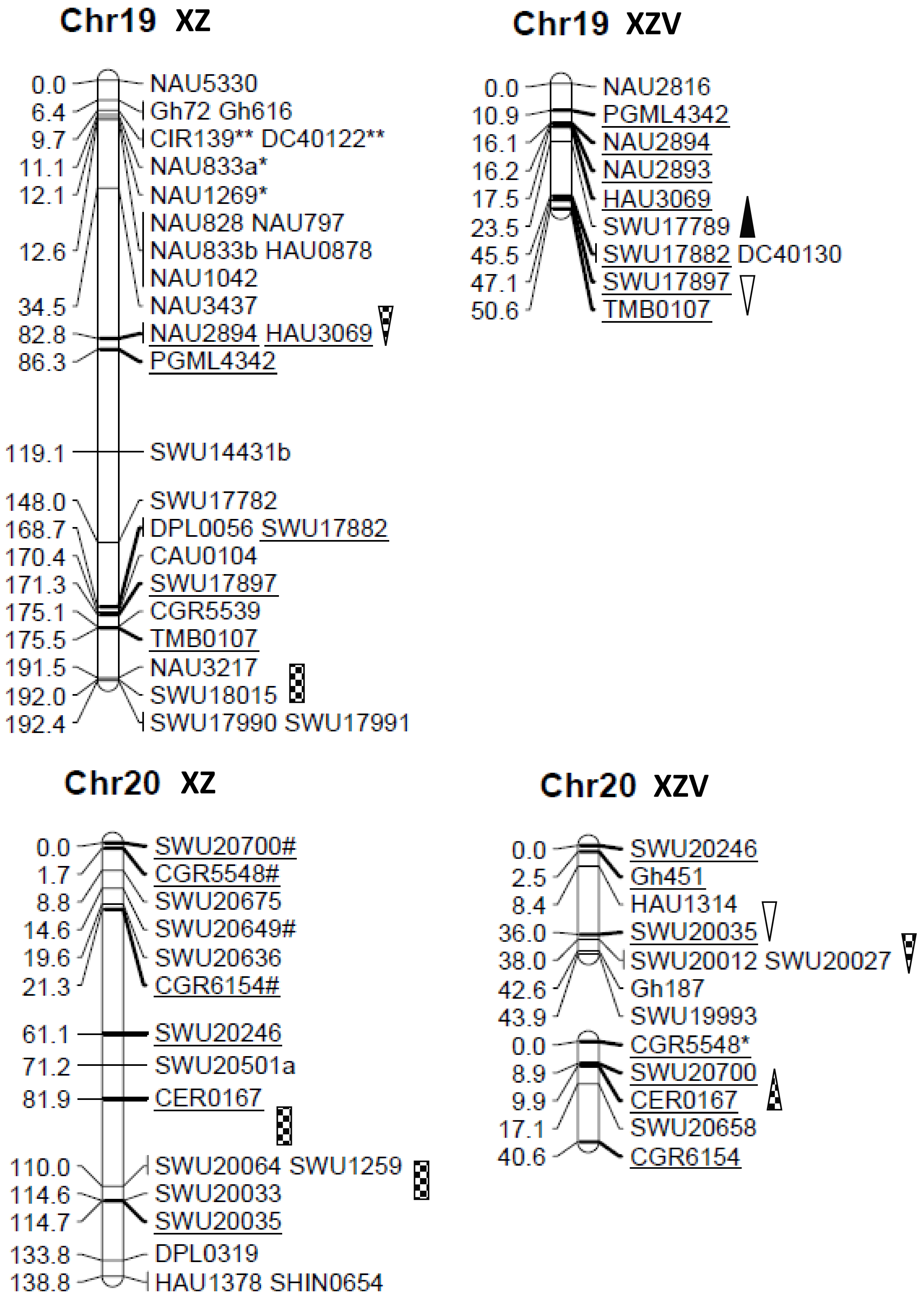
Chr19 XZ
0.0 NAU5330
6.4 Gh72 Gh616
9.7 CIR139** DC40122**
11.1 NAU833a*
12.1 NAU1269*
NAU828 NAU797
12.6 NAU833b HAU0878
NAU1042
34.5 NAU3437
82.8 NAU2894 HAU3069
86.3 PGML4342
119.1 SWU14431b
148.0 SWU17782
168.7 DPL0056 SWU17882
170.4 CAU0104
171.3 SWU17897
175.1 CGR5539
175.5 TMB0107
191.5 NAU3217
192.0 SWU18015
192.4 SWU17990 SWU17991
Chr19 XZV
0.0 NAU2816
10.9 PGML4342
16.1 NAU2894
16.2 NAU2893
17.5 HAU3069
23.5 SWU17789
45.5 SWU17882 DC40130
47.1 SWU17897
50.6 TMB0107
Chr20 XZ
0.0 SWU20700#
1.7 CGR5548#
8.8 SWU20675
14.6 SWU20649#
19.6 SWU20636
21.3 CGR6154#
61.1 SWU20246
71.2 SWU20501a
81.9 CER0167
110.0 SWU20064 SWU1259
114.6 SWU20033
114.7 SWU20035
133.8 DPL0319
138.8 HAU1378 SHIN0654
Chr20 XZV
0.0 SWU20246
2.5 Gh451
8.4 HAU1314
36.0 SWU20035
38.0 SWU20012 SWU20027
42.6 Gh187
43.9 SWU19993
0.0 CGR5548*
8.9 SWU20700
9.9 CER0167
17.1 SWU20658
40.6 CGR6154

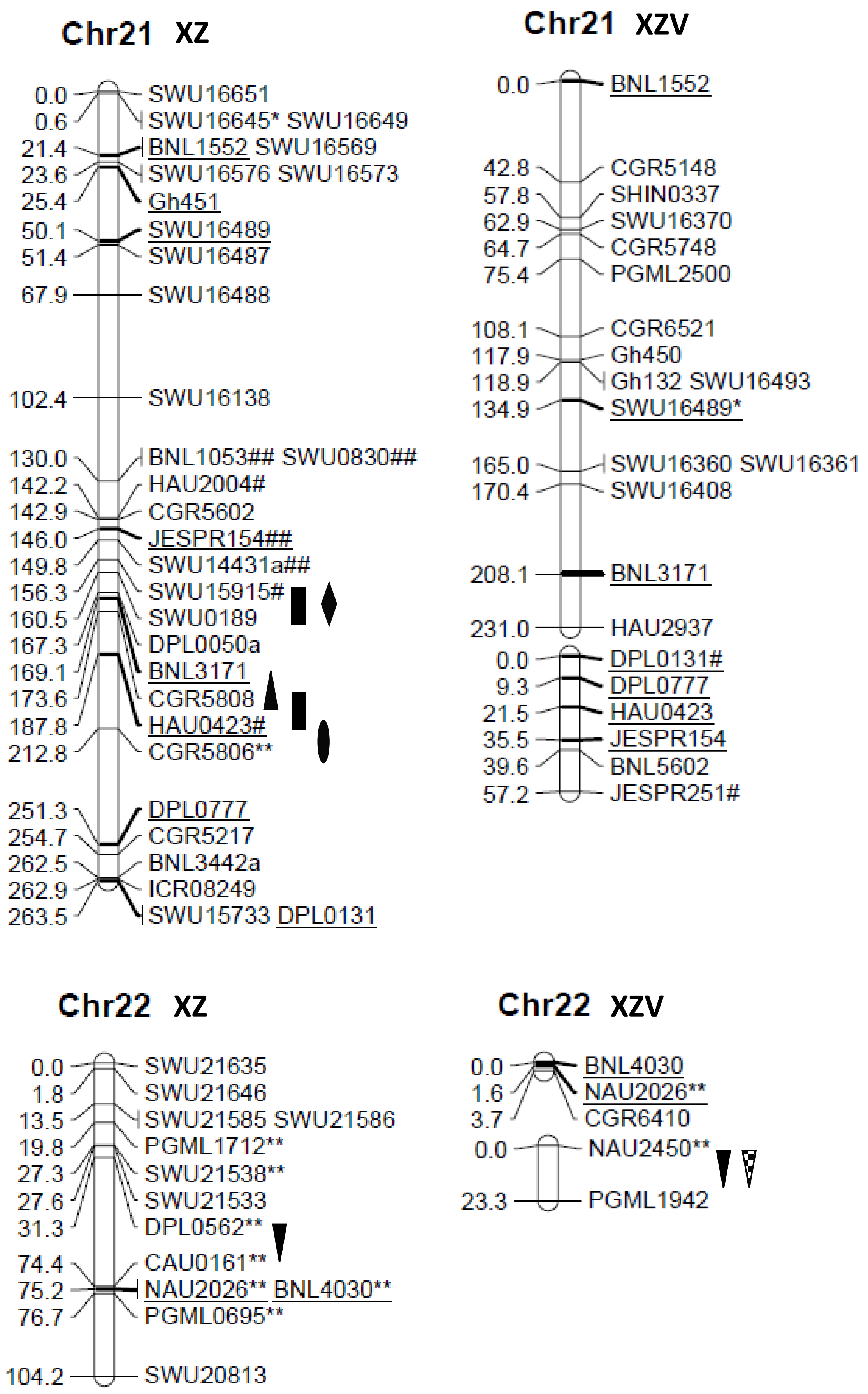
Chr21 XZ
0.0 SWU16651
0.6 SWU16645* SWU16649
21.4 BNL1552 SWU16569
23.6 SWU16576 SWU16573
25.4 Gh451
50.1 SWU16489
51.4 SWU16487
67.9 SWU16488
102.4 SWU16138
130.0 BNL1053## SWU0830##
142.2 HAU2004#
142.9 CGR5602
146.0 JESPR154##
149.8 SWU14431a##
156.3 SWU15915#
160.5 SWU0189
167.3 DPL0050a
169.1 BNL3171
173.6 CGR5808
187.8 HAU0423#
212.8 CGR5806**
251.3 DPL0777
254.7 CGR5217
262.5 BNL3442a
262.9 ICR08249
263.5 SWU15733 DPL0131
Chr21 XZV
0.0 BNL1552
42.8 CGR5148
57.8 SHIN0337
62.9 SWU16370
64.7 CGR5748
75.4 PGML2500
108.1 CGR6521
117.9 Gh450
118.9 Gh132 SWU16493
134.9 SWU16489*
165.0 SWU16360 SWU16361
170.4 SWU16408
208.1 BNL3171
231.0 HAU2937
0.0 DPL0131#
9.3 DPL0777
21.5 HAU0423
35.5 JESPR154
39.6 BNL5602
57.2 JESPR251#
Chr22 XZ
0.0 SWU21635
1.8 SWU21646
13.5 SWU21585 SWU21586
19.8 PGML1712**
27.3 SWU21538**
27.6 SWU21533
31.3 DPL0562**
74.4 CAU0161**
75.2 NAU2026** BNL4030**
76.7 PGML0695**
104.2 SWU20813
Chr22 XZV
0.0 BNL4030
1.6 NAU2026**
3.7 CGR6410
0.0 NAU2450**
23.3 PGML1942

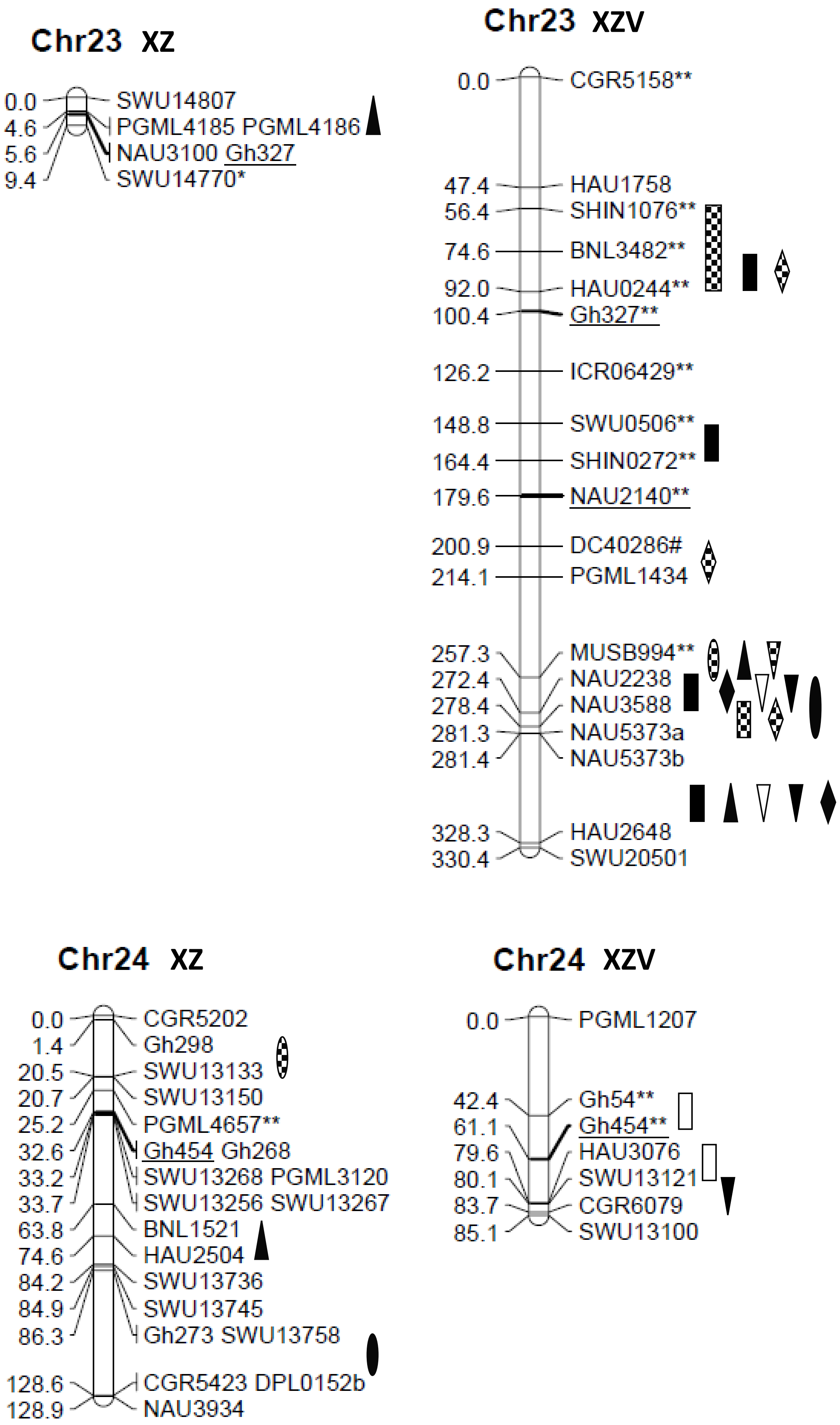

Chr23 XZ
0.0 SWU14807
4.6 PGML4185 PGML4186
5.6 NAU3100 Gh327
9.4 SWU14770*
Chr23 XZV
0.0 CGR5158**
47.4 HAU1758
56.4 SHIN1076**
74.6 BNL3482**
92.0 HAU0244**
100.4 Gh327**
126.2 ICR06429**
148.8 SWU0506**
164.4 SHIN0272**
179.6 NAU2140**
200.9 DC40286#
214.1 PGML1434
257.3 MUSB994**
272.4 NAU2238
278.4 NAU3588
281.3 NAU5373a
281.4 NAU5373b
328.3 HAU2648
330.4 SWU20501
Chr24 XZ
0.0 CGR5202
1.4 Gh298
20.5 SWU13133
20.7 SWU13150
25.2 PGML4657**
32.6 Gh454 Gh268
33.2 SWU13268 PGML3120
33.7 SWU13256 SWU13267
63.8 BNL1521
74.6 HAU2504
84.2 SWU13736
84.9 SWU13745
86.3 Gh273 SWU13758
128.6 CGR5423 DPL0152b
128.9 NAU3934
Chr24 XZV
0.0 PGML1207
42.4 Gh54**
61.1 Gh454**
79.6 HAU3076
80.1 SWU13121
83.7 CGR6079
85.1 SWU13100

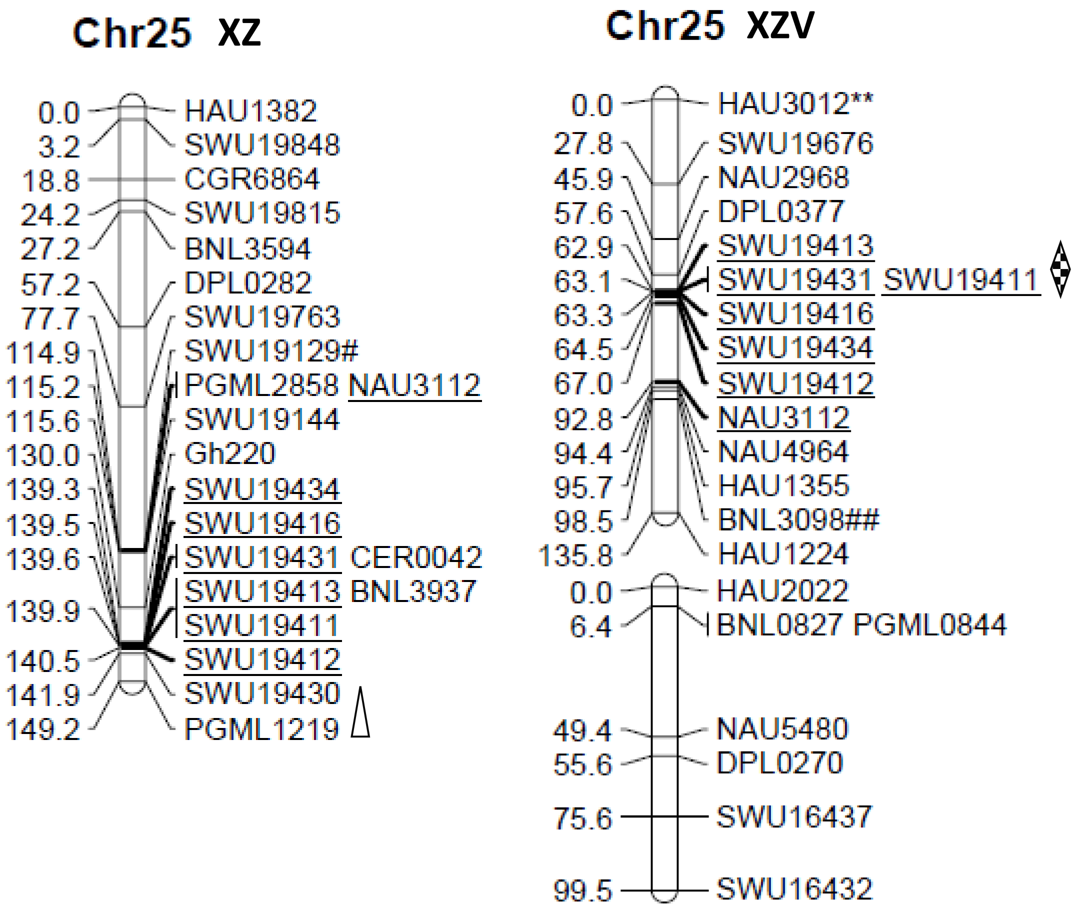

Chr25 XZ
0.0 HAU1382
3.2 SWU19848
18.8 CGR6864
24.2 SWU19815
27.2 BNL3594
57.2 DPL0282
77.7 SWU19763
114.9 SWU19129#
115.2 PGML2858 NAU3112
115.6 SWU19144
130.0 Gh220
139.3 SWU19434
139.5 SWU19416
139.6 SWU19431 CER0042
139.9 SWU19413 BNL3937
SWU19411
140.5 SWU19412
141.9 SWU19430
149.2 PGML1219
Chr25 XZV
0.0 HAU3012**
27.8 SWU19676
45.9 NAU2968
57.6 DPL0377
62.9 SWU19413
63.1 SWU19431 SWU19411
63.3 SWU19416
64.5 SWU19434
67.0 SWU19412
92.8 NAU3112
94.4 NAU4964
95.7 HAU1355
98.5 BNL3098##
135.8 HAU1224
0.0 HAU2022
6.4 BNL0827 PGML0844
49.4 NAU5480
55.6 DPL0270
75.6 SWU16437
99.5 SWU16432

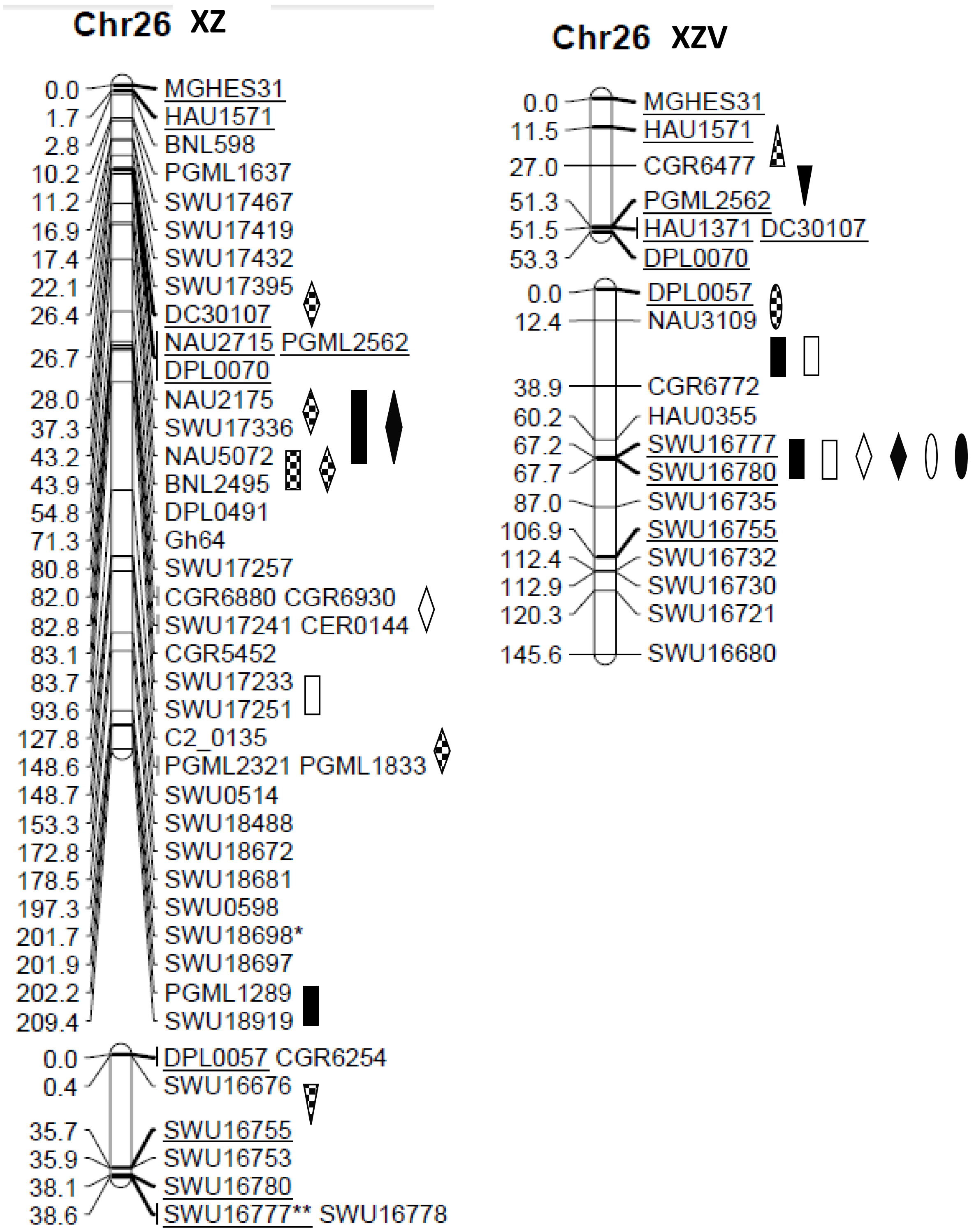
Chr26 XZ
0.0 MGHES31
1.7 HAU1571
2.8 BNL598
10.2 PGML1637
11.2 SWU17467
16.9 SWU17419
17.4 SWU17432
22.1 SWU17395
26.4 DC30107
26.7 NAU2715 PGML2562
DPL0070
28.0 NAU2175
37.3 SWU17336
43.2 NAU5072
43.9 BNL2495
54.8 DPL0491
71.3 Gh64
80.8 SWU17257
82.0 CGR6880 CGR6930
82.8 SWU17241 CER0144
83.1 CGR5452
83.7 SWU17233
93.6 SWU17251
127.8 C2_0135
148.6 PGML2321 PGML1833
148.7 SWU0514
153.3 SWU18488
172.8 SWU18672
178.5 SWU18681
197.3 SWU0598
201.7 SWU18698*
201.9 SWU18697
202.2 PGML1289
209.4 SWU18919
0.0 DPL0057 CGR6254
0.4 SWU16676
35.7 SWU16755
35.9 SWU16753
38.1 SWU16780
38.6 SWU16777** SWU16778
Chr26 XZV
0.0 MGHES31
11.5 HAU1571
27.0 CGR6477
51.3 PGML2562
51.5 HAU1371 DC30107
53.3 DPL0070
0.0 DPL0057
12.4 NAU3109
38.9 CGR6772
60.2 HAU0355
67.2 SWU16777
67.7 SWU16780
87.0 SWU16735
106.9 SWU16755
112.4 SWU16732
112.9 SWU16730
120.3 SWU16721
145.6 SWU16680

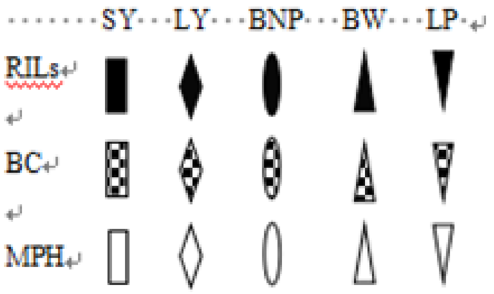

Figure S1 Locations of QTLs controlling yield and yield component traits identified in two hybrids

SY: seed cotton yield; LY: lint yield; BNP: bolls per plant; BW: boll weight; LP: lint percent;

* and **, segregation distortion significant at P = 0.05 and 0.01 levels, respectively.
