# Supplementary material for: Partial Dominance, Overdominance, Epistasis and QTL by Environment Interactions Contribute to Heterosis in Two Upland Cotton Hybrids
Source: G3 (Bethesda). 2015 Dec 29;6(3):499–507. doi: 10.1534/g3.115.025809 (PMC4777113; doi:10.1534/g3.115.025809)
Supplement: Supporting Information [file supp_g3.115.025809_TableS3.doc]

**Table S3 Main effects and environmental interactions detected for yield and yield components in BCF_1_ and BCVF_1_ populations by inclusive composite interval mapping**

| Trait | Chr. | Position | Flanking markers | | LOD | V(A) | V(AE) | A | AE1 | AE2 | AE3 |
| --- | --- | --- | --- | --- | --- | --- | --- | --- | --- | --- | --- |
| BCF_1_ population | | | | | | | | | | | |
| SY | 2 | 44 | SWU11887 | SWU11976 | 3.44 | 1.77 | 1.95 | 1.40 | -1.64 | -0.27 | 1.91 |
|  | 5 | 49 | NAU6240 | PGML1671 | 3.27 | 2.09 | 0.51 | 1.52 | -0.95 | 0.08 | 0.88 |
|  | 20 | 116 | SWU20035 | DPL0319 | 5.00 | 2.24 | 0.23 | -1.57 | -0.19 | -0.49 | 0.68 |
|  | 27 | 51 | ICR01320 | DPL0003 | 4.47 | 2.46 | 3.45 | -1.66 | 1.19 | 1.58 | -2.77 |
|  | 28 | 93 | MGHES24 | ICR11064 | 4.17 | 1.38 | 0.49 | 1.24 | 0.50 | 0.55 | -1.05 |
| LY | 2 | 43 | SWU11887 | SWU11976 | 4.43 | 2.62 | 2.35 | 0.70 | -0.72 | -0.15 | 0.87 |
|  | 3 | 2 | HAU2424 | CER0028 | 2.63 | 1.91 | 0.10 | 0.59 | -0.19 | 0.08 | 0.11 |
|  | 16 | 27 | ICR00647 | SWU20341 | 2.74 | 0.48 | 0.71 | -0.30 | -0.48 | 0.39 | 0.09 |
|  | 20 | 114 | SWU1259 | SWU20033 | 2.52 | 1.02 | 0.39 | -0.43 | -0.04 | -0.31 | 0.35 |
|  | 26 | 149 | SWU0514 | SWU18488 | 3.25 | 1.63 | 0.22 | -0.55 | -0.02 | -0.24 | 0.25 |
|  | 27 | 51 | ICR01320 | DPL0003 | 4.73 | 2.84 | 3.02 | -0.73 | 0.45 | 0.61 | -1.06 |
|  | 28 | 92 | NBRI0014 | SWU12107 | 3.17 | 0.96 | 0.57 | 0.42 | 0.27 | 0.19 | -0.46 |
| BNP | 1 | 159 | CGR5663 | NAU2343 | 3.25 | 0.77 | 1.67 | -0.21 | 0.06 | -0.41 | 0.34 |
|  | 9 | 63 | HAU1618 | NAU2873 | 3.22 | 2.21 | 0.13 | 0.36 | 0.03 | 0.08 | -0.12 |
|  | 11 | 72 | CGR5421 | ICR08245 | 4.35 | 3.16 | 0.51 | -0.43 | 0.24 | -0.06 | -0.17 |
|  | 12 | 59 | Gh631 | HAU1321 | 3.00 | 2.26 | 0.17 | -0.36 | 0.00 | 0.12 | -0.12 |
|  | 14 | 54 | NAU874 | SWU13824 | 2.96 | 2.05 | 0.14 | 0.34 | -0.01 | 0.11 | -0.10 |
|  | 24 | 30 | PGML4657 | Gh454 | 3.59 | 1.86 | 1.50 | 0.33 | -0.08 | -0.31 | 0.40 |
|  | 26 | 156 | SWU18488 | SWU18672 | 3.24 | 2.02 | 0.17 | -0.34 | -0.10 | -0.04 | 0.14 |
|  | 27 | 58 | CGR6356 | SWU11632 | 3.60 | 2.57 | 0.18 | -0.39 | -0.08 | 0.14 | -0.07 |
| BW | 4 | 41 | BNL1167 | SWU21415 | 4.17 | 1.65 | 1.41 | 0.04 | 0.02 | 0.02 | -0.05 |
|  | 4 | 50 | BNL530 | SWU21485 | 4.33 | 2.77 | 0.26 | 0.05 | 0.00 | -0.02 | 0.02 |
|  | 5 | 25 | SWU20917 | NAU6240 | 3.40 | 1.50 | 0.56 | 0.03 | -0.02 | -0.01 | 0.03 |
|  | 5 | 130 | CGR5025 | NBRI0694 | 2.62 | 1.71 | 0.10 | 0.04 | 0.00 | -0.01 | 0.01 |
|  | 6 | 84 | ICR02737 | TMB2940 | 2.81 | 2.05 | 0.09 | -0.04 | 0.01 | -0.01 | 0.00 |
|  | 6 | 100 | HAU1460 | HAU1371 | 3.84 | 2.56 | 0.05 | -0.04 | 0.01 | 0.00 | -0.01 |
|  | 12 | 4 | NAU943 | DPL0303 | 3.47 | 2.39 | 0.04 | -0.04 | 0.00 | 0.00 | -0.01 |
|  | 14 | 71 | SWU14224 | DPL0565 | 4.76 | 2.88 | 1.27 | -0.05 | 0.01 | -0.04 | 0.03 |
|  | 17 | 0 | ICR03391 | SWU12838a | 3.80 | 2.67 | 0.18 | 0.05 | 0.01 | 0.01 | -0.02 |
|  | 20 | 59 | CGR6154 | SWU20246 | 3.64 | 2.44 | 0.24 | -0.04 | 0.00 | -0.01 | 0.02 |
|  | 20 | 83 | CER0167 | SWU20064 | 4.08 | 2.84 | 0.22 | -0.05 | 0.00 | -0.02 | 0.02 |
|  | 21 | 168 | DPL0050a | BNL3171 | 3.10 | 2.25 | 0.04 | -0.04 | 0.00 | 0.00 | 0.01 |
|  | 21 | 187 | CGR5808 | HAU0423 | 3.89 | 2.82 | 0.03 | -0.05 | 0.00 | -0.01 | 0.00 |
|  | 21 | 250 | CGR5806 | DPL0777 | 3.20 | 1.82 | 0.29 | -0.04 | -0.02 | 0.02 | -0.01 |
|  | 23 | 2 | SWU14807 | PGML4185 | 3.06 | 2.16 | 0.01 | 0.04 | 0.00 | 0.00 | 0.00 |
|  | 28 | 94 | ICR11064 | NAU935 | 4.19 | 1.70 | 1.42 | 0.04 | -0.05 | 0.01 | 0.03 |
| LP | 2 | 38 | SWU12025 | SWU11889 | 2.66 | 1.58 | 0.11 | 0.17 | -0.06 | 0.02 | 0.05 |
|  | 3 | 1 | HAU2424 | CER0028 | 3.27 | 2.29 | 0.10 | 0.21 | 0.04 | 0.02 | -0.06 |
|  | 4 | 90 | SWU16783 | NAU3868 | 3.48 | 2.37 | 0.16 | 0.21 | 0.06 | 0.01 | -0.07 |
|  | 5 | 12 | SWU20913 | Gh260 | 10.85 | 6.73 | 0.78 | -0.35 | -0.11 | 0.17 | -0.06 |
|  | 5 | 38 | NAU6240 | PGML1671 | 5.31 | 2.31 | 1.72 | -0.21 | 0.03 | -0.23 | 0.21 |
|  | 6 | 78 | BNL3650 | ICR10602 | 3.17 | 2.28 | 0.09 | -0.21 | -0.01 | -0.05 | 0.05 |
|  | 6 | 86 | CGR5801 | SWU19249 | 3.32 | 2.24 | 0.20 | -0.20 | 0.06 | -0.08 | 0.03 |
|  | 9 | 73 | NAU1282 | CGR6771 | 2.80 | 1.44 | 0.83 | -0.16 | 0.01 | -0.16 | 0.15 |
|  | 13 | 52 | PGML0014 | CGR6732 | 2.56 | 1.79 | 0.19 | -0.19 | 0.00 | -0.07 | 0.07 |
|  | 13 | 79 | BNL1495 | CGR5390 | 4.60 | 2.97 | 0.03 | -0.24 | -0.03 | 0.03 | -0.01 |
|  | 14 | 58 | NAU3308 | HAU1057 | 3.15 | 1.97 | 0.18 | 0.19 | 0.07 | -0.07 | 0.00 |
|  | 14 | 75 | SWU14545 | SWU14543 | 3.08 | 1.45 | 0.46 | 0.16 | 0.07 | -0.13 | 0.06 |
|  | 19 | 81 | NAU3437 | NAU2894 | 6.43 | 4.52 | 0.43 | 0.29 | 0.00 | 0.11 | -0.11 |
|  | 22 | 32 | DPL0562 | CAU0161 | 2.53 | 1.73 | 0.05 | 0.19 | 0.04 | -0.02 | -0.02 |
|  | 24 | 75 | HAU2504 | SWU13736 | 3.35 | 2.35 | 0.14 | 0.21 | 0.05 | 0.02 | -0.07 |
|  | 25 | 117 | SWU19144 | Gh220 | 2.54 | 1.14 | 0.55 | 0.15 | 0.13 | -0.12 | -0.01 |
| BCVF_1_ population | | | | | | | | | | | |
| SY | 14 | 110 | ICR03943 | ICR12281 | 2.56 | 0.05 | 1.34 | 0.26 | -0.56 | 1.85 | -1.29 |
|  | 21 | 58 | SHIN0337 | SWU16370 | 2.86 | 2.03 | 1.07 | 1.60 | -0.77 | -0.88 | 1.65 |
|  | 23 | 200 | NAU2140 | DC40286 | 3.23 | 0.77 | 1.22 | -0.99 | -1.67 | 0.32 | 1.35 |
|  | 23 | 279 | NAU3588 | NAU5373a | 4.34 | 0.77 | 1.99 | -0.99 | -1.61 | -0.55 | 2.17 |
|  | 25 | 62 | DPL0377 | SWU19413 | 2.95 | 0.01 | 2.08 | -0.13 | -0.20 | 2.08 | -1.88 |
| LY | 19 | 8 | NAU2816 | PGML4342 | 2.67 | 1.22 | 1.36 | -0.52 | 0.06 | 0.64 | -0.70 |
|  | 19 | 42 | SWU17789 | SWU17882 | 2.67 | 0.55 | 2.22 | -0.35 | 0.38 | 0.60 | -0.98 |
|  | 23 | 0 | CGR5158 | HAU1758 | 2.76 | 1.47 | 0.80 | -0.59 | 0.59 | -0.15 | -0.44 |
|  | 23 | 90 | BNL3482 | HAU0244 | 2.66 | 1.14 | 1.86 | -0.52 | 0.28 | 0.65 | -0.93 |
|  | 23 | 199 | NAU2140 | DC40286 | 3.92 | 0.97 | 0.98 | -0.46 | -0.62 | 0.11 | 0.51 |
|  | 23 | 279 | NAU3588 | NAU5373a | 4.48 | 0.30 | 2.32 | -0.26 | -0.95 | 0.18 | 0.77 |
|  | 25 | 62 | DPL0377 | SWU19413 | 3.75 | 0.00 | 2.43 | 0.03 | -0.10 | 0.94 | -0.85 |
|  | 35 | 3 | NAU2139 | TMB1152 | 2.59 | 1.59 | 0.74 | -0.60 | 0.43 | 0.12 | -0.55 |
| BNP | 2 | 115 | SWU11976 | SWU12001 | 2.60 | 0.80 | 1.32 | -0.21 | 0.09 | -0.36 | 0.27 |
|  | 23 | 0 | CGR5158 | HAU1758 | 2.98 | 0.00 | 1.92 | 0.01 | 0.44 | -0.37 | -0.07 |
|  | 23 | 278 | NAU2238 | NAU3588 | 4.37 | 0.62 | 2.29 | -0.18 | -0.34 | -0.14 | 0.48 |
| BW | 1 | 38 | SWU17434 | SWU14616 | 2.74 | 1.42 | 0.21 | -0.04 | -0.01 | -0.01 | 0.02 |
|  | 1 | 83 | SWU14514 | Gh120 | 4.31 | 2.40 | 0.30 | -0.05 | -0.01 | -0.01 | 0.02 |
|  | 4 | 51 | BNL530 | SWU16781 | 3.42 | 2.20 | 0.01 | 0.04 | 0.00 | 0.00 | 0.00 |
|  | 7 | 1 | HAU2530 | CGR6586 | 3.93 | 2.15 | 0.30 | -0.04 | -0.02 | -0.01 | 0.02 |
|  | 10 | 11 | NAU3395 | CAU0234 | 3.28 | 0.00 | 2.37 | 0.00 | -0.05 | -0.01 | 0.06 |
|  | 14 | 83 | ICR01124 | HAU2482 | 2.59 | 1.58 | 0.37 | -0.05 | -0.01 | 0.03 | -0.02 |
|  | 23 | 0 | CGR5158 | HAU1758 | 2.66 | 1.96 | 0.09 | -0.04 | -0.01 | 0.01 | 0.00 |
|  | 23 | 49 | HAU1758 | SHIN1076 | 2.84 | 0.05 | 2.37 | -0.01 | 0.05 | 0.01 | -0.06 |
|  | 25 | 0 | HAU3012 | SWU19676 | 3.62 | 2.24 | 0.08 | 0.04 | 0.00 | 0.01 | -0.01 |
|  | 26 | 27 | CGR6477 | PGML2562 | 5.14 | 2.96 | 1.17 | -0.05 | -0.04 | 0.03 | 0.01 |
|  | 32 | 2 | TMB0071 | HAU1000 | 4.95 | 3.72 | 0.31 | -0.06 | -0.01 | 0.02 | -0.01 |
|  | 36 | 9 | SWU20700 | CER0167 | 3.41 | 2.61 | 0.44 | -0.05 | 0.00 | 0.02 | -0.03 |
| LP | 4 | 64 | SWU16783 | SWU18876 | 3.42 | 1.96 | 0.03 | -0.18 | 0.03 | -0.03 | 0.00 |
|  | 6 | 58 | BNL3650 | TMB2940 | 3.24 | 2.15 | 0.44 | -0.19 | -0.12 | 0.02 | 0.09 |
|  | 6 | 86 | SWU19656 | CGR5124 | 2.51 | 1.84 | 0.05 | -0.18 | -0.02 | -0.02 | 0.04 |
|  | 13 | 31 | SWU13032 | HAU2850 | 4.79 | 3.02 | 0.07 | -0.23 | -0.01 | 0.04 | -0.04 |
|  | 16 | 39 | NAU747 | HAU1129 | 2.88 | 1.98 | 0.38 | -0.18 | -0.11 | 0.06 | 0.05 |
|  | 23 | 277 | NAU2238 | NAU3588 | 2.72 | 1.13 | 1.05 | -0.14 | 0.06 | -0.19 | 0.13 |
|  | 23 | 282 | NAU5373b | HAU2648 | 4.56 | 1.70 | 2.91 | -0.17 | -0.32 | 0.15 | 0.17 |
|  | 38 | 10 | NAU2450 | PGML1942 | 4.43 | 2.36 | 0.22 | -0.20 | 0.07 | -0.08 | 0.01 |

See footnotes of additional table S2 for explanations
