# Supplementary material for: Partial Dominance, Overdominance, Epistasis and QTL by Environment Interactions Contribute to Heterosis in Two Upland Cotton Hybrids
Source: G3 (Bethesda). 2015 Dec 29;6(3):499–507. doi: 10.1534/g3.115.025809 (PMC4777113; doi:10.1534/g3.115.025809)
Supplement: Supporting Information [file supp_g3.115.025809_TableS4.doc]

**Table S4 Main effects and environmental interactions detected for yield and yield components in two MPH data by inclusive composite interval mapping**

| Trait | Chr. | Position | Flanking markers | | LOD | V(A) | V(AE) | A | AE1 | AE2 | AE3 |
| --- | --- | --- | --- | --- | --- | --- | --- | --- | --- | --- | --- |
| MPH (XZ hybrid) | | | | | | | | | | | |
| SY | 14 | 54 | NAU874 | SWU13824 | 2.53 | 1.61 | 0.13 | 1.18 | 0.15 | 0.32 | -0.47 |
|  | 16 | 75 | SWU10266 | DC40065 | 3.40 | 0.02 | 2.92 | 0.13 | 0.10 | 1.89 | -1.99 |
|  | 27 | 58 | CGR6356 | SWU11632 | 2.72 | 1.53 | 0.59 | -1.15 | -0.40 | 1.01 | -0.61 |
| LY | 16 | 74 | SWU10266 | DC40065 | 3.80 | 0.00 | 3.28 | 0.00 | 0.07 | 0.79 | -0.86 |
|  | 27 | 51 | ICR01320 | DPL0003 | 2.79 | 0.88 | 2.03 | -0.35 | 0.05 | 0.63 | -0.68 |
|  | 27 | 58 | CGR6356 | SWU11632 | 2.78 | 1.56 | 0.54 | -0.47 | -0.20 | 0.39 | -0.19 |
| BNP | 9 | 63 | HAU1618 | NAU2873 | 3.47 | 1.37 | 1.10 | 0.27 | 0.28 | 0.04 | -0.31 |
|  | 11 | 73 | CGR5421 | ICR08245 | 3.51 | 2.41 | 0.50 | -0.36 | 0.23 | -0.08 | -0.15 |
|  | 21 | 52 | SWU16487 | SWU16488 | 2.73 | 1.33 | 0.57 | -0.27 | -0.14 | -0.10 | 0.25 |
|  | 27 | 57 | CGR6356 | SWU11632 | 2.52 | 1.64 | 0.32 | -0.30 | -0.09 | 0.18 | -0.09 |
| BW | 5 | 124 | PGML4350 | SWU17781 | 2.86 | 0.00 | 2.45 | 0.00 | -0.05 | 0.00 | 0.05 |
|  | 5 | 152 | SWU13378 | SWU17846 | 2.66 | 0.53 | 1.48 | -0.02 | -0.02 | -0.03 | 0.05 |
|  | 6 | 41 | ICR00143 | CGR5108 | 2.63 | 0.19 | 1.87 | -0.01 | -0.02 | -0.03 | 0.05 |
|  | 20 | 0 | SWU20700 | CGR5548 | 4.38 | 1.68 | 1.66 | -0.04 | -0.01 | -0.04 | 0.05 |
|  | 25 | 29 | BNL3594 | DPL0282 | 2.72 | 1.67 | 0.41 | -0.03 | 0.02 | -0.01 | -0.01 |
| LP | 5 | 32 | NAU6240 | PGML1671 | 3.06 | 0.02 | 2.20 | 0.01 | -0.11 | -0.10 | 0.21 |
|  | 12 | 1 | NAU943 | DPL0303 | 2.60 | 2.01 | 0.03 | -0.14 | -0.01 | -0.02 | 0.02 |
|  | 13 | 54 | PGML0014 | CGR6732 | 2.87 | 0.06 | 1.92 | -0.03 | -0.07 | -0.13 | 0.20 |
|  | 13 | 72 | Gh157 | BNL1495 | 2.85 | 0.82 | 1.43 | -0.09 | -0.10 | -0.08 | 0.17 |
|  | 20 | 138 | DPL0319 | HAU1378 | 3.44 | 2.67 | 0.05 | 0.17 | 0.03 | 0.00 | -0.03 |
| MPH (XZV hybrid) | | | | | | | | | | | |
| SY | 2 | 3 | DPL0217 | CGR6695 | 2.60 | 0.51 | 2.23 | -1.04 | 2.46 | 0.36 | -2.82 |
|  | 16 | 0 | HAU3081 | NAU747 | 2.66 | 0.19 | 1.03 | 0.63 | 2.09 | -1.11 | -0.97 |
|  | 21 | 231 | BNL3171 | HAU2937 | 2.57 | 1.63 | 0.72 | 1.87 | -1.46 | -0.12 | 1.58 |
|  | 26 | 52 | DC30107 | DPL0070 | 3.12 | 0.27 | 2.16 | -0.75 | 2.80 | -0.42 | -2.37 |
|  | 31 | 68 | SWU16780 | SWU16735 | 3.21 | 0.28 | 2.93 | -0.76 | 2.86 | 0.35 | -3.20 |
| LY | 19 | 46 | DC40130 | SWU17897 | 2.83 | 1.74 | 2.92 | -0.78 | 0.77 | 0.66 | -1.43 |
|  | 21 | 231 | BNL3171 | HAU2937 | 2.97 | 1.97 | 0.93 | 0.84 | -0.61 | -0.16 | 0.77 |
|  | 31 | 68 | SWU16780 | SWU16735 | 2.98 | 0.14 | 2.44 | -0.22 | 1.09 | 0.08 | -1.17 |
| BNP | 2 | 16 | SWU11013 | DPL0041 | 2.80 | 2.07 | 0.94 | -0.42 | 0.33 | 0.04 | -0.36 |
|  | 3 | 14 | SWU12840 | NAU2742 | 3.18 | 2.03 | 0.07 | -0.42 | 0.10 | -0.09 | 0.00 |
|  | 21 | 231 | BNL3171 | HAU2937 | 4.05 | 3.06 | 0.70 | 0.51 | -0.34 | 0.09 | 0.25 |
|  | 26 | 53 | DC30107 | DPL0070 | 4.14 | 3.22 | 0.38 | -0.52 | 0.20 | 0.04 | -0.24 |
|  | 31 | 67 | HAU0355 | SWU16777 | 3.11 | 2.45 | 1.22 | -0.46 | 0.26 | 0.19 | -0.45 |
|  | 31 | 117 | SWU16730 | SWU16721 | 3.24 | 2.24 | 0.18 | 0.44 | -0.17 | 0.05 | 0.12 |
| BW | 7 | 5 | CGR5372 | C2_0046 | 3.09 | 1.13 | 0.57 | -0.03 | -0.02 | -0.01 | 0.03 |
|  | 12 | 54 | ICR03107 | HAU3373 | 2.52 | 0.67 | 1.50 | 0.03 | -0.05 | 0.01 | 0.04 |
|  | 17 | 28 | HAU1413 | CGR5576 | 2.56 | 1.22 | 0.66 | -0.04 | -0.02 | 0.04 | -0.02 |
|  | 19 | 50 | SWU17897 | TMB0107 | 4.33 | 2.85 | 0.00 | -0.05 | 0.00 | 0.00 | 0.00 |
|  | 23 | 54 | HAU1758 | SHIN1076 | 3.53 | 0.03 | 2.44 | 0.01 | 0.05 | 0.02 | -0.07 |
| LP | 19 | 47 | DC40130 | SWU17897 | 2.58 | 1.37 | 0.45 | -0.14 | -0.05 | 0.11 | -0.06 |

See footnotes of additional table S2 for explanations
