# Supplementary material for: Partial Dominance, Overdominance, Epistasis and QTL by Environment Interactions Contribute to Heterosis in Two Upland Cotton Hybrids
Source: G3 (Bethesda). 2015 Dec 29;6(3):499–507. doi: 10.1534/g3.115.025809 (PMC4777113; doi:10.1534/g3.115.025809)
Supplement: Supporting Information [file supp_g3.115.025809_TableS5.doc]

Table S5 Epistatic effects and environmental interactions detected for yield and yield components in RIL and RILV populations using two-locus analysis by inclusive composite interval mapping

| Trait | Chi | Flanking markers | | Chj | Flanking markers | | LOD | V(AA) | V(AAE) | AA | AAE1 | AAE2 | AAE3 |
| --- | --- | --- | --- | --- | --- | --- | --- | --- | --- | --- | --- | --- | --- |
| RIL population | | | | | | | | | | | | | |
| SY | 1 | **SWU10986** | **NAU2218** | 2 | SWU11887 | SWU11976 | 6.01 | 3.20 | 0.28 | -2.48 | 0.48 | 0.55 | -1.03 |
|  | 9 | NAU1282 | CGR6771 | 10 | SWU20501b | CGR5873 | 5.16 | 3.03 | 0.05 | 2.43 | -0.45 | 0.24 | 0.21 |
|  | 5 | NAU6240 | PGML1671 | 10 | NAU2139 | SWU20689 | 5.22 | 2.55 | 0.27 | -2.23 | -0.06 | 0.90 | -0.84 |
|  | 7 | NAU3181 | SHIN0376 | 15 | DC40183 | DC40175 | 6.36 | 3.65 | 0.06 | 2.64 | -0.42 | -0.27 | 0.69 |
|  | 14 | SWU14224 | DPL0565 | 15 | DPL0182 | SWU11691 | 6.45 | 3.55 | 0.19 | 2.62 | -0.22 | -0.59 | 0.81 |
|  | 10 | CGR5873 | ICR00093 | 16 | Gh56 | NAU5120 | 5.74 | 3.40 | 0.00 | 2.59 | 0.22 | 0.43 | -0.65 |
|  | 3 | CER0028 | Gh663 | 20 | SWU20246 | SWU20501a | 5.72 | 3.37 | 0.07 | 2.59 | -0.45 | 0.28 | 0.17 |
|  | 22 | DPL0562 | CAU0161 | 24 | CGR5202 | Gh298 | 5.16 | 2.69 | 0.14 | -2.47 | -0.85 | 0.24 | 0.61 |
|  | 6 | ICR00143 | CGR5108 | 24 | SWU13758 | CGR5423 | 5.01 | 2.85 | 0.11 | -2.33 | 0.63 | -0.21 | -0.42 |
|  | 21 | SWU16651 | SWU16645 | 25 | SWU19848 | CGR6864 | 5.29 | 2.79 | 0.36 | 2.34 | -1.11 | 0.30 | 0.81 |
|  | 2 | SWU11950 | TMB1268 | 26 | BNL2495 | DPL0491 | 6.30 | 3.10 | 0.60 | -2.44 | 1.22 | 0.17 | -1.39 |
|  | 6 | SWU19541 | CGR5801 | 27 | SWU11384 | ICR11885 | 6.60 | 2.59 | 1.42 | 2.23 | -2.04 | 0.13 | 1.92 |
| LY | 1 | **SWU10986** | **NAU2218** | 2 | SWU11887 | SWU11976 | 6.72 | 3.34 | 0.43 | -1.04 | 0.17 | 0.35 | -0.52 |
|  | 1 | BNL2827a | NAU6367 | 3 | SWU12819 | SWU12765 | 5.56 | 3.23 | 0.32 | 1.02 | -0.01 | -0.38 | 0.40 |
|  | 1 | HAU1417 | NAU2437 | 13 | NAU3468 | SWU22309 | 5.53 | 3.30 | 0.08 | -1.05 | 0.07 | 0.08 | -0.15 |
|  | 14 | SWU14224 | DPL0565 | 15 | DPL0182 | SWU11691 | 6.36 | 3.50 | 0.27 | 1.06 | 0.01 | -0.37 | 0.36 |
|  | 16 | SWU10062 | SWU10094 | 20 | SWU20246 | SWU20501a | 5.26 | 2.83 | 0.38 | 0.96 | -0.30 | -0.19 | 0.49 |
|  | 7 | SWU10205 | HAU1483a | 23 | PGML4186 | NAU3100 | 5.75 | 3.25 | 0.30 | -1.03 | 0.08 | 0.33 | -0.41 |
|  | 20 | SWU20035 | DPL0319 | 25 | SWU19430 | PGML1219 | 5.52 | 3.01 | 0.37 | 0.98 | -0.10 | -0.36 | 0.46 |
|  | 20 | SWU20636 | CGR6154 | 28 | SWU12343 | SWU14060 | 5.05 | 2.65 | 0.43 | 0.93 | 0.42 | -0.49 | 0.06 |
|  | 7 | Gh474 | SWU10785 | 28 | NBRI0014 | SWU12107 | 5.39 | 2.79 | 0.29 | -0.94 | -0.25 | 0.44 | -0.20 |
|  | 10 | ICR00093 | ICR07050 | 30 | BNL243 | CER0168 | 5.52 | 2.63 | 0.75 | -0.92 | -0.12 | 0.66 | -0.54 |
| BNP | 1 | ICR03724 | ICR03725 | 2 | PGML0700 | SWU12016 | 5.16 | 3.27 | 0.01 | -0.56 | 0.02 | 0.04 | -0.06 |
|  | 1 | NAU3384 | CGR5663 | 8 | DC20094 | HAU1470b | 5.02 | 2.59 | 0.52 | -0.50 | -0.26 | 0.29 | -0.03 |
|  | 1 | HAU1417 | NAU2437 | 9 | Gh27 | SWU15194 | 5.43 | 3.08 | 0.36 | 0.54 | 0.14 | -0.26 | 0.13 |
|  | 7 | SWU10067 | SWU10064 | 14 | SWU13909 | TMB0071 | 5.40 | 3.86 | 0.19 | 0.61 | 0.06 | -0.19 | 0.13 |
|  | 7 | SWU10064 | NAU3181 | 16 | SWU10038 | ICR00016 | 5.72 | 2.92 | 0.57 | -0.61 | 0.10 | 0.26 | -0.36 |
|  | 1 | SWU0077 | HAU1417 | 19 | NAU5330 | Gh72 | 5.34 | 2.70 | 0.88 | 0.52 | 0.00 | -0.36 | 0.36 |
|  | 10 | SWU20501b | CGR5873 | 20 | SWU20700 | CGR5548 | 5.42 | 2.43 | 1.28 | -0.49 | -0.12 | 0.49 | -0.37 |
|  | 2 | PGML0700 | SWU12016 | 21 | Gh451 | SWU16489 | 7.99 | 4.99 | 0.10 | -0.70 | 0.09 | 0.04 | -0.13 |
|  | 1 | NAU3384 | CGR5663 | 21 | SWU14431a | SWU15915 | 5.02 | 3.23 | 0.34 | -0.57 | 0.19 | 0.06 | -0.25 |
|  | 16 | Gh56 | NAU5120 | 21 | BNL3171 | CGR5808 | 5.39 | 2.94 | 0.55 | 0.55 | 0.34 | -0.22 | -0.12 |
|  | 11 | NAU3390 | NAU2460 | 25 | HAU1382 | SWU19848 | 5.83 | 3.99 | 0.26 | -0.62 | 0.13 | 0.09 | -0.22 |
|  | 10 | ICR00093 | ICR07050 | 26 | MGHES31 | HAU1571 | 5.59 | 2.78 | 1.03 | -0.52 | 0.05 | 0.36 | -0.41 |
|  | 22 | SWU21533 | DPL0562 | 30 | BNL243 | CER0168 | 7.74 | 1.70 | 3.09 | -0.42 | -0.12 | 0.74 | -0.62 |
|  | 28 | SWU12343 | SWU14060 | 30 | BNL243 | CER0168 | 5.45 | 3.99 | 0.07 | 0.63 | 0.12 | -0.07 | -0.05 |
|  | 16 | Gh56 | NAU5120 | 30 | BNL243 | CER0168 | 5.37 | 3.21 | 0.42 | 0.56 | 0.27 | -0.19 | -0.07 |
| BW | 1 | DPL0090 | Gh398 | 8 | NAU4064 | CGR6508 | 5.73 | 3.55 | 0.02 | 0.09 | 0.01 | -0.01 | 0.00 |
|  | 7 | SWU10205 | HAU1483a | 10 | BNL2960 | SWU20511 | 5.74 | 2.14 | 1.02 | -0.07 | -0.06 | 0.04 | 0.02 |
|  | 1 | NAU3384 | CGR5663 | 11 | NAU1014 | ICR10344 | 6.62 | 4.01 | 0.19 | -0.09 | -0.02 | -0.01 | 0.02 |
|  | 8 | DC20094 | HAU1470b | 12 | **NAU943** | **DPL0303** | 9.27 | 5.40 | 0.08 | -0.10 | -0.02 | 0.01 | 0.01 |
|  | 5 | **PGML1917** | **SWU17715** | 13 | SWU22413 | CGR5331 | 7.04 | 4.21 | 0.13 | 0.09 | 0.01 | 0.01 | -0.02 |
|  | 6 | **ICR03206** | **NAU896** | 13 | SWU13032 | DPL0308 | 5.19 | 2.83 | 0.35 | -0.08 | -0.03 | 0.00 | 0.03 |
|  | 1 | NAU3384 | CGR5663 | 14 | **SWU14224** | **DPL0565** | 5.40 | 2.64 | 0.42 | 0.07 | 0.04 | 0.00 | -0.04 |
|  | 13 | DPL0535 | CER0165 | 16 | HAU1129 | NAU2984 | 6.16 | 3.34 | 0.40 | -0.08 | -0.04 | 0.02 | 0.02 |
|  | 16 | HAU1129 | NAU2984 | 18 | DC40150 | ICR02849 | 5.91 | 3.09 | 0.45 | -0.08 | -0.04 | 0.03 | 0.02 |
|  | 16 | PGML1709 | SWU10627 | 19 | NAU1042 | NAU3437 | 5.66 | 3.30 | 0.14 | -0.08 | -0.02 | 0.00 | 0.02 |
|  | 2 | SWU12025 | SWU11889 | 20 | SWU20700 | CGR5548 | 5.87 | 3.58 | 0.17 | 0.09 | 0.03 | -0.01 | -0.02 |
|  | 14 | SWU14545 | SWU14543 | 21 | SWU16487 | SWU16488 | 6.68 | 3.83 | 0.12 | -0.09 | 0.00 | 0.02 | -0.02 |
|  | 9 | SWU15157 | SWU14934 | 21 | SWU16138 | BNL1053 | 5.55 | 2.74 | 0.36 | 0.08 | 0.03 | 0.00 | -0.03 |
|  | 16 | PGML1709 | SWU10627 | 21 | SWU16138 | BNL1053 | 7.14 | 4.03 | 0.15 | 0.09 | 0.03 | -0.01 | -0.02 |
|  | 11 | CGR5421 | ICR08245 | 23 | PGML4186 | NAU3100 | 6.13 | 3.83 | 0.02 | 0.09 | 0.01 | 0.00 | -0.02 |
|  | 9 | Gh158 | DC40407 | 24 | CGR5202 | Gh298 | 5.27 | 3.19 | 0.10 | 0.08 | -0.01 | -0.01 | 0.02 |
|  | 21 | SWU16649 | BNL1552 | 24 | HAU2504 | SWU13736 | 6.41 | 3.81 | 0.02 | -0.09 | 0.01 | 0.00 | -0.01 |
|  | 1 | SWU0077 | HAU1417 | 25 | CGR6864 | SWU19815 | 7.64 | 4.40 | 0.33 | -0.10 | -0.04 | 0.02 | 0.02 |
|  | 16 | SWU10266 | DC40065 | 25 | BNL3594 | DPL0282 | 5.32 | 2.83 | 0.19 | 0.08 | 0.01 | -0.03 | 0.02 |
|  | 4 | SWU18881 | NAU2701 | 25 | SWU19129 | PGML2858 | 5.33 | 3.23 | 0.13 | -0.08 | -0.02 | 0.00 | 0.02 |
|  | 8 | CGR6508 | Gh197 | 25 | SWU19144 | Gh220 | 6.09 | 3.34 | 0.39 | 0.08 | 0.04 | -0.02 | -0.02 |
|  | 24 | SWU13758 | CGR5423 | 26 | BNL598 | PGML1637 | 5.54 | 2.40 | 0.81 | 0.07 | 0.05 | -0.05 | 0.00 |
|  | 1 | SWU10912 | DPL0090 | 26 | DPL0491 | Gh64 | 5.79 | 2.86 | 0.49 | 0.08 | 0.04 | -0.03 | -0.01 |
|  | 18 | SWU22187 | DC40150 | 26 | C2_0135 | PGML2321 | 8.21 | 4.74 | 0.07 | 0.10 | 0.01 | -0.02 | 0.01 |
|  | 6 | CGR5108 | ICR03206 | 27 | SWU11038 | SWU11384 | 5.36 | 3.23 | 0.17 | 0.08 | 0.03 | -0.01 | -0.01 |
|  | 8 | HAU3177 | NAU4064 | 28 | HAU3071 | CGR5534 | 5.19 | 3.26 | 0.21 | -0.08 | -0.01 | -0.02 | 0.03 |
|  | 5 | DPL0022 | SWU17787 | 28 | CGR5534 | SHIN0219 | 6.79 | 4.10 | 0.05 | 0.09 | -0.01 | 0.01 | 0.00 |
|  | 14 | PGML1568 | Gh529 | 28 | SHIN0219 | TMB2386 | 8.30 | 4.45 | 0.45 | 0.10 | 0.04 | -0.02 | -0.02 |
|  | 17 | CGR5576 | NAU3765 | 28 | SHIN0219 | TMB2386 | 6.70 | 4.10 | 0.03 | 0.09 | 0.01 | 0.01 | -0.01 |
|  | 20 | SWU20636 | CGR6154 | 28 | SHIN0219 | TMB2386 | 6.54 | 4.24 | 0.01 | 0.09 | 0.00 | 0.00 | 0.00 |
|  | 15 | CGR6889 | DPL0182 | 28 | NBRI0014 | SWU12107 | 6.02 | 3.70 | 0.14 | -0.09 | -0.02 | 0.02 | 0.01 |
|  | 24 | SWU13267 | BNL1521 | 29 | DC20127 | DPL0252 | 5.17 | 3.17 | 0.08 | -0.08 | -0.01 | 0.02 | -0.01 |
|  | 2 | PGML0700 | SWU12016 | 29 | DC20127 | DPL0252 | 6.24 | 3.17 | 0.51 | -0.08 | -0.03 | 0.05 | -0.02 |
|  | 13 | DPL0572 | HAU2558 | 32 | NAU2140 | NAU2957 | 7.75 | 4.41 | 0.18 | 0.10 | 0.03 | -0.02 | 0.00 |
|  | 18 | DC40150 | ICR02849 | 32 | NAU2140 | NAU2957 | 5.07 | 3.10 | 0.08 | 0.08 | 0.01 | -0.02 | 0.00 |
|  | 29 | C2_0115 | ICR03107 | 32 | NAU2140 | NAU2957 | 5.85 | 3.22 | 0.34 | 0.08 | 0.03 | -0.03 | 0.00 |
| LP | 1 | SWU10912 | DPL0090 | 3 | CER0028 | Gh663 | 5.89 | 3.69 | 0.10 | 0.43 | -0.08 | 0.03 | 0.05 |
|  | 2 | SWU12126 | SWU12147 | 7 | Gh474 | SWU10785 | 6.68 | 4.00 | 0.22 | -0.44 | -0.13 | 0.12 | 0.00 |
|  | 4 | SWU18881 | NAU2701 | 7 | Gh474 | SWU10785 | 6.20 | 3.72 | 0.30 | -0.43 | -0.17 | 0.12 | 0.05 |
|  | 4 | SWU12672 | HAU1332 | 8 | CGR6508 | Gh197 | 5.19 | 3.52 | 0.05 | -0.42 | -0.03 | 0.07 | -0.05 |
|  | 5 | SWU17787 | SWU13378 | 8 | DC20094 | HAU1470b | 5.93 | 3.89 | 0.00 | 0.44 | 0.00 | 0.00 | 0.00 |
|  | 9 | Gh27 | SWU15194 | 11 | NAU3390 | NAU2460 | 5.12 | 3.08 | 0.10 | -0.39 | -0.10 | 0.05 | 0.05 |
|  | 4 | **SWU16783** | **NAU3868** | 11 | CER0098 | CGR5421 | 5.63 | 3.57 | 0.19 | -0.42 | -0.12 | 0.06 | 0.05 |
|  | 11 | NAU5428 | Gh256 | 12 | HAU1316 | NAU3519 | 5.70 | 3.72 | 0.01 | -0.43 | -0.04 | 0.02 | 0.02 |
|  | 7 | SWU10067 | SWU10064 | 12 | HAU1316 | NAU3519 | 5.47 | 3.45 | 0.02 | -0.42 | -0.01 | 0.04 | -0.03 |
|  | 1 | **NAU3384** | **CGR5663** | 13 | SWU22413 | CGR5331 | 6.58 | 4.01 | 0.03 | -0.44 | 0.02 | 0.03 | -0.05 |
|  | 6 | ICR10602 | SWU19656 | 13 | BNL1495 | CGR5390 | 6.14 | 3.54 | 0.06 | 0.43 | 0.01 | -0.08 | 0.07 |
|  | 4 | SWU12672 | HAU1332 | 13 | **DPL0894** | **SWU10800** | 5.96 | 3.93 | 0.04 | 0.45 | 0.02 | -0.06 | 0.04 |
|  | 5 | PGML4350 | SWU17781 | 14 | PGML4763 | SWU13909 | 7.29 | 4.53 | 0.05 | 0.47 | 0.05 | 0.01 | -0.06 |
|  | 7 | SWU10785 | CER0036 | 14 | ICR12037 | CGR5675 | 6.07 | 4.01 | 0.00 | 0.45 | 0.01 | 0.00 | -0.01 |
|  | 16 | PGML1709 | SWU10627 | 16 | SWU20341 | DPL0897 | 7.07 | 3.82 | 0.39 | -0.48 | -0.10 | 0.22 | -0.12 |
|  | 6 | ICR10602 | SWU19656 | 16 | DPL0048 | SWU10266 | 5.67 | 3.49 | 0.10 | 0.42 | 0.06 | -0.10 | 0.04 |
|  | 11 | NAU3695 | DPL0050b | 18 | NAU748 | SWU22192 | 5.82 | 3.54 | 0.03 | 0.42 | -0.02 | -0.01 | 0.03 |
|  | 14 | SWU14224 | DPL0565 | 19 | DC40122 | NAU833a | 6.26 | 3.58 | 0.07 | -0.44 | -0.07 | 0.08 | 0.00 |
|  | 16 | ICR00010 | SWU10038 | 19 | NAU1042 | NAU3437 | 5.17 | 3.12 | 0.12 | -0.40 | 0.03 | 0.07 | -0.11 |
|  | 1 | NAU6367 | MUSS422 | 20 | CGR5548 | SWU20675 | 6.20 | 3.89 | 0.05 | -0.46 | 0.01 | 0.05 | -0.06 |
|  | 7 | CER0036 | PGML1916 | 20 | SWU20246 | SWU20501a | 6.36 | 3.72 | 0.32 | 0.44 | 0.12 | 0.02 | -0.14 |
|  | 14 | NAU3820 | NAU2960 | 20 | SWU20035 | DPL0319 | 5.12 | 3.11 | 0.21 | -0.39 | -0.11 | 0.14 | -0.03 |
|  | 15 | DC40183 | DC40175 | 21 | SWU16651 | SWU16645 | 8.78 | 5.25 | 0.09 | 0.51 | 0.04 | -0.09 | 0.05 |
|  | 2 | PGML0700 | SWU12016 | 21 | Gh451 | SWU16489 | 5.93 | 3.43 | 0.49 | -0.41 | 0.17 | 0.03 | -0.21 |
|  | 12 | DPL0303 | COT107 | 21 | Gh451 | SWU16489 | 6.96 | 4.25 | 0.17 | 0.46 | 0.13 | -0.05 | -0.08 |
|  | 21 | Gh451 | SWU16489 | 21 | SWU16488 | SWU16138 | 8.02 | 5.27 | 0.01 | -0.53 | 0.02 | 0.03 | -0.05 |
|  | 16 | SWU10627 | PGML1309 | 21 | SWU15915 | SWU0189 | 5.51 | 3.54 | 0.02 | -0.42 | -0.01 | -0.02 | 0.04 |
|  | 19 | SWU17782 | DPL0056 | 21 | CGR5806 | DPL0777 | 5.57 | 3.03 | 0.22 | -0.39 | -0.14 | 0.12 | 0.01 |
|  | 13 | SWU22374 | HAU2857 | 22 | SWU21646 | SWU21585 | 5.83 | 3.25 | 0.24 | 0.41 | 0.10 | -0.16 | 0.06 |
|  | 22 | PGML1712 | SWU21538 | 22 | CAU0161 | NAU2026 | 5.15 | 2.64 | 0.39 | 0.40 | 0.00 | -0.18 | 0.18 |
|  | 19 | NAU3437 | NAU2894 | 23 | SWU14807 | PGML4185 | 5.09 | 3.16 | 0.10 | 0.40 | 0.09 | -0.09 | 0.01 |
|  | 6 | NAU896 | BNL3650 | 23 | PGML4186 | NAU3100 | 5.47 | 3.12 | 0.26 | -0.39 | 0.16 | -0.07 | -0.09 |
|  | 5 | SWU20917 | NAU6240 | 25 | HAU1382 | SWU19848 | 7.14 | 4.47 | 0.02 | 0.47 | 0.01 | 0.00 | -0.02 |
|  | 1 | SWU0077 | HAU1417 | 25 | SWU19411 | SWU19412 | 5.49 | 3.13 | 0.32 | -0.40 | -0.06 | 0.18 | -0.11 |
|  | 21 | Gh451 | SWU16489 | 26 | NAU2175 | SWU17336 | 5.33 | 3.48 | 0.05 | 0.42 | 0.02 | -0.06 | 0.05 |
|  | 22 | SWU21533 | DPL0562 | 26 | Gh64 | SWU17257 | 5.65 | 3.26 | 0.06 | 0.41 | -0.06 | 0.01 | 0.05 |
|  | 15 | **DC40183** | **DC40175** | 26 | C2_0135 | PGML2321 | 5.29 | 3.14 | 0.27 | 0.39 | 0.15 | -0.03 | -0.12 |
|  | 13 | SWU22413 | CGR5331 | 26 | SWU0514 | SWU18488 | 6.08 | 3.90 | 0.03 | -0.44 | 0.02 | 0.03 | -0.05 |
|  | 14 | SWU14224 | DPL0565 | 26 | SWU18488 | SWU18672 | 5.52 | 3.33 | 0.02 | -0.41 | -0.03 | 0.03 | 0.00 |
|  | 20 | SWU20675 | SWU20649 | 26 | SWU18488 | SWU18672 | 5.44 | 3.33 | 0.10 | -0.43 | -0.04 | -0.05 | 0.10 |
|  | 16 | PGML1709 | SWU10627 | 26 | SWU18488 | SWU18672 | 5.79 | 3.88 | 0.05 | 0.44 | 0.06 | -0.02 | -0.04 |
|  | 26 | SWU17251 | C2_0135 | 26 | SWU18488 | SWU18672 | 5.69 | 3.46 | 0.01 | -0.44 | -0.03 | -0.04 | 0.07 |
|  | 16 | CGR6802 | HAU1129 | 27 | SWU11038 | SWU11384 | 7.96 | 5.04 | 0.00 | 0.50 | -0.04 | 0.05 | -0.01 |
|  | 3 | SWU12765 | NAU3839 | 27 | CGR6857 | ICR11205 | 5.08 | 3.11 | 0.07 | 0.40 | 0.09 | -0.01 | -0.07 |
|  | 26 | C2_0135 | PGML2321 | 28 | BNL2877 | HAU3071 | 6.00 | 3.80 | 0.04 | -0.43 | -0.04 | -0.03 | 0.06 |
|  | 12 | DPL0303 | COT107 | 28 | CGR5534 | SHIN0219 | 5.47 | 3.54 | 0.00 | -0.42 | 0.02 | 0.01 | -0.02 |
|  | 11 | NAU5428 | Gh256 | 28 | SWU12343 | SWU14060 | 5.01 | 3.10 | 0.08 | -0.40 | -0.08 | 0.07 | 0.01 |
|  | 16 | SWU10062 | SWU10094 | 29 | C2_0115 | ICR03107 | 6.21 | 4.09 | 0.08 | 0.46 | 0.04 | -0.09 | 0.05 |
| RILV population | | | | | | | | | | | | | |
| SY | 4 | BNL1167 | JESPR234 | 4 | SWU16783 | SWU18876 | 5.92 | 1.55 | 1.68 | -2.91 | 3.30 | 0.77 | -4.07 |
|  | 2 | TMB1268 | SWU11976 | 6 | SWU19656 | CGR5124 | 6.76 | 2.40 | 1.86 | 3.54 | -3.09 | -1.18 | 4.27 |
|  | 4 | JESPR234 | BNL530 | 6 | SWU19184 | DPL0847 | 5.59 | 1.27 | 1.89 | 2.58 | -3.53 | -0.58 | 4.11 |
|  | 4 | BNL530 | SWU16781 | 7 | HAU2530 | CGR6586 | 5.06 | 1.78 | 1.38 | -3.06 | 2.97 | 0.58 | -3.55 |
|  | 1 | PGML2498 | SWU14490 | 12 | HAU3373 | CGR6847 | 5.75 | 2.87 | 0.72 | -3.88 | 2.23 | 0.26 | -2.49 |
|  | 10 | SWU13030 | NAU4967 | 12 | DPL0400 | HAU2173 | 5.52 | 1.40 | 1.34 | 2.74 | -3.22 | -0.04 | 3.26 |
|  | 8 | HAU0810 | TMB2904 | 12 | DPL0400 | HAU2173 | 5.51 | 2.37 | 0.93 | -3.52 | 2.51 | 0.28 | -2.79 |
|  | 12 | DPL0400 | HAU2173 | 13 | SWU13032 | HAU2850 | 5.07 | 1.35 | 1.28 | 2.67 | -2.93 | -0.45 | 3.38 |
|  | 3 | SWU12840 | NAU2742 | 13 | NAU3398 | CGR5331 | 5.26 | 1.56 | 0.89 | 2.91 | -3.11 | 1.12 | 1.99 |
|  | 2 | SWU11013 | DPL0041 | 13 | CGR5331 | SHIN1462 | 5.52 | 1.81 | 1.32 | 3.08 | -2.82 | -0.68 | 3.50 |
|  | 6 | MUSB1144 | BNL3650 | 13 | CGR5331 | SHIN1462 | 7.15 | 2.59 | 1.11 | 3.70 | -3.28 | 0.76 | 2.52 |
|  | 6 | SWU19656 | CGR5124 | 14 | HAU0883 | CIR228 | 7.03 | 3.10 | 1.00 | 4.02 | -2.81 | 0.02 | 2.79 |
|  | 9 | SWU15413 | NAU0483 | 14 | DPL0502 | ICR00401 | 5.30 | 1.43 | 1.99 | 2.78 | -3.46 | -0.90 | 4.37 |
|  | 1 | Gh529 | SWU17434 | 14 | HAU2482 | NAU4045 | 6.21 | 2.84 | 0.95 | -4.70 | 3.28 | 0.06 | -3.34 |
|  | 14 | ICR03943 | ICR12281 | 14 | ICR03943 | ICR12281 | 6.60 | 2.24 | 1.28 | 4.27 | -3.65 | -0.65 | 4.30 |
|  | 8 | HAU0810 | TMB2904 | 15 | **NAU3736** | **SWU11691** | 5.30 | 1.51 | 1.75 | -2.80 | 3.47 | 0.42 | -3.89 |
|  | 2 | TMB1268 | SWU11976 | 16 | NAU747 | HAU1129 | 6.03 | 2.81 | 0.75 | 3.83 | -2.28 | -0.26 | 2.55 |
|  | 12 | SWU16858 | TMB0327 | 16 | SWU18366 | SWU18579 | 5.21 | 1.82 | 1.34 | 3.10 | -2.55 | -1.11 | 3.66 |
|  | 1 | Gh120 | Gh398 | 18 | SWU21718 | SWU0738 | 5.23 | 1.63 | 1.21 | 2.92 | -2.99 | -0.16 | 3.15 |
|  | 15 | **NAU3736** | **SWU11691** | 18 | SWU0738 | ICR02849 | 6.19 | 2.45 | 1.75 | 3.62 | -2.51 | -1.78 | 4.29 |
|  | 13 | SWU13032 | HAU2850 | 19 | NAU2816 | PGML4342 | 6.27 | 2.00 | 0.85 | 3.24 | -2.78 | 0.44 | 2.34 |
|  | 15 | NAU3736 | SWU11691 | 19 | SWU17789 | SWU17882 | 5.38 | 0.91 | 1.23 | -2.19 | 3.39 | -0.65 | -2.74 |
|  | 12 | HAU3373 | CGR6847 | 20 | SWU20027 | Gh187 | 5.18 | 1.62 | 1.63 | -2.91 | 2.78 | 1.26 | -4.03 |
|  | 8 | DC20094 | Gh197 | 21 | SWU16489 | SWU16360 | 5.62 | 2.46 | 0.72 | -3.64 | 2.45 | -0.09 | -2.35 |
|  | 12 | ICR03107 | HAU3373 | 21 | SWU16361 | SWU16408 | 5.26 | 1.07 | 2.22 | -2.36 | 3.66 | 0.90 | -4.56 |
|  | 2 | SWU11976 | SWU12001 | 21 | BNL3171 | HAU2937 | 11.90 | 5.55 | 2.87 | 5.43 | -3.70 | -1.70 | 5.40 |
|  | 12 | DPL0400 | HAU2173 | 22 | BNL4030 | NAU2026 | 5.88 | 1.40 | 1.61 | 2.75 | -3.49 | -0.21 | 3.70 |
|  | 18 | SWU0738 | ICR02849 | 23 | **BNL3482** | **HAU0244** | 5.44 | 2.06 | 0.94 | -3.50 | 3.01 | -0.14 | -2.87 |
|  | 23 | HAU1758 | SHIN1076 | 23 | **BNL3482** | **HAU0244** | 6.30 | 3.01 | 1.40 | 4.15 | -2.26 | -1.86 | 4.13 |
|  | 2 | DPL0217 | CGR6695 | 23 | PGML1434 | MUSB994 | 8.83 | 3.83 | 2.87 | 4.50 | -3.46 | -1.97 | 5.43 |
|  | 1 | SWU11632 | SWU21958 | 23 | NAU3588 | NAU5373a | 5.15 | 2.16 | 1.13 | -3.42 | 2.59 | 0.74 | -3.33 |
|  | 1 | DPL0790 | ICR03725 | 24 | PGML1207 | Gh54 | 5.00 | 2.01 | 1.13 | -3.34 | 2.67 | 0.67 | -3.35 |
|  | 24 | Gh54 | Gh454 | 24 | HAU3076 | SWU13121 | 6.90 | 2.14 | 1.34 | 4.36 | -3.78 | 0.63 | 3.16 |
|  | 17 | SWU12838a | HAU1413 | 25 | NAU4964 | HAU1355 | 8.15 | 2.51 | 1.68 | 3.70 | -3.60 | -0.12 | 3.72 |
|  | 10 | NAU3395 | CAU0234 | 26 | CGR6477 | PGML2562 | 5.70 | 1.69 | 1.90 | 3.00 | -3.47 | -0.75 | 4.22 |
|  | 10 | HAU0635 | PGML4154 | 28 | BNL3545 | PGML3983 | 6.36 | 2.01 | 2.38 | 3.25 | -3.26 | -1.65 | 4.92 |
|  | 26 | CGR6477 | PGML2562 | 29 | DPL0171 | Gh499 | 6.54 | 2.32 | 0.93 | 3.49 | -2.65 | -0.13 | 2.77 |
|  | 1 | Gh529 | SWU17434 | 30 | TMB1638 | CGR6812 | 5.97 | 2.22 | 0.79 | -3.41 | 2.80 | -0.91 | -1.89 |
|  | 16 | CGR5506 | CGR5925 | 31 | SWU16780 | SWU16735 | 5.15 | 1.39 | 0.85 | -2.71 | 2.89 | -0.74 | -2.15 |
|  | 1 | SWU21958 | NAU0748 | 33 | BNL3661 | PGML4891 | 5.29 | 2.18 | 1.13 | -3.43 | 2.77 | 0.45 | -3.22 |
|  | 21 | SWU16493 | SWU16489 | 33 | BNL3661 | PGML4891 | 7.20 | 2.24 | 1.31 | 3.47 | -3.47 | 0.52 | 2.95 |
|  | 7 | HAU2530 | CGR6586 | 34 | JESPR297 | ICR00647 | 6.81 | 2.37 | 1.64 | 3.52 | -3.67 | 0.16 | 3.52 |
|  | 12 | HAU3373 | CGR6847 | 34 | JESPR297 | ICR00647 | 5.79 | 2.35 | 0.88 | -3.53 | 2.85 | -0.39 | -2.45 |
|  | 1 | Gh398 | CGR6129 | 35 | NAU2139 | TMB1152 | 5.69 | 2.46 | 0.76 | -3.61 | 2.71 | -0.60 | -2.11 |
|  | 21 | BNL1552 | CGR5148 | 36 | CGR5548 | SWU20700 | 6.21 | 2.22 | 1.54 | 3.48 | -3.26 | -0.63 | 3.89 |
|  | 25 | SWU19676 | NAU2968 | 36 | CGR5548 | SWU20700 | 5.08 | 2.01 | 1.09 | 3.27 | -2.57 | -0.66 | 3.23 |
|  | 7 | CGR5372 | C2_0046 | 36 | SWU20658 | CGR6154 | 6.22 | 1.79 | 1.64 | 3.10 | -3.42 | -0.45 | 3.87 |
|  | 32 | TMB0071 | HAU1000 | 36 | SWU20658 | CGR6154 | 5.82 | 2.06 | 1.67 | -3.30 | 2.78 | 1.33 | -4.12 |
|  | 23 | NAU5373b | HAU2648 | 37 | DPL0131 | DPL0777 | 9.55 | 3.53 | 1.84 | -4.31 | 3.79 | 0.07 | -3.87 |
|  | 25 | NAU2968 | DPL0377 | 37 | DPL0131 | DPL0777 | 8.31 | 2.65 | 2.19 | 3.80 | -3.99 | -0.42 | 4.41 |
|  | 12 | BNL3261 | Gh568 | 37 | DPL0131 | DPL0777 | 5.66 | 1.75 | 1.69 | 3.07 | -3.24 | -0.76 | 3.99 |
|  | 17 | HAU1413 | CGR5576 | 38 | NAU2450 | PGML1942 | 5.82 | 1.90 | 1.57 | 3.22 | -3.10 | -0.85 | 3.95 |
|  | 10 | HAU0635 | PGML4154 | 38 | NAU2450 | PGML1942 | 7.76 | 2.77 | 1.40 | 3.83 | -3.13 | -0.36 | 3.49 |
|  | 37 | DPL0131 | DPL0777 | 38 | NAU2450 | PGML1942 | 5.96 | 1.50 | 1.56 | 2.83 | -3.31 | -0.43 | 3.74 |
|  | 36 | SWU20658 | CGR6154 | 39 | HAU2022 | BNL0827 | 5.93 | 2.40 | 1.11 | 3.56 | -2.21 | -1.17 | 3.38 |
|  | 1 | ICR03295 | SWU10912 | 39 | PGML0844 | NAU5480 | 5.69 | 1.72 | 1.34 | 3.00 | -3.14 | -0.19 | 3.32 |
|  | 29 | DPL0171 | Gh499 | 39 | DPL0270 | SWU16437 | 6.04 | 2.22 | 1.28 | -3.42 | 2.83 | 0.59 | -3.42 |
|  | 3 | NAU2742 | SWU12841 | 39 | DPL0270 | SWU16437 | 6.65 | 2.57 | 0.91 | 3.71 | -2.84 | 0.30 | 2.53 |
|  | 12 | DPL0400 | HAU2173 | 39 | SWU16437 | SWU16432 | 5.93 | 2.51 | 0.98 | -3.63 | 2.19 | 0.92 | -3.11 |
| LY | 4 | BNL1167 | JESPR234 | 4 | SWU16783 | SWU18876 | 5.74 | 1.87 | 1.88 | -1.30 | 1.37 | 0.43 | -1.80 |
|  | 5 | Gh260 | PGML0120 | 5 | Gh260 | PGML0120 | 5.46 | 2.42 | 0.53 | 1.55 | -1.07 | 0.13 | 0.94 |
|  | 2 | TMB1268 | SWU11976 | 6 | SWU19656 | CGR5124 | 6.97 | 2.66 | 1.68 | 1.53 | -1.16 | -0.51 | 1.68 |
|  | 4 | JESPR234 | BNL530 | 6 | SWU19184 | DPL0847 | 5.01 | 0.87 | 1.98 | 0.87 | -1.39 | -0.37 | 1.76 |
|  | 6 | MUSB1144 | BNL3650 | 8 | DC20094 | Gh197 | 5.02 | 2.27 | 0.67 | -1.42 | 0.95 | -0.03 | -0.92 |
|  | 6 | MUSB1144 | BNL3650 | 12 | C2_0115 | SWU16858 | 5.01 | 1.50 | 1.17 | -1.15 | 1.18 | 0.12 | -1.30 |
|  | 1 | PGML2498 | SWU14490 | 12 | HAU3373 | CGR6847 | 5.63 | 2.79 | 0.69 | -1.56 | 0.87 | 0.16 | -1.02 |
|  | 10 | SWU13030 | NAU4967 | 12 | DPL0400 | HAU2173 | 5.54 | 1.88 | 1.17 | 1.30 | -1.13 | -0.18 | 1.32 |
|  | 8 | HAU0810 | TMB2904 | 12 | DPL0400 | HAU2173 | 5.80 | 2.49 | 0.97 | -1.48 | 1.01 | 0.18 | -1.20 |
|  | 6 | MUSB1144 | BNL3650 | 13 | CGR5331 | SHIN1462 | 6.24 | 2.24 | 1.06 | 1.40 | -1.24 | 0.12 | 1.13 |
|  | 1 | Gh529 | SWU17434 | 14 | HAU2482 | NAU4045 | 5.99 | 2.69 | 1.00 | -1.88 | 1.31 | 0.13 | -1.44 |
|  | 6 | DC40417 | MUSB1164 | 14 | HAU2482 | NAU4045 | 6.92 | 2.81 | 0.72 | 1.91 | -1.35 | 0.47 | 0.88 |
|  | 10 | NAU3395 | CAU0234 | 14 | ICR03943 | ICR12281 | 5.09 | 2.38 | 0.53 | -1.51 | 0.79 | 0.10 | -0.89 |
|  | 14 | ICR03943 | ICR12281 | 14 | ICR03943 | ICR12281 | 6.80 | 2.60 | 1.06 | 1.87 | -1.28 | -0.39 | 1.67 |
|  | 12 | DPL0400 | HAU2173 | 16 | HAU3081 | NAU747 | 5.03 | 2.45 | 0.60 | 1.47 | -0.84 | -0.08 | 0.93 |
|  | 2 | TMB1268 | SWU11976 | 16 | NAU747 | HAU1129 | 5.14 | 2.35 | 0.83 | 1.44 | -0.85 | -0.31 | 1.16 |
|  | 15 | **NAU3736** | **SWU11691** | 18 | SWU0738 | ICR02849 | 7.34 | 2.93 | 1.77 | 1.62 | -1.12 | -0.62 | 1.74 |
|  | 13 | SWU13032 | HAU2850 | 19 | NAU2816 | PGML4342 | 5.59 | 1.90 | 0.78 | 1.29 | -1.02 | 0.01 | 1.00 |
|  | 12 | HAU3373 | CGR6847 | 20 | SWU20027 | Gh187 | 5.25 | 1.64 | 1.59 | -1.20 | 1.12 | 0.52 | -1.63 |
|  | 2 | SWU11976 | SWU12001 | 21 | BNL3171 | HAU2937 | 11.39 | 4.84 | 3.18 | 2.08 | -1.56 | -0.78 | 2.34 |
|  | 12 | DPL0400 | HAU2173 | 22 | BNL4030 | NAU2026 | 5.32 | 1.64 | 1.26 | 1.21 | -1.17 | -0.24 | 1.41 |
|  | 3 | NAU2742 | SWU12841 | 23 | HAU1758 | SHIN1076 | 5.61 | 2.30 | 1.04 | -1.45 | 1.07 | 0.20 | -1.27 |
|  | 18 | SWU0738 | ICR02849 | 23 | BNL3482 | HAU0244 | 5.16 | 1.83 | 0.98 | -1.35 | 1.17 | 0.10 | -1.27 |
|  | 23 | HAU1758 | SHIN1076 | 23 | BNL3482 | HAU0244 | 6.90 | 3.34 | 1.36 | 1.80 | -0.87 | -0.78 | 1.65 |
|  | 5 | Gh260 | PGML0120 | 23 | ICR06429 | SWU0506 | 5.01 | 1.20 | 1.61 | -1.17 | 1.41 | 0.38 | -1.79 |
|  | 2 | DPL0217 | CGR6695 | 23 | PGML1434 | MUSB994 | 9.28 | 3.57 | 3.31 | 1.77 | -1.57 | -0.81 | 2.39 |
|  | 1 | SWU11632 | SWU21958 | 23 | NAU3588 | NAU5373a | 5.50 | 2.51 | 1.13 | -1.51 | 0.96 | 0.44 | -1.40 |
|  | 1 | DPL0790 | ICR03725 | 24 | PGML1207 | Gh54 | 6.01 | 2.60 | 1.04 | -1.56 | 1.02 | 0.30 | -1.32 |
|  | 24 | Gh54 | Gh454 | 24 | HAU3076 | SWU13121 | 6.23 | 2.20 | 1.14 | 1.74 | -1.37 | -0.01 | 1.39 |
|  | 7 | HAU1367 | NAU3181 | 25 | DPL0377 | SWU19413 | 6.18 | 2.76 | 1.24 | 1.56 | -1.03 | -0.40 | 1.43 |
|  | 17 | SWU12838a | HAU1413 | 25 | NAU4964 | HAU1355 | 7.03 | 2.40 | 1.34 | 1.47 | -1.23 | -0.20 | 1.43 |
|  | 10 | HAU0635 | PGML4154 | 28 | BNL3545 | PGML3983 | 6.54 | 2.22 | 2.15 | 1.40 | -1.25 | -0.67 | 1.92 |
|  | 26 | CGR6477 | PGML2562 | 29 | DPL0171 | Gh499 | 6.75 | 2.49 | 1.03 | 1.48 | -1.02 | -0.26 | 1.28 |
|  | 1 | Gh529 | SWU17434 | 30 | TMB1638 | CGR6812 | 5.21 | 1.68 | 0.84 | -1.21 | 1.15 | -0.25 | -0.90 |
|  | 1 | ICR03295 | SWU10912 | 31 | SWU16780 | SWU16735 | 5.37 | 2.20 | 0.75 | 1.39 | -0.91 | -0.09 | 1.00 |
|  | 16 | CGR5506 | CGR5925 | 31 | SWU16780 | SWU16735 | 5.03 | 1.36 | 0.86 | -1.10 | 1.14 | -0.16 | -0.98 |
|  | 21 | SWU16493 | SWU16489 | 33 | BNL3661 | PGML4891 | 6.15 | 2.14 | 1.17 | 1.39 | -1.23 | -0.05 | 1.28 |
|  | 7 | HAU2530 | CGR6586 | 34 | DPL0897 | SWU20341 | 5.08 | 1.55 | 1.61 | 1.16 | -1.36 | -0.17 | 1.53 |
|  | 25 | BNL3098 | HAU1224 | 34 | JESPR297 | ICR00647 | 5.34 | 2.09 | 0.60 | -1.35 | 1.01 | -0.47 | -0.53 |
|  | 1 | Gh398 | CGR6129 | 35 | NAU2139 | TMB1152 | 5.01 | 2.40 | 0.60 | -1.46 | 0.89 | 0.02 | -0.90 |
|  | 21 | BNL1552 | CGR5148 | 36 | CGR5548 | SWU20700 | 6.16 | 2.03 | 1.89 | 1.36 | -1.32 | -0.51 | 1.82 |
|  | 25 | SWU19676 | NAU2968 | 36 | CGR5548 | SWU20700 | 6.23 | 2.61 | 1.01 | 1.53 | -1.07 | -0.18 | 1.24 |
|  | 7 | CGR5372 | C2_0046 | 36 | SWU20658 | CGR6154 | 6.15 | 1.86 | 1.43 | 1.29 | -1.31 | -0.17 | 1.48 |
|  | 32 | TMB0071 | HAU1000 | 36 | SWU20658 | CGR6154 | 5.68 | 2.13 | 1.52 | -1.37 | 0.99 | 0.63 | -1.63 |
|  | 23 | NAU5373b | HAU2648 | 37 | DPL0131 | DPL0777 | 9.56 | 3.91 | 1.55 | -1.87 | 1.35 | 0.17 | -1.52 |
|  | 25 | NAU2968 | DPL0377 | 37 | DPL0131 | DPL0777 | 8.37 | 3.06 | 1.88 | 1.67 | -1.42 | -0.31 | 1.73 |
|  | 12 | BNL3261 | Gh568 | 37 | DPL0131 | DPL0777 | 5.72 | 2.05 | 1.38 | 1.36 | -1.17 | -0.32 | 1.49 |
|  | 17 | HAU1413 | CGR5576 | 38 | NAU2450 | PGML1942 | 5.88 | 1.75 | 1.65 | 1.27 | -1.24 | -0.45 | 1.69 |
|  | 10 | HAU0635 | PGML4154 | 38 | NAU2450 | PGML1942 | 6.41 | 2.06 | 1.40 | 1.35 | -1.18 | -0.31 | 1.49 |
|  | 36 | SWU20658 | CGR6154 | 39 | HAU2022 | BNL0827 | 6.89 | 2.60 | 1.20 | 1.51 | -0.99 | -0.43 | 1.42 |
|  | 1 | ICR03295 | SWU10912 | 39 | PGML0844 | NAU5480 | 5.83 | 2.08 | 1.16 | 1.35 | -1.14 | -0.17 | 1.31 |
|  | 21 | CGR6521 | Gh450 | 39 | NAU5480 | DPL0270 | 5.13 | 2.48 | 0.37 | -1.49 | 0.78 | -0.20 | -0.58 |
|  | 29 | DPL0171 | Gh499 | 39 | DPL0270 | SWU16437 | 6.17 | 1.99 | 1.38 | -1.33 | 1.25 | 0.18 | -1.43 |
|  | 3 | NAU2742 | SWU12841 | 39 | DPL0270 | SWU16437 | 7.37 | 3.11 | 0.83 | 1.67 | -1.07 | 0.04 | 1.03 |
|  | 12 | DPL0400 | HAU2173 | 39 | SWU16437 | SWU16432 | 5.25 | 1.97 | 1.09 | -1.32 | 0.94 | 0.40 | -1.35 |
| BNP | 1 | SWU14514 | Gh120 | 1 | ICR03295 | SWU10912 | 5.02 | 2.99 | 0.59 | 0.84 | -0.43 | -0.05 | 0.49 |
|  | 1 | DPL0790 | ICR03725 | 2 | DPL0217 | CGR6695 | 5.01 | 3.28 | 0.20 | 0.88 | -0.30 | 0.21 | 0.10 |
|  | 5 | Gh260 | PGML0120 | 5 | Gh260 | PGML0120 | 7.71 | 4.76 | 0.71 | 1.13 | -0.47 | -0.08 | 0.55 |
|  | 1 | DPL0790 | ICR03725 | 6 | CGR5355 | SWU19656 | 5.58 | 3.21 | 1.02 | -0.88 | 0.48 | 0.21 | -0.69 |
|  | 2 | TMB1268 | SWU11976 | 6 | SWU19656 | CGR5124 | 8.68 | 4.94 | 1.19 | 1.06 | -0.57 | -0.12 | 0.69 |
|  | 6 | MUSB1144 | BNL3650 | 8 | DC20094 | Gh197 | 8.27 | 4.92 | 0.72 | -1.06 | 0.55 | -0.13 | -0.42 |
|  | 3 | NAU2742 | SWU12841 | 8 | HAU0810 | TMB2904 | 5.74 | 2.41 | 0.89 | -0.74 | 0.62 | -0.19 | -0.44 |
|  | 3 | SWU12840 | NAU2742 | 12 | ICR03107 | HAU3373 | 5.55 | 2.89 | 0.93 | 0.82 | -0.57 | 0.00 | 0.58 |
|  | 6 | CGR6749 | NAU3186 | 12 | HAU3373 | CGR6847 | 5.14 | 2.59 | 0.99 | 0.77 | -0.48 | -0.16 | 0.64 |
|  | 1 | NAU2697 | SWU0320 | 12 | CGR6847 | SWU17197 | 5.28 | 3.25 | 0.33 | -0.86 | 0.34 | -0.01 | -0.33 |
|  | 10 | SWU13030 | NAU4967 | 12 | DPL0400 | HAU2173 | 8.98 | 4.09 | 1.39 | 0.97 | -0.74 | 0.10 | 0.63 |
|  | 8 | HAU0810 | TMB2904 | 12 | DPL0400 | HAU2173 | 7.48 | 4.87 | 0.47 | -1.06 | 0.45 | -0.11 | -0.34 |
|  | 6 | SWU19656 | CGR5124 | 14 | HAU0883 | CIR228 | 7.18 | 4.69 | 0.44 | 1.03 | -0.44 | 0.16 | 0.28 |
|  | 4 | BNL1167 | JESPR234 | 14 | DPL0502 | ICR00401 | 5.21 | 3.53 | 0.23 | -0.90 | 0.32 | -0.13 | -0.18 |
|  | 1 | Gh529 | SWU17434 | 14 | HAU2482 | NAU4045 | 6.23 | 3.64 | 0.56 | -1.11 | 0.59 | -0.23 | -0.36 |
|  | 9 | MUSS139 | PGML2830 | 14 | HAU2482 | NAU4045 | 5.27 | 2.51 | 0.87 | 0.92 | -0.74 | 0.16 | 0.58 |
|  | 12 | DPL0400 | HAU2173 | 14 | HAU2482 | NAU4045 | 5.97 | 3.22 | 0.62 | 1.05 | -0.61 | 0.09 | 0.53 |
|  | 8 | HAU0810 | TMB2904 | 14 | ICR03943 | ICR12281 | 5.66 | 2.99 | 0.82 | -0.90 | 0.58 | -0.03 | -0.55 |
|  | 14 | ICR03943 | ICR12281 | 14 | ICR03943 | ICR12281 | 5.78 | 3.45 | 0.43 | 1.06 | -0.50 | -0.03 | 0.53 |
|  | 12 | DPL0400 | HAU2173 | 16 | HAU3081 | NAU747 | 10.85 | 6.66 | 0.94 | 1.23 | -0.64 | 0.18 | 0.46 |
|  | 2 | TMB1268 | SWU11976 | 16 | NAU747 | HAU1129 | 8.29 | 4.55 | 1.12 | 1.02 | -0.60 | -0.04 | 0.64 |
|  | 4 | SWU16783 | SWU18876 | 18 | SWU0738 | ICR02849 | 5.05 | 3.05 | 0.41 | 0.84 | -0.42 | 0.10 | 0.32 |
|  | 15 | **NAU3736** | **SWU11691** | 18 | SWU0738 | ICR02849 | 6.23 | 3.21 | 1.07 | 0.86 | -0.53 | -0.13 | 0.66 |
|  | 17 | HAU1413 | CGR5576 | 19 | NAU2816 | PGML4342 | 5.12 | 3.64 | 0.16 | 0.90 | -0.25 | 0.08 | 0.17 |
|  | 10 | SWU19932 | HAU0635 | 20 | HAU1314 | SWU20035 | 6.75 | 4.46 | 0.47 | -1.01 | 0.35 | 0.09 | -0.44 |
|  | 12 | HAU3373 | CGR6847 | 20 | SWU20027 | Gh187 | 5.31 | 2.84 | 0.65 | -0.80 | 0.48 | -0.01 | -0.47 |
|  | 13 | CGR5331 | SHIN1462 | 20 | SWU20027 | Gh187 | 5.50 | 2.98 | 0.53 | 0.83 | -0.49 | 0.28 | 0.21 |
|  | 6 | SWU19656 | CGR5124 | 21 | CGR5148 | SHIN0337 | 5.20 | 2.82 | 0.54 | 0.80 | -0.44 | 0.03 | 0.41 |
|  | 10 | SWU19932 | HAU0635 | 21 | CGR5148 | SHIN0337 | 5.06 | 2.91 | 0.49 | 0.82 | -0.35 | -0.10 | 0.45 |
|  | 8 | DC20094 | Gh197 | 21 | SWU16489 | SWU16360 | 7.82 | 4.83 | 0.56 | -1.06 | 0.50 | -0.13 | -0.37 |
|  | 16 | HAU3081 | NAU747 | 21 | SWU16361 | SWU16408 | 5.43 | 2.27 | 1.26 | 0.72 | -0.68 | 0.05 | 0.63 |
|  | 12 | DPL0400 | HAU2173 | 22 | BNL4030 | NAU2026 | 7.46 | 2.89 | 1.24 | 0.82 | -0.73 | 0.18 | 0.55 |
|  | 16 | HAU1129 | C2_0011B | 23 | DC40286 | PGML1434 | 5.71 | 3.51 | 0.56 | 0.89 | -0.47 | 0.08 | 0.40 |
|  | 2 | DPL0217 | CGR6695 | 23 | PGML1434 | MUSB994 | 5.68 | 2.70 | 1.00 | 0.78 | -0.62 | 0.08 | 0.54 |
|  | 1 | SWU14616 | SWU14077 | 23 | HAU2648 | SWU20501 | 6.23 | 3.79 | 0.66 | 0.93 | -0.45 | -0.05 | 0.50 |
|  | 24 | Gh54 | Gh454 | 24 | HAU3076 | SWU13121 | 6.43 | 3.76 | 0.58 | 1.09 | -0.61 | 0.03 | 0.58 |
|  | 3 | NAU2742 | SWU12841 | 25 | NAU4964 | HAU1355 | 5.20 | 2.31 | 0.79 | 0.73 | -0.59 | 0.17 | 0.42 |
|  | 17 | SWU12838a | HAU1413 | 25 | NAU4964 | HAU1355 | 14.06 | 6.92 | 1.51 | 1.27 | -0.81 | 0.21 | 0.60 |
|  | 12 | HAU3373 | CGR6847 | 26 | HAU1571 | CGR6477 | 5.80 | 2.76 | 1.17 | 0.79 | -0.60 | -0.06 | 0.66 |
|  | 10 | HAU0635 | PGML4154 | 28 | BNL3545 | PGML3983 | 7.43 | 4.14 | 0.97 | 0.97 | -0.55 | -0.06 | 0.61 |
|  | 26 | CGR6477 | PGML2562 | 29 | DPL0171 | Gh499 | 7.47 | 3.41 | 1.12 | 0.88 | -0.57 | -0.09 | 0.66 |
|  | 6 | BNL3650 | TMB2940 | 29 | Gh111 | Gh27 | 5.59 | 3.03 | 0.67 | 0.83 | -0.51 | 0.06 | 0.45 |
|  | 25 | NAU4964 | HAU1355 | 30 | TMB1638 | CGR6812 | 5.28 | 3.24 | 0.32 | -0.88 | 0.37 | -0.05 | -0.32 |
|  | 1 | CGR6129 | ICR03295 | 31 | SWU16780 | SWU16735 | 5.25 | 3.32 | 0.57 | 0.87 | -0.41 | -0.06 | 0.47 |
|  | 16 | CGR5506 | CGR5925 | 31 | **SWU16735** | **SWU16755** | 5.60 | 3.21 | 0.58 | -0.86 | 0.50 | -0.15 | -0.35 |
|  | 30 | TMB1638 | CGR6812 | 31 | SWU16730 | SWU16721 | 6.20 | 3.86 | 0.46 | -0.95 | 0.35 | 0.10 | -0.45 |
|  | 13 | NAU3398 | CGR5331 | 33 | BNL3661 | PGML4891 | 5.34 | 2.78 | 0.67 | -0.80 | 0.54 | -0.15 | -0.39 |
|  | 21 | SWU16493 | SWU16489 | 33 | BNL3661 | PGML4891 | 8.01 | 3.40 | 1.33 | 0.89 | -0.72 | 0.09 | 0.63 |
|  | 25 | BNL3098 | HAU1224 | 34 | JESPR297 | ICR00647 | 5.14 | 3.08 | 0.45 | -0.83 | 0.44 | -0.18 | -0.26 |
|  | 10 | SWU19932 | HAU0635 | 35 | NAU2139 | TMB1152 | 5.33 | 2.71 | 0.66 | -0.83 | 0.55 | -0.30 | -0.25 |
|  | 1 | Gh398 | CGR6129 | 35 | NAU2139 | TMB1152 | 7.98 | 4.39 | 1.32 | -1.01 | 0.54 | 0.21 | -0.75 |
|  | 12 | CGR6847 | SWU17197 | 35 | NAU2139 | TMB1152 | 6.36 | 2.66 | 1.11 | 0.79 | -0.72 | 0.30 | 0.42 |
|  | 7 | CGR5372 | C2_0046 | 36 | CGR5548 | SWU20700 | 6.35 | 3.60 | 0.94 | 0.93 | -0.49 | -0.15 | 0.65 |
|  | 25 | SWU19676 | NAU2968 | 36 | CGR5548 | SWU20700 | 6.91 | 4.35 | 0.63 | 1.00 | -0.43 | -0.06 | 0.50 |
|  | 21 | BNL1552 | CGR5148 | 36 | CER0167 | SWU20658 | 5.03 | 2.35 | 0.86 | 0.74 | -0.54 | -0.03 | 0.57 |
|  | 32 | TMB0071 | HAU1000 | 36 | CER0167 | SWU20658 | 6.63 | 3.34 | 0.93 | -0.88 | 0.62 | -0.14 | -0.49 |
|  | 33 | BNL3661 | PGML4891 | 36 | SWU20658 | CGR6154 | 6.99 | 3.30 | 1.04 | 0.87 | -0.66 | 0.13 | 0.53 |
|  | 23 | NAU5373b | HAU2648 | 37 | DPL0131 | DPL0777 | 10.29 | 5.74 | 1.22 | -1.16 | 0.73 | -0.19 | -0.54 |
|  | 25 | NAU2968 | DPL0377 | 37 | DPL0131 | DPL0777 | 5.52 | 2.61 | 1.37 | 0.78 | -0.29 | -0.50 | 0.79 |
|  | 21 | CGR5748 | PGML2500 | 37 | DPL0131 | DPL0777 | 5.21 | 2.96 | 0.63 | 0.83 | -0.47 | 0.00 | 0.46 |
|  | 10 | HAU0635 | PGML4154 | 38 | NAU2450 | PGML1942 | 7.11 | 3.60 | 0.86 | 0.91 | -0.57 | 0.06 | 0.51 |
|  | 37 | DPL0131 | DPL0777 | 38 | NAU2450 | PGML1942 | 6.55 | 3.57 | 0.92 | 0.91 | -0.52 | -0.09 | 0.61 |
|  | 31 | CGR6772 | HAU0355 | 38 | NAU2450 | PGML1942 | 5.44 | 2.76 | 0.56 | 0.79 | -0.49 | 0.16 | 0.33 |
|  | 1 | SWU14514 | Gh120 | 39 | NAU5480 | DPL0270 | 5.84 | 3.43 | 0.67 | -0.88 | 0.42 | 0.09 | -0.51 |
|  | 21 | CGR6521 | Gh450 | 39 | NAU5480 | DPL0270 | 6.15 | 3.90 | 0.40 | -0.95 | 0.42 | -0.13 | -0.28 |
|  | 29 | Gh111 | Gh27 | 39 | DPL0270 | SWU16437 | 7.72 | 3.52 | 1.04 | -0.90 | 0.65 | -0.12 | -0.53 |
|  | 3 | NAU2742 | SWU12841 | 39 | DPL0270 | SWU16437 | 6.34 | 3.43 | 0.70 | 0.89 | -0.53 | 0.08 | 0.45 |
| BW | 1 | ICR03725 | ICR03724 | 3 | SWU12840 | NAU2742 | 6.11 | 4.12 | 0.01 | 0.10 | 0.00 | 0.00 | 0.00 |
|  | 3 | SWU12840 | NAU2742 | 7 | CGR5372 | C2_0046 | 6.35 | 4.00 | 0.21 | 0.10 | -0.03 | 0.00 | 0.03 |
|  | 4 | BNL530 | SWU16781 | 14 | HAU0883 | CIR228 | 6.13 | 3.98 | 0.06 | -0.09 | -0.02 | 0.01 | 0.00 |
|  | 1 | SWU14616 | SWU14077 | 18 | SWU0738 | ICR02849 | 6.56 | 3.96 | 0.01 | 0.10 | -0.02 | 0.00 | 0.01 |
|  | 12 | HAU3373 | CGR6847 | 21 | CGR6521 | Gh450 | 5.34 | 3.55 | 0.03 | -0.09 | 0.00 | 0.01 | -0.01 |
|  | 19 | SWU17789 | SWU17882 | 24 | Gh54 | Gh454 | 5.34 | 3.39 | 0.05 | 0.09 | -0.01 | 0.02 | -0.01 |
|  | 10 | NAU3395 | CAU0234 | 32 | HAU1000 | TMB1931 | 5.56 | 3.05 | 0.65 | 0.08 | -0.05 | 0.03 | 0.03 |
|  | 20 | SWU20027 | Gh187 | 34 | DPL0897 | SWU20341 | 6.41 | 3.81 | 0.53 | 0.09 | -0.02 | -0.03 | 0.05 |
|  | 13 | CER0165 | SWU13032 | 37 | HAU0423 | JESPR154 | 5.29 | 3.29 | 0.31 | 0.08 | -0.01 | -0.03 | 0.03 |
|  | 6 | MUSB1144 | BNL3650 | 38 | **NAU2450** | **PGML1942** | 6.12 | 3.63 | 0.22 | 0.09 | -0.03 | 0.02 | 0.01 |
|  | 4 | BNL1167 | JESPR234 | 39 | NAU5480 | DPL0270 | 5.65 | 3.70 | 0.09 | 0.09 | -0.02 | 0.01 | 0.00 |
| LP | 1 | **SWU11632** | **SWU21958** | 1 | SWU21958 | NAU0748 | 6.97 | 1.77 | 0.03 | 0.96 | -0.09 | 0.08 | 0.01 |
|  | 1 | PGML2498 | SWU14490 | 10 | HAU0635 | PGML4154 | 5.37 | 3.48 | 0.39 | 0.43 | -0.01 | 0.18 | -0.17 |
|  | 4 | SWU16783 | SWU18876 | 12 | ICR03107 | HAU3373 | 5.33 | 3.45 | 0.20 | 0.42 | 0.04 | 0.09 | -0.14 |
|  | 8 | DC20094 | Gh197 | 13 | SWU13032 | HAU2850 | 6.30 | 3.60 | 0.79 | -0.43 | -0.02 | -0.23 | 0.26 |
|  | 14 | HAU0883 | CIR228 | 14 | DPL0502 | ICR00401 | 7.02 | 3.50 | 0.02 | 0.73 | -0.03 | -0.02 | 0.05 |
|  | 1 | ICR03295 | SWU10912 | 17 | SWU12838a | HAU1413 | 6.02 | 4.24 | 0.09 | -0.46 | 0.03 | -0.09 | 0.06 |
|  | 12 | C2_0115 | SWU16858 | 18 | SWU21718 | SWU0738 | 7.47 | 5.28 | 0.00 | 0.52 | -0.03 | 0.02 | 0.01 |
|  | 12 | COT107 | Gh243 | 20 | SWU20027 | Gh187 | 8.08 | 5.34 | 0.49 | -0.52 | -0.02 | -0.18 | 0.20 |
|  | 14 | ICR01124 | HAU2482 | 21 | CGR5748 | PGML2500 | 5.56 | 3.88 | 0.40 | 0.54 | -0.13 | 0.24 | -0.12 |
|  | 12 | DPL0400 | HAU2173 | 21 | SWU16489 | SWU16360 | 6.07 | 4.21 | 0.06 | 0.47 | -0.06 | 0.08 | -0.02 |
|  | 16 | HAU3081 | NAU747 | 23 | HAU1758 | SHIN1076 | 5.18 | 3.68 | 0.08 | 0.44 | 0.01 | 0.07 | -0.08 |
|  | 9 | CGR5009 | CGR6771 | 23 | Gh327 | ICR06429 | 5.83 | 3.99 | 0.13 | -0.49 | 0.11 | -0.11 | 0.01 |
|  | 8 | HAU0810 | TMB2904 | 23 | DC40286 | PGML1434 | 6.07 | 4.35 | 0.00 | -0.47 | 0.01 | -0.03 | 0.02 |
|  | 10 | HAU0635 | PGML4154 | 25 | DPL0377 | SWU19413 | 5.19 | 3.23 | 0.44 | 0.40 | -0.12 | 0.21 | -0.09 |
|  | 4 | BNL530 | SWU16781 | 25 | BNL3098 | HAU1224 | 5.00 | 2.55 | 1.04 | 0.36 | -0.08 | 0.31 | -0.23 |
|  | 20 | SWU20246 | Gh451 | 26 | HAU1571 | CGR6477 | 5.07 | 3.20 | 0.18 | -0.41 | 0.07 | -0.13 | 0.07 |
|  | 2 | SWU11889 | JESPR304 | 26 | **CGR6477** | **PGML2562** | 6.17 | 4.71 | 0.05 | 0.49 | 0.02 | 0.03 | -0.05 |
|  | 23 | **BNL3482** | **HAU0244** | 27 | Gh247 | CGR5867 | 5.03 | 3.56 | 0.12 | -0.45 | -0.12 | 0.07 | 0.05 |
|  | 16 | HAU3081 | NAU747 | 31 | **DPL0057** | **NAU3109** | 5.58 | 3.81 | 0.10 | -0.44 | -0.09 | 0.02 | 0.07 |
|  | 31 | NAU3109 | CGR6772 | 36 | SWU20658 | CGR6154 | 5.08 | 3.09 | 0.42 | 0.40 | -0.05 | 0.20 | -0.15 |
|  | 21 | BNL1552 | CGR5148 | 37 | DPL0131 | DPL0777 | 5.04 | 3.51 | 0.00 | 0.43 | -0.01 | 0.03 | -0.02 |
|  | 2 | DPL0200 | SWU11889 | 37 | HAU0423 | JESPR154 | 5.21 | 3.49 | 0.30 | 0.42 | -0.10 | 0.17 | -0.07 |

Chi and Chj represent the chromosome number of the loci being tested in the analysis

Flanking markers in bold are those flanking M-QTLs identified by inclusive composite interval mapping in additional table S2

AA is the epistatic effect between loci i and j

AAE is the effect of the environmental interaction of epistasis

AAEl, AAE2 and AAE3 indicate the epistatic effects of QTL × environment interactions in E1, E2 and E3, respectively

V(AA)% and V(AAE)%, percentage of the total variation explained by the AA and AAE
