# Supplementary material for: Partial Dominance, Overdominance, Epistasis and QTL by Environment Interactions Contribute to Heterosis in Two Upland Cotton Hybrids
Source: G3 (Bethesda). 2015 Dec 29;6(3):499–507. doi: 10.1534/g3.115.025809 (PMC4777113; doi:10.1534/g3.115.025809)
Supplement: Supporting Information [file supp_g3.115.025809_TableS6.doc]

**Table S6 Epistatic effects and environmental interactions detected for yield and yield components in BCF_1_ and BCVF_1_ populations using two-locus analysis by inclusive composite interval mapping**

| Trait | Chi | Flanking markers | | Chj | Flanking markers | | LOD | V(AA) | V(AAE) | AA | AAE1 | AAE2 | AAE3 |
| --- | --- | --- | --- | --- | --- | --- | --- | --- | --- | --- | --- | --- | --- |
| BCF_1_ population | | | | | | | | | | | | | |
| SY | 7 | SWU10067 | SWU10064 | 11 | ICR01810 | CGR6525 | 7.02 | 1.91 | 3.48 | 1.46 | -1.64 | -1.14 | 2.79 |
|  | 9 | SWU15511 | SWU15413 | 14 | SWU14224 | DPL0565 | 5.27 | 2.28 | 0.85 | 1.60 | -1.32 | 0.16 | 1.15 |
|  | 14 | SWU14224 | DPL0565 | 15 | DC40183 | DC40175 | 5.96 | 1.96 | 1.44 | 1.47 | -1.79 | 0.60 | 1.19 |
|  | 5 | CGR5025 | NBRI0694 | 16 | Gh56 | NAU5120 | 5.57 | 2.09 | 1.45 | 1.52 | -1.75 | 0.69 | 1.06 |
|  | 3 | SWU12732 | SWU12783 | 26 | Gh64 | SWU17257 | 5.37 | 2.37 | 1.42 | -1.64 | 1.18 | 0.57 | -1.75 |
|  | 21 | SWU16651 | SWU16645 | 29 | DC20127 | DPL0252 | 7.75 | 2.83 | 2.11 | 1.77 | -2.09 | 0.56 | 1.53 |
|  | 26 | MGHES31 | HAU1571 | 29 | DC20127 | DPL0252 | 5.65 | 1.66 | 2.53 | 1.36 | -1.53 | -0.80 | 2.33 |
|  | 28 | HAU3071 | CGR5534 | 29 | DPL0252 | BNL3261 | 5.66 | 2.16 | 1.25 | 1.56 | -1.23 | -0.35 | 1.59 |
| LY | 7 | SWU10067 | SWU10064 | 11 | ICR01810 | CGR6525 | 5.72 | 1.61 | 2.78 | 0.55 | -0.62 | -0.40 | 1.02 |
|  | 8 | Gh197 | DC20094 | 14 | CIR228 | BNL2485 | 5.20 | 3.05 | 0.83 | 0.75 | -0.20 | -0.35 | 0.55 |
|  | 5 | SWU13378 | SWU17846 | 16 | PGML1709 | SWU10627 | 5.76 | 2.96 | 1.20 | 0.75 | -0.39 | -0.28 | 0.66 |
|  | 10 | CGR5873 | ICR00093 | 17 | NAU3765 | SWU14627 | 5.01 | 2.74 | 0.79 | 0.75 | -0.42 | -0.13 | 0.55 |
|  | 19 | NAU5330 | Gh72 | 21 | SWU0830 | HAU2004 | 5.73 | 3.61 | 0.30 | 0.85 | -0.28 | -0.02 | 0.31 |
|  | 20 | CGR5548 | SWU20675 | 27 | SWU11384 | ICR11885 | 5.26 | 2.21 | 1.40 | -0.68 | 0.26 | 0.50 | -0.76 |
|  | 5 | TMB1296 | HAU1603 | 27 | ICR11205 | DPL0847a | 5.27 | 2.54 | 1.33 | 0.69 | -0.38 | -0.35 | 0.73 |
|  | 13 | SWU22309 | SWU22324 | 29 | DC20127 | DPL0252 | 5.14 | 3.42 | 0.30 | 0.82 | -0.18 | -0.16 | 0.34 |
|  | 21 | SWU16651 | SWU16645 | 29 | DC20127 | DPL0252 | 7.08 | 3.49 | 1.26 | 0.81 | -0.68 | 0.26 | 0.42 |
|  | 28 | HAU3071 | CGR5534 | 29 | DPL0252 | BNL3261 | 5.70 | 2.06 | 1.40 | 0.62 | -0.54 | -0.15 | 0.69 |
| BNP | 3 | Gh663 | CGR6528 | 8 | HAU1470a | SHIN1341 | 6.61 | 3.58 | 0.71 | -0.46 | 0.17 | -0.29 | 0.11 |
|  | 10 | Gh320 | HAU0635 | 14 | ICR12037 | CGR5675 | 5.02 | 2.93 | 0.39 | -0.41 | 0.21 | -0.14 | -0.07 |
|  | 2 | PGML0700 | SWU12016 | 18 | CIR099 | NAU748 | 5.18 | 1.98 | 1.52 | 0.34 | -0.13 | -0.28 | 0.41 |
|  | 7 | PGML1916 | SWU10864 | 20 | SWU20246 | SWU20501a | 6.06 | 3.90 | 0.54 | 0.53 | 0.15 | 0.15 | -0.30 |
|  | 16 | SWU20341 | DPL0897 | 24 | HAU2504 | SWU13736 | 5.95 | 1.42 | 2.97 | 0.29 | -0.16 | -0.42 | 0.57 |
|  | 1 | NAU2218 | SWU11191 | 24 | SWU13758 | CGR5423 | 6.64 | 3.86 | 0.56 | -0.47 | 0.07 | 0.18 | -0.24 |
|  | 10 | ICR00093 | ICR07050 | 29 | BNL3261 | CGR5111 | 6.06 | 4.16 | 0.06 | -0.49 | 0.04 | 0.04 | -0.08 |
| BW | 8 | NAU4064 | CGR6508 | 8 | Gh197 | DC20094 | 5.43 | 2.24 | 0.90 | -0.05 | -0.01 | -0.03 | 0.04 |
|  | 2 | PGML0700 | SWU12016 | 9 | CGR6876 | CGR5758 | 6.54 | 4.00 | 0.12 | -0.05 | 0.00 | -0.01 | 0.01 |
|  | 8 | HAU3177 | NAU4064 | 9 | SWU15157 | SWU14934 | 5.35 | 2.82 | 0.59 | -0.05 | -0.03 | 0.00 | 0.02 |
|  | 7 | CER0036 | PGML1916 | 12 | HAU1316 | NAU3519 | 6.09 | 2.92 | 0.85 | 0.05 | -0.01 | 0.03 | -0.03 |
|  | 7 | SWU10785 | CER0036 | 14 | TMB0071 | BNL3661 | 5.27 | 2.90 | 0.13 | 0.05 | -0.01 | 0.01 | -0.01 |
|  | 7 | NAU1357 | SWU10067 | 16 | CGR6802 | HAU1129 | 5.54 | 3.47 | 0.07 | 0.05 | 0.01 | 0.00 | -0.01 |
|  | 16 | ICR00010 | SWU10038 | 19 | SWU17897 | CGR5539 | 7.24 | 3.59 | 1.09 | 0.05 | -0.03 | 0.04 | -0.01 |
|  | 2 | SWU12126 | SWU12147 | 20 | SWU20700 | CGR5548 | 5.47 | 3.65 | 0.00 | 0.05 | 0.00 | 0.00 | 0.00 |
|  | 16 | DPL0048 | SWU10266 | 21 | Gh451 | SWU16489 | 5.84 | 2.70 | 0.80 | -0.04 | 0.01 | -0.03 | 0.02 |
|  | 9 | SWU15157 | SWU14934 | 21 | SWU0830 | HAU2004 | 7.03 | 4.06 | 0.32 | 0.06 | -0.02 | 0.02 | 0.00 |
|  | 19 | NAU3437 | NAU2894 | 21 | **CGR5806** | **DPL0777** | 5.02 | 2.69 | 0.35 | 0.05 | 0.01 | 0.02 | -0.03 |
|  | 15 | DPL0182 | SWU11691 | 22 | PGML0695 | SWU20813 | 5.10 | 2.49 | 0.45 | 0.04 | 0.00 | 0.02 | -0.02 |
|  | 21 | SWU16649 | BNL1552 | 24 | SWU13758 | CGR5423 | 7.24 | 3.89 | 0.44 | -0.05 | 0.01 | -0.02 | 0.02 |
|  | 14 | TMB0071 | BNL3661 | 25 | HAU1382 | SWU19848 | 6.05 | 2.72 | 0.93 | -0.05 | -0.01 | -0.03 | 0.04 |
|  | 20 | DPL0319 | HAU1378 | 25 | BNL3594 | DPL0282 | 5.68 | 3.41 | 0.21 | -0.05 | -0.02 | 0.01 | 0.01 |
|  | 4 | ICR01729 | SWU16781 | 25 | SWU19430 | PGML1219 | 5.00 | 2.59 | 0.52 | -0.04 | 0.01 | -0.03 | 0.02 |
|  | 10 | BNL2960 | SWU20511 | 26 | BNL598 | PGML1637 | 5.61 | 2.74 | 0.78 | 0.05 | -0.03 | 0.03 | 0.01 |
|  | 11 | CAU0003 | DC40250 | 26 | BNL2495 | DPL0491 | 5.35 | 3.26 | 0.07 | -0.05 | 0.01 | -0.01 | 0.00 |
|  | 18 | CIR099 | NAU748 | 26 | BNL2495 | DPL0491 | 5.56 | 2.83 | 0.45 | 0.05 | 0.02 | 0.01 | -0.03 |
|  | 2 | PGML0700 | SWU12016 | 26 | Gh64 | SWU17257 | 5.75 | 3.01 | 0.43 | -0.05 | -0.01 | -0.02 | 0.02 |
|  | 23 | PGML4186 | NAU3100 | 26 | SWU17251 | C2_0135 | 5.31 | 2.98 | 0.22 | 0.05 | -0.02 | 0.01 | 0.01 |
|  | 13 | SHIN1462 | SWU22374 | 26 | C2_0135 | PGML2321 | 6.38 | 3.58 | 0.26 | 0.05 | 0.00 | -0.02 | 0.01 |
|  | 14 | PGML1568 | Gh529 | 28 | SHIN0219 | TMB2386 | 5.87 | 3.53 | 0.23 | 0.05 | 0.01 | 0.00 | -0.02 |
|  | 17 | SWU14627 | CGR5871 | 31 | SWU16676 | SWU16755 | 5.62 | 3.32 | 0.18 | -0.05 | 0.00 | -0.01 | 0.02 |
|  | 19 | NAU5330 | Gh72 | 32 | NAU2140 | NAU2957 | 7.68 | 4.96 | 0.06 | -0.06 | 0.00 | 0.00 | 0.01 |
|  | 26 | SWU18681 | SWU0598 | 32 | NAU2140 | NAU2957 | 5.22 | 2.99 | 0.19 | 0.05 | 0.00 | 0.01 | -0.02 |
| LP | 1 | NAU3384 | CGR5663 | 1 | CGR5663 | NAU2343 | 6.33 | 1.43 | 0.00 | -0.49 | -0.07 | -0.07 | 0.14 |
|  | 5 | HAU1603 | PGML4457 | 7 | CGR5001 | CGR6586 | 5.29 | 3.10 | 0.24 | 0.24 | -0.07 | 0.10 | -0.03 |
|  | 4 | SWU12672 | HAU1332 | 7 | SWU10205 | HAU1483a | 5.45 | 3.32 | 0.20 | -0.25 | 0.01 | 0.06 | -0.07 |
|  | 1 | ICR03724 | ICR03725 | 10 | ICR00093 | ICR07050 | 5.56 | 3.39 | 0.09 | 0.26 | -0.03 | 0.07 | -0.04 |
|  | 7 | Gh474 | SWU10785 | 13 | SWU22413 | CGR5331 | 5.90 | 3.65 | 0.08 | -0.26 | 0.05 | -0.04 | -0.01 |
|  | 13 | SWU13032 | DPL0308 | 14 | ICR12037 | CGR5675 | 6.10 | 3.38 | 0.20 | -0.27 | 0.08 | -0.06 | -0.02 |
|  | 13 | SWU22374 | HAU2857 | 15 | DPL0182 | SWU11691 | 5.02 | 3.18 | 0.03 | -0.25 | 0.01 | 0.00 | -0.01 |
|  | 16 | PGML1709 | SWU10627 | 16 | SWU20341 | DPL0897 | 6.27 | 3.35 | 0.74 | -0.27 | -0.04 | 0.17 | -0.14 |
|  | 5 | PGML1917 | SWU17715 | 16 | SWU10038 | ICR00016 | 5.26 | 3.13 | 0.03 | 0.25 | 0.01 | 0.03 | -0.03 |
|  | 3 | SWU12819 | SWU12765 | 18 | Gh60 | SWU22281 | 5.10 | 3.16 | 0.03 | 0.24 | 0.00 | 0.02 | -0.02 |
|  | 18 | Gh60 | SWU22281 | 18 | DC40150 | ICR02849 | 5.56 | 3.33 | 0.16 | -0.30 | 0.03 | -0.09 | 0.07 |
|  | 12 | NAU943 | DPL0303 | 20 | SWU20675 | SWU20649 | 6.39 | 4.12 | 0.03 | 0.28 | 0.04 | -0.01 | -0.03 |
|  | 2 | SWU12393 | SWU11013 | 20 | SWU20246 | SWU20501a | 5.16 | 2.85 | 0.40 | 0.23 | 0.00 | 0.11 | -0.11 |
|  | 3 | SWU12783 | SWU12819 | 21 | SWU16651 | SWU16645 | 5.26 | 3.32 | 0.03 | -0.25 | 0.01 | -0.02 | 0.01 |
|  | 12 | DPL0303 | COT107 | 21 | Gh451 | SWU16489 | 5.22 | 3.11 | 0.31 | 0.24 | 0.08 | 0.01 | -0.10 |
|  | 6 | ICR10602 | SWU19656 | 21 | SWU16487 | SWU16488 | 5.00 | 2.79 | 0.32 | -0.23 | 0.03 | -0.11 | 0.07 |
|  | 19 | SWU17882 | CAU0104 | 21 | CGR5217 | BNL3442a | 5.17 | 3.27 | 0.08 | -0.25 | -0.04 | -0.02 | 0.06 |
|  | 22 | PGML1712 | SWU21538 | 22 | CAU0161 | NAU2026 | 6.40 | 3.89 | 0.14 | 0.29 | -0.08 | 0.06 | 0.02 |
|  | 18 | Gh60 | SWU22281 | 24 | CGR5202 | Gh298 | 5.20 | 3.05 | 0.22 | 0.24 | 0.09 | -0.06 | -0.03 |
|  | 14 | PGML1568 | Gh529 | 25 | CGR6864 | SWU19815 | 5.72 | 2.85 | 0.56 | 0.23 | -0.01 | 0.13 | -0.12 |
|  | 8 | DC20094 | HAU1470b | 25 | Gh220 | SWU19434 | 5.03 | 2.73 | 0.47 | 0.23 | -0.13 | 0.10 | 0.03 |
|  | 24 | **HAU2504** | **SWU13736** | 26 | SWU17395 | DC30107 | 5.30 | 2.84 | 0.24 | -0.23 | 0.04 | -0.10 | 0.06 |
|  | 25 | BNL3594 | DPL0282 | 26 | SWU17395 | DC30107 | 6.76 | 4.07 | 0.05 | 0.28 | -0.02 | 0.03 | -0.01 |
|  | 16 | SWU10038 | ICR00016 | 26 | Gh64 | SWU17257 | 5.54 | 2.61 | 0.58 | 0.23 | 0.13 | -0.14 | 0.01 |
|  | 14 | SWU14224 | DPL0565 | 26 | SWU0514 | SWU18488 | 5.90 | 3.55 | 0.16 | -0.25 | -0.08 | 0.04 | 0.04 |
|  | 21 | CGR5217 | BNL3442a | 26 | SWU18488 | SWU18672 | 5.22 | 3.09 | 0.25 | -0.24 | 0.05 | 0.05 | -0.09 |
|  | 7 | NAU1357 | SWU10067 | 27 | CGR6857 | ICR11205 | 5.23 | 2.64 | 0.03 | 0.23 | -0.06 | 0.06 | 0.00 |
| BCVF_1_ population | | | | | | | | | | | | | |
| SY | 2 | SWU11889 | JESPR304 | 23 | **NAU2140** | **DC40286** | 5.85 | 0.66 | 3.91 | 0.94 | -1.10 | -2.01 | 3.10 |
|  | 32 | HAU1000 | TMB1931 | 36 | CGR5548 | SWU20700 | 6.09 | 1.79 | 2.30 | -1.54 | 1.15 | 1.30 | -2.45 |
| LY | 1 | PGML2498 | SWU14490 | 1 | DPL0790 | ICR03725 | 5.20 | 2.97 | 1.04 | 0.83 | -0.39 | -0.33 | 0.72 |
|  | 8 | HAU0810 | TMB2904 | 12 | DPL0400 | HAU2173 | 5.94 | 1.06 | 3.61 | -0.49 | 0.39 | 0.86 | -1.25 |
|  | 2 | TMB1268 | SWU11976 | 23 | **NAU2140** | **DC40286** | 5.08 | 0.94 | 3.33 | 0.48 | -0.63 | -0.59 | 1.22 |
|  | 25 | SWU19676 | NAU2968 | 25 | SWU19676 | NAU2968 | 5.10 | 1.26 | 0.12 | 0.77 | -0.51 | -0.48 | 0.98 |
|  | 26 | CGR6477 | PGML2562 | 30 | TMB1638 | CGR6812 | 5.27 | 1.62 | 2.29 | -0.60 | 0.73 | 0.23 | -0.96 |
|  | 1 | ICR03724 | SWU11632 | 34 | DPL0897 | SWU20341 | 5.40 | 2.11 | 1.76 | -0.69 | 0.67 | 0.18 | -0.85 |
|  | 32 | HAU1000 | TMB1931 | 36 | CGR5548 | SWU20700 | 5.67 | 1.55 | 2.61 | -0.60 | 0.44 | 0.64 | -1.08 |
| BNP | 1 | SWU10912 | HAU2489 | 1 | DPL0790 | ICR03725 | 5.21 | 0.88 | 1.52 | 0.28 | -0.49 | 0.49 | 0.00 |
|  | 14 | ICR03105 | ICR01124 | 21 | CGR5748 | PGML2500 | 5.97 | 0.31 | 3.26 | 0.17 | -0.70 | 0.58 | 0.12 |
| BW | 15 | NAU3736 | SWU11691 | 17 | SWU12838a | HAU1413 | 5.59 | 2.51 | 0.82 | -0.05 | 0.01 | 0.03 | -0.04 |
|  | 1 | SWU14616 | SWU14077 | 18 | SWU0738 | ICR02849 | 5.69 | 2.58 | 0.53 | 0.05 | -0.01 | -0.02 | 0.03 |
|  | 10 | HAU0635 | PGML4154 | 26 | **CGR6477** | **PGML2562** | 6.68 | 3.43 | 0.44 | 0.06 | 0.00 | -0.03 | 0.02 |
|  | 1 | ICR03295 | SWU10912 | 26 | **CGR6477** | **PGML2562** | 5.17 | 2.89 | 0.54 | 0.05 | 0.01 | -0.03 | 0.02 |
|  | 14 | HAU0883 | CIR228 | 31 | CGR6772 | HAU0355 | 6.24 | 3.22 | 0.82 | 0.05 | 0.00 | -0.03 | 0.03 |
|  | 1 | CGR6784 | NAU3393 | 37 | BNL5602 | JESPR251 | 6.61 | 4.32 | 0.25 | 0.06 | 0.01 | -0.02 | 0.01 |
| LP | 4 | BNL1167 | JESPR234 | 21 | SWU16361 | SWU16408 | 5.52 | 2.93 | 0.89 | 0.23 | 0.17 | -0.14 | -0.03 |
|  | 9 | CGR6876 | BNL1317 | 23 | BNL3482 | HAU0244 | 6.13 | 2.76 | 0.79 | -0.23 | -0.06 | -0.12 | 0.18 |
|  | 12 | HAU3373 | CGR6847 | 27 | Gh247 | CGR5867 | 5.12 | 2.80 | 0.87 | -0.22 | -0.16 | 0.03 | 0.13 |
|  | 24 | Gh54 | Gh454 | 27 | Gh247 | CGR5867 | 5.03 | 2.51 | 0.70 | 0.21 | -0.07 | 0.16 | -0.09 |

See footnotes of additional table S5 for explanations
