# Supplementary material for: Partial Dominance, Overdominance, Epistasis and QTL by Environment Interactions Contribute to Heterosis in Two Upland Cotton Hybrids
Source: G3 (Bethesda). 2015 Dec 29;6(3):499–507. doi: 10.1534/g3.115.025809 (PMC4777113; doi:10.1534/g3.115.025809)
Supplement: Supporting Information [file supp_g3.115.025809_TableS7.doc]

Table S7 Epistatic effects and environmental interactions detected for yield and yield components in two MPH data using two-locus analysis by inclusive composite interval mapping

| Trait | Chi | Flanking markers | | Chj | Flanking markers | | LOD | V(AA) | V(AAE) | AA | AAE1 | AAE2 | AAE3 |
| --- | --- | --- | --- | --- | --- | --- | --- | --- | --- | --- | --- | --- | --- |
| MPH (XZ hybrid) | | | | | | | | | | | | | |
| SY | 13 | DPL0894 | SWU10800 | 18 | SWU22281 | SWU21800 | 5.27 | 1.52 | 2.73 | -1.14 | 0.54 | 1.55 | -2.09 |
|  | 5 | SWU17787 | SWU13378 | 25 | SWU19411 | SWU19412 | 5.12 | 1.89 | 2.16 | -1.28 | 0.63 | 1.27 | -1.90 |
|  | 21 | CGR5806 | DPL0777 | 25 | SWU19411 | SWU19412 | 7.32 | 4.08 | 1.17 | 1.97 | -0.79 | -0.68 | 1.47 |
|  | 3 | Gh663 | CGR6528 | 26 | DPL0491 | Gh64 | 5.62 | 2.29 | 1.52 | -1.42 | 0.70 | 1.00 | -1.70 |
| LY | 13 | DPL0894 | SWU10800 | 18 | SWU22281 | SWU21800 | 5.12 | 1.38 | 2.74 | -0.44 | 0.26 | 0.59 | -0.85 |
|  | 21 | CGR5806 | DPL0777 | 25 | SWU19411 | SWU19412 | 8.05 | 4.68 | 1.03 | 0.85 | -0.29 | -0.27 | 0.56 |
|  | 3 | Gh663 | CGR6528 | 26 | DPL0491 | Gh64 | 5.63 | 2.43 | 1.41 | -0.59 | 0.22 | 0.43 | -0.65 |
|  | 18 | NAU748 | SWU22192 | 26 | SWU17233 | SWU17251 | 5.29 | 1.21 | 2.42 | 0.41 | 0.15 | -0.78 | 0.62 |
| BNP | 3 | SWU12732 | SWU12783 | 5 | CGR5025 | NBRI0694 | 5.70 | 3.78 | 0.66 | 0.45 | -0.03 | -0.23 | 0.26 |
|  | 19 | TMB0107 | NAU3217 | 24 | SWU13745 | Gh273 | 5.05 | 2.81 | 1.03 | 0.39 | -0.20 | -0.13 | 0.34 |
|  | 5 | SWU13378 | SWU17846 | 25 | DPL0282 | SWU19763 | 5.32 | 3.40 | 0.49 | -0.43 | 0.18 | 0.04 | -0.21 |
| BW | 7 | SWU10205 | HAU1483a | 13 | SWU22374 | HAU2857 | 5.33 | 2.94 | 0.86 | 0.05 | -0.03 | 0.00 | 0.03 |
|  | 16 | SWU10062 | SWU10094 | 16 | SWU10060 | SWU10054 | 5.03 | 0.05 | 0.00 | -0.02 | -0.01 | 0.11 | -0.11 |
| LP | 1 | SWU10986 | NAU2218 | 19 | SWU17782 | DPL0056 | 7.17 | 4.67 | 0.22 | 0.22 | 0.02 | 0.04 | -0.07 |
|  | 14 | CIR228 | BNL2485 | 28 | SHIN0219 | TMB2386 | 5.52 | 2.26 | 1.87 | -0.15 | -0.09 | -0.10 | 0.20 |
|  | 18 | DC40150 | ICR02849 | 29 | BNL3261 | CGR5111 | 5.51 | 3.30 | 0.80 | -0.20 | -0.02 | -0.11 | 0.12 |
|  | 27 | SWU10994 | HAU1001 | 30 | CER0168 | SWU21718 | 5.00 | 3.67 | 0.12 | 0.20 | 0.00 | 0.05 | -0.05 |
| MPH (XZV hybrid) | | | | | | | | | | | | | |
| SY | 1 | Gh529 | SWU17434 | 14 | HAU2482 | NAU4045 | 5.10 | 1.17 | 2.17 | 1.90 | -3.46 | 0.82 | 2.65 |
|  | 6 | HAU2768 | HAU0483 | 14 | HAU2482 | NAU4045 | 5.72 | 1.63 | 1.87 | -2.28 | 3.18 | -0.33 | -2.85 |
|  | 13 | SWU13032 | HAU2850 | 14 | NAU4045 | ICR03943 | 5.34 | 0.87 | 2.98 | -1.53 | 3.32 | 0.21 | -3.53 |
|  | 10 | NAU4967 | SWU19932 | 16 | NAU747 | HAU1129 | 5.12 | 0.64 | 2.51 | -1.16 | 2.97 | -0.33 | -2.64 |
|  | 13 | SWU13032 | HAU2850 | 19 | SWU17789 | SWU17882 | 5.98 | 0.33 | 3.99 | -0.84 | 3.37 | 0.39 | -3.76 |
|  | 7 | CGR5372 | C2_0046 | 21 | SHIN0337 | SWU16370 | 5.03 | 0.09 | 2.64 | -0.47 | 3.25 | -0.68 | -2.57 |
|  | 2 | SWU11976 | SWU12001 | 21 | **BNL3171** | **HAU2937** | 6.03 | 0.58 | 4.56 | -1.12 | 3.18 | 1.08 | -4.26 |
|  | 14 | HAU2482 | NAU4045 | 23 | DC40286 | PGML1434 | 5.59 | 2.17 | 1.82 | -2.82 | 2.56 | 0.39 | -2.95 |
|  | 14 | HAU2482 | NAU4045 | 24 | PGML1207 | Gh54 | 5.35 | 0.90 | 2.49 | 1.77 | -3.69 | -0.17 | 3.85 |
|  | 24 | Gh54 | Gh454 | 24 | CGR6079 | SWU13100 | 5.44 | 0.89 | 2.87 | -1.57 | 3.56 | 0.11 | -3.67 |
|  | 1 | Gh120 | Gh398 | 33 | BNL3661 | PGML4891 | 5.51 | 0.77 | 2.97 | -1.28 | 3.06 | 0.05 | -3.11 |
|  | 6 | MUSB1144 | BNL3650 | 34 | JESPR297 | ICR00647 | 5.22 | 1.87 | 2.16 | 2.02 | -2.10 | -0.86 | 2.97 |
|  | 25 | SWU19676 | NAU2968 | 36 | CER0167 | SWU20658 | 6.28 | 2.34 | 2.15 | -2.23 | 1.88 | 1.11 | -2.99 |
|  | 13 | CER0165 | SWU13032 | 39 | NAU5480 | DPL0270 | 6.60 | 1.36 | 5.26 | -1.69 | 3.10 | 1.51 | -4.62 |
| LY | 1 | PGML2498 | SWU14490 | 6 | MUSB1144 | BNL3650 | 5.33 | 0.73 | 3.29 | -0.51 | 1.01 | 0.48 | -1.49 |
|  | 3 | SWU12840 | NAU2742 | 13 | NAU3398 | CGR5331 | 5.19 | 0.30 | 2.14 | -0.34 | 1.25 | -0.46 | -0.79 |
|  | 3 | SWU12840 | NAU2742 | 14 | ICR03105 | ICR01124 | 5.15 | 1.29 | 2.53 | 0.83 | -1.23 | -0.35 | 1.58 |
|  | 2 | SWU11976 | SWU12001 | 21 | **BNL3171** | **HAU2937** | 6.26 | 0.51 | 4.59 | -0.43 | 1.29 | 0.46 | -1.75 |
|  | 14 | HAU2482 | NAU4045 | 23 | DC40286 | PGML1434 | 5.08 | 1.93 | 1.55 | -1.11 | 0.93 | 0.17 | -1.10 |
|  | 24 | Gh54 | Gh454 | 24 | HAU3076 | SWU13121 | 5.46 | 0.89 | 3.05 | -0.65 | 1.49 | 0.14 | -1.63 |
|  | 2 | SWU12490 | DPL0200 | 31 | SWU16730 | SWU16721 | 5.39 | 0.63 | 3.44 | 0.46 | -1.29 | -0.11 | 1.40 |
|  | 20 | Gh451 | HAU1314 | 34 | DPL0897 | SWU20341 | 5.21 | 1.12 | 2.71 | -0.63 | 1.12 | 0.15 | -1.27 |
|  | 12 | DPL0400 | HAU2173 | 39 | SWU16437 | SWU16432 | 5.18 | 1.35 | 2.69 | 0.69 | -0.87 | -0.49 | 1.36 |
| BNP | 1 | CGR6129 | ICR03295 | 2 | DPL0217 | CGR6695 | 5.14 | 2.26 | 0.45 | -0.44 | 0.27 | -0.17 | -0.10 |
|  | 5 | Gh260 | PGML0120 | 5 | Gh260 | PGML0120 | 5.22 | 2.70 | 0.96 | -0.51 | 0.12 | 0.30 | -0.42 |
|  | 2 | TMB1268 | SWU11976 | 6 | DC40417 | MUSB1164 | 6.44 | 3.57 | 0.57 | -0.55 | 0.26 | 0.02 | -0.28 |
|  | 6 | SWU19656 | CGR5124 | 6 | CGR5124 | SWU19249 | 5.23 | 0.81 | 0.00 | -0.73 | 0.62 | -0.33 | -0.29 |
|  | 6 | MUSB1144 | BNL3650 | 8 | DC20094 | Gh197 | 5.13 | 2.49 | 0.85 | 0.46 | -0.34 | 0.01 | 0.33 |
|  | 1 | Gh398 | CGR6129 | 8 | HAU0810 | TMB2904 | 5.28 | 1.80 | 1.23 | 0.39 | -0.43 | 0.08 | 0.35 |
|  | 9 | DC30015 | SWU15413 | 12 | ICR03107 | HAU3373 | 5.16 | 1.76 | 0.79 | -0.39 | 0.30 | -0.34 | 0.04 |
|  | 6 | CGR6749 | NAU3186 | 12 | HAU3373 | CGR6847 | 6.32 | 2.55 | 1.90 | -0.46 | 0.29 | 0.28 | -0.57 |
|  | 8 | DC20094 | Gh197 | 14 | ICR03943 | ICR12281 | 5.79 | 2.74 | 0.52 | 0.51 | -0.30 | 0.06 | 0.24 |
|  | 14 | HAU0883 | CIR228 | 17 | HAU1413 | CGR5576 | 5.51 | 2.77 | 0.57 | -0.49 | 0.30 | -0.20 | -0.10 |
|  | 13 | CER0165 | SWU13032 | 18 | SWU0738 | ICR02849 | 5.83 | 2.95 | 0.52 | -0.55 | 0.33 | -0.14 | -0.19 |
|  | 1 | NAU0748 | NAU2697 | 21 | CGR5748 | PGML2500 | 5.70 | 2.23 | 1.07 | -0.44 | 0.43 | -0.23 | -0.20 |
|  | 15 | NAU3736 | SWU11691 | 21 | CGR6521 | Gh450 | 5.63 | 2.89 | 0.49 | 0.50 | -0.05 | -0.23 | 0.27 |
|  | 1 | SWU11632 | SWU21958 | 23 | Gh327 | ICR06429 | 5.24 | 1.28 | 1.67 | -0.36 | 0.46 | 0.09 | -0.55 |
|  | 24 | Gh54 | Gh454 | 24 | CGR6079 | SWU13100 | 6.35 | 2.81 | 0.85 | -0.54 | 0.47 | -0.05 | -0.41 |
|  | 6 | BNL3650 | TMB2940 | 25 | NAU2968 | DPL0377 | 5.25 | 3.00 | 0.32 | 0.50 | -0.16 | -0.06 | 0.22 |
|  | 5 | Gh260 | PGML0120 | 25 | SWU19434 | SWU19412 | 7.36 | 3.38 | 1.01 | 0.56 | -0.41 | 0.25 | 0.17 |
|  | 12 | HAU3373 | CGR6847 | 26 | CGR6477 | PGML2562 | 5.82 | 2.53 | 1.01 | -0.47 | 0.35 | 0.03 | -0.38 |
|  | 13 | NAU3398 | CGR5331 | 31 | CGR6772 | HAU0355 | 5.82 | 2.96 | 0.56 | -0.50 | 0.24 | -0.30 | 0.05 |
|  | 9 | CGR6876 | BNL1317 | 31 | **SWU16730** | **SWU16721** | 5.09 | 2.83 | 0.28 | -0.49 | 0.22 | -0.12 | -0.10 |
|  | 6 | MUSB1144 | BNL3650 | 34 | JESPR297 | ICR00647 | 6.78 | 2.48 | 1.37 | 0.47 | -0.48 | 0.15 | 0.33 |
|  | 25 | SWU19676 | NAU2968 | 36 | CER0167 | SWU20658 | 5.24 | 2.08 | 1.31 | -0.42 | 0.35 | 0.11 | -0.46 |
|  | 24 | PGML1207 | Gh54 | 39 | HAU2022 | BNL0827 | 5.73 | 2.64 | 0.99 | 0.48 | -0.25 | -0.17 | 0.42 |
|  | 13 | CER0165 | SWU13032 | 39 | NAU5480 | DPL0270 | 6.99 | 2.85 | 2.71 | -0.49 | 0.25 | 0.42 | -0.67 |
| BW | 6 | SWU19184 | DPL0847 | 16 | HAU3081 | NAU747 | 6.36 | 3.55 | 1.22 | 0.06 | -0.03 | -0.02 | 0.05 |
|  | 13 | CGR5331 | SHIN1462 | 23 | SHIN1076 | BNL3482 | 5.37 | 3.42 | 0.25 | -0.06 | 0.02 | 0.01 | -0.02 |
|  | 10 | NAU3395 | CAU0234 | 34 | DPL0897 | SWU20341 | 6.86 | 3.33 | 1.23 | -0.06 | 0.04 | 0.01 | -0.05 |
|  | 2 | CGR6695 | SWU11013 | 36 | CER0167 | SWU20658 | 7.97 | 2.12 | 3.60 | 0.05 | -0.07 | -0.02 | 0.08 |
| LP | 18 | SWU0738 | ICR02849 | 29 | DPL0171 | Gh499 | 5.89 | 2.01 | 2.24 | -0.17 | -0.23 | 0.03 | 0.20 |

See footnotes of additional table S5 for explanations
