# Supplementary material for: Partial Dominance, Overdominance, Epistasis and QTL by Environment Interactions Contribute to Heterosis in Two Upland Cotton Hybrids
Source: G3 (Bethesda). 2015 Dec 29;6(3):499–507. doi: 10.1534/g3.115.025809 (PMC4777113; doi:10.1534/g3.115.025809)
Supplement: Supporting Information [file supp_g3.115.025809_TableS8.doc]

**Table S8** Same QTLs for yield and yield component traits compared with results of F_2:3_ and F_2:4_ populations

| Trait | QTL | Population | Env. | Chr | Flanking Markers | | LOD | A | D | Var% |
| --- | --- | --- | --- | --- | --- | --- | --- | --- | --- | --- |
| SY | ***qSY-Chr21-1*** | RIL' | E3 | 21 | SWU15915 | SWU0189 | 4.27 | -5.42 |  | 7.77 |
|  |  | RIL | E3 | 21 | SWU15915 | SWU0189 | 3.39 | -4.51 |  | 6.11 |
|  |  | F_2:4_ | E1 | 11 | CGR5602 | DPL050a | 10.15 | 1.95 | 1.95 | 23.13 |
|  | ***qSY-Chr26-2*** | BC | E3 | 26 | NAU5072 | BNL2495 | 2.36 | 6.85 |  | 4.43 |
|  |  | RIL' | E3 | 26 | SWU17336 | NAU5072 | 2.38 | 4.24 |  | 4.80 |
|  |  | RIL | E3 | 26 | SWU17336 | NAU5072 | 4.43 | 5.64 |  | 9.62 |
|  |  | F_2:3_ | E1 | 26 | CGR6880 | CGR5452 | 2.35 | -4.35 | -1.90 | 5.24 |
|  |  | F_2:3_ | E2 | 26 | CGR6880 | CGR5452 | 3.39 | -8.74 | 0.67 | 7.59 |
| LY | ***qLY-Chr21-1*** | RIL' | E3 | 21 | SWU15915 | SWU0189 | 4.01 | -2.07 |  | 7.00 |
|  |  | RIL | E3 | 21 | SWU15915 | SWU0189 | 5.28 | -3.56 |  | 9.41 |
|  |  | F_2:4_ | E1 | 11 | CGR5602 | DPL050a | 7.49 | 0.60 | 0.76 | 16.21 |
|  | ***qLY-Chr26-3*** | BC | E3 | 26 | NAU5072 | BNL2495 | 2.46 | 2.83 |  | 4.61 |
|  |  | RIL' | E3 | 26 | SWU17336 | NAU5072 | 3.25 | 2.03 |  | 6.59 |
|  |  | RIL | E3 | 26 | SWU17336 | NAU5072 | 3.45 | 1.97 |  | 6.91 |
|  |  | F_2:3_ | E1 | 26 | CGR6880 | CGR5452 | 3.18 | -1.58 | -1.55 | 6.80 |
|  |  | F_2:3_ | E2 | 26 | CGR6880 | CGR5452 | 5.79 | -4.72 | 0.36 | 12.64 |
| BNP | ***qBNP-Chr21-1*** | RIL' | E1 | 21 | CGR5808 | HAU0423 | 3.21 | -0.68 |  | 6.27 |
|  |  | RIL | E1 | 21 | HAU0423 | CGR5806 | 2.1 | -0.65 |  | 3.98 |
|  |  | F_2:4_ | E2 | 11 | CGR5808 | CGR5217 | 3.03 | 1.46 | -1.16 | 8.79 |
|  | ***qBNP-Chr24-2*** | RIL' | E2 | 24 | SWU13758 | CGR5423 | 2.46 | 0.89 |  | 9.61 |
|  |  | RIL | E2 | 24 | SWU13758 | CGR5423 | 4.57 | 0.87 |  | 8.97 |
|  |  | F_2:3_ | E2 | 24 | DPL551 | GH273 | 3.27 | -1.06 | -0.21 | 8.37 |
|  |  | F_2:3_ | E1 | 24 | CGR5423 | SHIN1076 | 4.39 | -0.64 | -0.55 | 9.78 |
| BW | ***qBW-Chr4-1*** | BC | E1 | 4 | BNL1167 | SWU21415 | 3.33 | 0.14 |  | 6.92 |
|  |  | F_2:4_ | E1 | 4 | BNL3990 | BNL0530 | 2.86 | -0.17 | 0.09 | 5.30 |
|  |  | F_2:4_ | E2 | 4 | BNL3990 | BNL0530 | 3.27 | -0.23 | 0.14 | 7.23 |
|  | ***qBW-Chr5-1*** | RIL' | E2 | 5 | SWU20913 | Gh260 | 3.53 | 0.13 |  | 8.94 |
|  |  | RIL | E3 | 5 | PGML0120 | SWU20914 | 5.94 | 0.15 |  | 11.48 |
|  |  | F_2:3_ | E1 | 5 | BNL3447 | GH260 | 2.40 | -0.24 | 0.17 | 7.54 |
|  |  | F_2:3_ | E2 | 5 | BNL3447 | GH260 | 2.50 | -0.18 | -0.01 | 8.14 |
|  | ***qBW-Chr5-2*** | BC | E3 | 5 | SWU20917 | NAU6240 | 3.50 | 0.16 |  | 9.34 |
|  |  | RIL' | E3 | 5 | SWU20917 | NAU6240 | 6.81 | 0.21 |  | 18.19 |
|  |  | RIL | E3 | 5 | SWU20917 | NAU6240 | 6.12 | 0.17 |  | 16.02 |
|  |  | RIL | E2 | 5 | SWU20917 | NAU6240 | 6.28 | 0.17 |  | 15.73 |
|  |  | F_2:3_ | E1 | 5 | BNL3447 | GH260 | 2.40 | -0.24 | 0.17 | 7.54 |
|  |  | F_2:3_ | E2 | 5 | BNL3447 | GH260 | 2.50 | -0.18 | -0.01 | 8.14 |
|  | ***qBW-Chr5-5*** | MPH | E1 | 5 | TMB1296 | HAU1603 | 2.52 | -0.12 |  | 5.54 |
|  |  | RIL | E1 | 5 | NAU4034 | SWU17713 | 3.55 | 0.13 |  | 7.03 |
|  |  | F_2:3_ | E2 | 5 | NAU2865 | GH388 | 4.61 | -0.25 | -0.09 | 9.69 |
|  |  | F_2:3_ | E1 | 5 | HAU1603 | TMB1296 | 2.70 | -0.23 | 0.07 | 5.94 |
| LP | ***qLP-Chr2-1*** | RIL' | E2 | 2 | SWU12025 | SWU11889 | 2.13 | 0.62 |  | 7.93 |
|  |  | RIL | E3 | 2 | SWU11887 | SWU11976 | 3.00 | 0.50 |  | 4.75 |
|  |  | RIL | E3 | 2 | SWU11889 | SWU11887 | 3.87 | 0.64 |  | 6.93 |
|  |  | F_2:4_ | E2 | 2 | CGR6695 | DPL217 | 3.14 | -0.91 | 0.82 | 7.54 |
|  | ***qLP-Chr5-1*** | BC | E1 | 5 | SWU20913 | Gh260 | 6.05 | -1.05 |  | 14.26 |
|  |  | BC | E3 | 5 | SWU20913 | Gh260 | 6.36 | -0.89 |  | 13.67 |
|  |  | RIL' | E1 | 5 | SWU20913 | Gh260 | 7.96 | -0.88 |  | 16.29 |
|  |  | RIL' | E2 | 5 | SWU20913 | Gh260 | 9.39 | -1.03 |  | 21.98 |
|  |  | RIL' | E3 | 5 | PGML0120 | SWU20914 | 12.83 | -1.13 |  | 23.26 |
|  |  | RIL | E3 | 5 | SWU20913 | Gh260 | 8.03 | -1.07 |  | 19.78 |
|  |  | RIL | E1 | 5 | SWU20913 | Gh260 | 11.60 | -1.11 |  | 23.21 |
|  |  | RIL | E2 | 5 | SWU20913 | Gh260 | 10.93 | -0.94 |  | 21.62 |
|  |  | F_2:3_ | E1 | 5 | GH260 | NAU6240 | 3.76 | 0.95 | -0.77 | 9.33 |
|  |  | F_2:3_ | E2 | 5 | GH260 | NAU6240 | 6.10 | 1.34 | -0.75 | 16.70 |
|  |  | F_2:4_ | E2 | 5 | GH260 | NAU6240 | 6.68 | 1.20 | -0.56 | 16.18 |
|  | ***qLP-Chr5-2*** | BC | E1 | 5 | SWU20917 | NAU6240 | 6.10 | -1.09 |  | 15.51 |
|  |  | BC | E2 | 5 | SWU20917 | NAU6240 | 6.26 | -1.24 |  | 16.93 |
|  |  | RIL' | E1 | 5 | SWU20917 | NAU6240 | 8.06 | -0.93 |  | 18.10 |
|  |  | RIL' | E2 | 5 | SWU20917 | NAU6240 | 9.03 | -1.05 |  | 22.75 |
|  |  | RIL' | E3 | 5 | SWU20917 | NAU6240 | 14.82 | -1.41 |  | 37.72 |
|  |  | MPH | E3 | 5 | SWU20917 | NAU6240 | 3.10 |  | 0.54 | 7.59 |
|  |  | RIL | E3 | 5 | SWU20917 | NAU6240 | 10.03 | -1.24 |  | 26.42 |
|  |  | F_2:3_ | E1 | 5 | GH260 | NAU6240 | 3.76 | 0.95 | -0.77 | 9.33 |
|  |  | F_2:3_ | E2 | 5 | GH260 | NAU6240 | 6.10 | 1.34 | -0.75 | 16.70 |
|  |  | F_2:4_ | E2 | 5 | GH260 | NAU6240 | 6.68 | 1.20 | -0.56 | 16.18 |
|  | ***qLP-Chr13-3*** | BC | E3 | 13 | BNL1495 | CGR5390 | 2.91 | -0.65 |  | 7.12 |
|  |  | BC | E1 | 13 | DPL0894 | SWU10800 | 2.03 | -0.69 |  | 5.58 |
|  |  | RIL' | E3 | 13 | BNL1495 | CGR5390 | 5.38 | -0.77 |  | 11.10 |
|  |  | RIL' | E1 | 13 | DPL0894 | SWU10800 | 2.35 | -0.66 |  | 9.29 |
|  |  | RIL | E3 | 13 | BNL1495 | CGR5390 | 4.10 | -0.73 |  | 9.13 |
|  |  | F_2:3_ | E2 | 13 | DPL687 | DPL286 | 3.27 | 0.56 | 0.47 | 7.65 |

Env. : Environment, E1, Handan; E2: Cangzhou; E3: Xiangyang; E4: Hainan

Effect, the genetic expectation of a QTL effect obtained is the additive effect (A) when estimated from the RILs and RIL′s, the additive and dominance effects (A+D) from the BCF_1_ mean values, and the dominance effect (D) from the MPH values

Var%, Phenotypic variation explained by a single QTL
