# Supplementary material for: Partial Dominance, Overdominance, Epistasis and QTL by Environment Interactions Contribute to Heterosis in Two Upland Cotton Hybrids
Source: G3 (Bethesda). 2015 Dec 29;6(3):499–507. doi: 10.1534/g3.115.025809 (PMC4777113; doi:10.1534/g3.115.025809)
Supplement: Supporting Information [file supp_g3.115.025809_TableS1.doc]

**Table S1 QTLs identified for yield and yield components of RIL(V)′, RIL(V), BC(V)F_1_ and MPH data by composite interval mapping in two hybrids**

| Trait | QTL | Env | Marker interval | | RIL(V)**′** | | |  | RIL(V) | | |  | BC(V)F_1_ | | |  |  | MPH |  |
| --- | --- | --- | --- | --- | --- | --- | --- | --- | --- | --- | --- | --- | --- | --- | --- | --- | --- | --- | --- |
|  |  |  |  |  | LOD | A | Var% |  | LOD | A | Var% |  | LOD | A+D | Var% |  | LOD | D | Var% |
| XZ hybrid | | | | | | | | | | | | | | | | | | | |
| SY | ***qSY-Chr1-1*** | E2 | NAU3177 | ICR03724 | 4.57 | -3.72 | 8.67 |  |  |  |  |  |  |  |  |  |  |  |  |
|  |  | E1 | ICR03725 | SWU10987 | 2.18 | -1.97 | 4.31 |  |  |  |  |  |  |  |  |  |  |  |  |
|  |  | E2 | ICR03724 | ICR03725 |  |  |  |  | 3.96 | -3.48 | 8.66 |  |  |  |  |  |  |  |  |
|  |  | E3 | ICR03724 | ICR03725 |  |  |  |  | 2.30 | -3.50 | 3.87 |  |  |  |  |  |  |  |  |
|  | *qSY-Chr1-2* | E2 | SWU10986 | NAU2218 |  |  |  |  | 3.69 | -3.37 | 7.47 |  |  |  |  |  |  |  |  |
|  | ***qSY-Chr1-3*** | E2 | HAU1417 | NAU2437 |  |  |  |  |  |  |  |  | 2.22 | -5.14 | 7.11 |  |  |  |  |
|  |  | E3 | SWU0077 | HAU1417 |  |  |  |  |  |  |  |  |  |  |  |  | 2.25 | 4.93 | 4.78 |
|  |  | E2 | HAU1417 | NAU2437 |  |  |  |  |  |  |  |  |  |  |  |  | 3.54 | -7.11 | 15.05 |
|  | ***qSY-Chr1-4*** | E3 | ICR01320 | DPL0003 |  |  |  |  |  |  |  |  | 4.14 | -8.33 | 8.46 |  |  |  |  |
|  |  | E3 | SWU11038 | SWU11384 |  |  |  |  | 2.32 | -3.66 | 4.22 |  |  |  |  |  |  |  |  |
|  | ***qSY-Chr1-5*** | E1 | CGR6356 | SWU11632 |  |  |  |  |  |  |  |  |  |  |  |  | 2.22 | -3.42 | 5.00 |
|  |  | E1 | CGR6356 | SWU11632 |  |  |  |  | 2.44 | 2.64 | 5.08 |  |  |  |  |  |  |  |  |
|  | ***qSY-Chr2-1*** | E3 | SWU11887 | SWU11976 |  |  |  |  |  |  |  |  | 2.90 | 7.22 | 6.48 |  |  |  |  |
|  |  | E3 | SWU12025 | SWU11889 | 7.71 | 8.57 | 20.48 |  |  |  |  |  |  |  |  |  |  |  |  |
|  |  | E3 | SWU12025 | SWU11889 |  |  |  |  | 7.14 | 8.05 | 20.61 |  |  |  |  |  |  |  |  |
|  | *qSY-Chr2-2* | E3 | SWU11887 | SWU11976 | 8.38 | 8.72 | 21.29 |  |  |  |  |  |  |  |  |  |  |  |  |
|  | *qSY-Chr2-3* | E1 | MGHES24 | ICR11064 |  |  |  |  |  |  |  |  | 3.42 | 3.88 | 7.06 |  |  |  |  |
|  | ***qSY-Chr5-1*** | E3 | NAU6240 | PGML1671 |  |  |  |  |  |  |  |  | 3.35 | 8.10 | 8.41 |  |  |  |  |
|  |  | E2 | NAU6240 | PGML1671 | 4.57 | 3.82 | 9.59 |  |  |  |  |  |  |  |  |  |  |  |  |
|  | *qSY-Chr17-1* | E2 | SWU14627 | CGR5871 | 3.67 | 3.79 | 9.33 |  |  |  |  |  |  |  |  |  |  |  |  |
|  | *qSY-Chr19-1* | E1 | NAU3217 | SWU18015 |  |  |  |  |  |  |  |  | 3.34 | -3.92 | 6.96 |  |  |  |  |
|  | ***qSY-Chr20-1*** | E1 | CER0167 | SWU20064 |  |  |  |  |  |  |  |  | 3.78 | -5.07 | 11.92 |  |  |  |  |
|  |  | E2 | CER0167 | SWU20064 | 3.60 | -4.31 | 12.26 |  |  |  |  |  |  |  |  |  |  |  |  |
|  | ***qSY-Chr20-2*** | E1 | SWU1259 | SWU20033 |  |  |  |  |  |  |  |  | 3.32 | -3.87 | 6.97 |  |  |  |  |
|  |  | E2 | SWU1259 | SWU20033 |  |  |  |  |  |  |  |  | 3.41 | -5.24 | 7.63 |  |  |  |  |
|  | ***qSY-Chr21-1*** | E3 | SWU15915 | SWU0189 | 4.27 | -5.42 | 7.77 |  |  |  |  |  |  |  |  |  |  |  |  |
|  |  | E3 | SWU15915 | SWU0189 |  |  |  |  | 3.39 | -4.51 | 6.11 |  |  |  |  |  |  |  |  |
|  | ***qSY-Chr21-2*** | E1 | CGR5808 | HAU0423 | 4.85 | -2.95 | 9.94 |  |  |  |  |  |  |  |  |  |  |  |  |
|  |  | E2 | CGR5808 | HAU0423 |  |  |  |  | 3.94 | -3.59 | 9.07 |  |  |  |  |  |  |  |  |
|  | *qSY-Chr22-1* | E3 | BNL4030 | PGML0695 | 3.82 | -5.24 | 6.13 |  |  |  |  |  |  |  |  |  |  |  |  |
|  | ***qSY-Chr26-1*** | E3 | NAU2175 | SWU17336 | 3.85 | 5.19 | 7.26 |  |  |  |  |  |  |  |  |  |  |  |  |
|  |  | E3 | NAU2175 | SWU17336 |  |  |  |  | 5.22 | 5.80 | 10.24 |  |  |  |  |  |  |  |  |
|  | ***qSY-Chr26-2*** | E3 | NAU5072 | BNL2495 |  |  |  |  |  |  |  |  | 2.36 | 6.85 | 4.43 |  |  |  |  |
|  |  | E3 | SWU17336 | NAU5072 | 2.38 | 4.24 | 4.80 |  |  |  |  |  |  |  |  |  |  |  |  |
|  |  | E3 | SWU17336 | NAU5072 |  |  |  |  | 4.43 | 5.64 | 9.62 |  |  |  |  |  |  |  |  |
|  | *qSY-Chr26-3* | E3 | SWU17233 | SWU17251 |  |  |  |  |  |  |  |  |  |  |  |  | 3.50 | -6.16 | 7.36 |
|  | *qSY-Chr26-4* | E1 | PGML1289 | SWU18919 |  |  |  |  | 3.30 | 3.12 | 6.97 |  |  |  |  |  |  |  |  |
| LY | ***qLY-Chr1-1*** | E3 | CGR6129 | DPL0790 | 4.10 | -2.26 | 8.43 |  |  |  |  |  |  |  |  |  |  |  |  |
|  |  | E3 | ICR03295 | CGR6129 |  |  |  |  | 2.43 | -1.45 | 3.81 |  |  |  |  |  |  |  |  |
|  | ***qLY-Chr1-2*** | E2 | NAU3177 | ICR03724 | 4.43 | -1.47 | 9.11 |  |  |  |  |  |  |  |  |  |  |  |  |
|  |  | E3 | NAU3177 | ICR03724 | 3.27 | -2.00 | 6.58 |  |  |  |  |  |  |  |  |  |  |  |  |
|  |  | E2 | ICR03724 | ICR03725 |  |  |  |  | 2.95 | -1.19 | 6.57 |  |  |  |  |  |  |  |  |
|  |  | E3 | ICR03724 | ICR03725 |  |  |  |  | 2.28 | -1.44 | 3.70 |  |  |  |  |  |  |  |  |
|  | ***qLY-Chr1-3*** | E3 | ICR03725 | SWU10987 |  |  |  |  |  |  |  |  | 2.44 | -2.67 | 5.51 |  |  |  |  |
|  |  | E2 | ICR03725 | SWU10987 | 2.89 | -1.29 | 6.92 |  |  |  |  |  |  |  |  |  |  |  |  |
|  |  | E2 | SWU10986 | NAU2218 |  |  |  |  | 3.79 | -1.44 | 7.96 |  |  |  |  |  |  |  |  |
|  |  | E3 | SWU10986 | NAU2218 |  |  |  |  | 1.69 | -1.38 | 2.69 |  |  |  |  |  |  |  |  |
|  | *qLY-Chr1-4* | E2 | HAU1417 | NAU2437 |  |  |  |  |  |  |  |  |  |  |  |  | 3.04 | -2.65 | 13.05 |
|  | ***qLY-Chr1-5*** | E3 | SWU11560 | ICR01320 |  |  |  |  |  |  |  |  | 5.19 | -3.82 | 10.73 |  |  |  |  |
|  |  | E3 | ICR01320 | DPL0003 |  |  |  |  |  |  |  |  |  |  |  |  | 2.91 | -2.29 | 6.14 |
|  |  | E3 | SWU11384 | ICR11885 |  |  |  |  | 2.73 | -1.62 | 4.62 |  |  |  |  |  |  |  |  |
|  | ***qLY-Chr1-6*** | E3 | SWU11632 | CGR6857 |  |  |  |  |  |  |  |  | 3.56 | -3.12 | 7.51 |  |  |  |  |
|  |  | E1 | CGR6356 | SWU11632 |  |  |  |  |  |  |  |  |  |  |  |  | 2.51 | -1.47 | 5.47 |
|  |  | E2 | CGR6356 | SWU11632 |  |  |  |  | 2.69 | 1.06 | 5.35 |  |  |  |  |  |  |  |  |
|  | ***qLY-Chr2-1*** | E3 | SWU11887 | SWU11976 |  |  |  |  |  |  |  |  | 3.45 | 3.26 | 8.04 |  |  |  |  |
|  |  | E3 | SWU12025 | SWU11889 | 8.48 | 3.29 | 18.50 |  |  |  |  |  |  |  |  |  |  |  |  |
|  |  | E1 | SWU11889 | SWU11887 |  |  |  |  | 5.56 | 1.68 | 12.28 |  |  |  |  |  |  |  |  |
|  |  | E3 | SWU11889 | SWU11887 |  |  |  |  | 10.33 | 3.29 | 19.45 |  |  |  |  |  |  |  |  |
|  | *qLY-Chr2-2* | E3 | SWU11887 | SWU11976 | 8.61 | 3.57 | 21.91 |  |  |  |  |  |  |  |  |  |  |  |  |
|  | *qLY-Chr2-3* | E1 | NBRI0014 | SWU12107 |  |  |  |  |  |  |  |  | 3.15 | 1.69 | 7.59 |  |  |  |  |
|  | ***qLY-Chr16-1*** | E1 | ICR00647 | SWU20341 |  |  |  |  |  |  |  |  | 2.30 | -1.32 | 4.70 |  |  |  |  |
|  |  | E1 | ICR00647 | SWU20341 |  |  |  |  |  |  |  |  |  |  |  |  | 2.24 | -1.38 | 5.01 |
|  | ***qLY-Chr17-1*** | E3 | CGR5871 | SWU12876 | 2.17 | 2.41 | 10.02 |  |  |  |  |  |  |  |  |  |  |  |  |
|  |  | E1 | CGR5871 | SWU12876 |  |  |  |  | 2.03 | 1.58 | 11.77 |  |  |  |  |  |  |  |  |
|  | ***qLY-Chr21-1*** | E3 | SWU15915 | SWU0189 | 4.01 | -2.07 | 7.00 |  |  |  |  |  |  |  |  |  |  |  |  |
|  |  | E3 | SWU15915 | SWU0189 |  |  |  |  | 5.28 | -3.56 | 9.41 |  |  |  |  |  |  |  |  |
|  | *qLY-Chr21-2* | E1 | CGR5808 | HAU0423 | 3.15 | -1.00 | 7.61 |  |  |  |  |  |  |  |  |  |  |  |  |
|  | *qLY-Chr22-1* | E3 | BNL4030 | PGML0695 | 3.32 | -2.03 | 5.65 |  |  |  |  |  |  |  |  |  |  |  |  |
|  | ***qLY-Chr26-1*** | E3 | SWU17395 | DC30107 |  |  |  |  |  |  |  |  | 2.16 | 2.57 | 4.16 |  |  |  |  |
|  |  | E3 | DPL0070 | NAU2175 | 5.26 | 2.39 | 9.12 |  |  |  |  |  |  |  |  |  |  |  |  |
|  | ***qLY-Chr26-2*** | E3 | NAU2175 | SWU17336 |  |  |  |  |  |  |  |  | 2.16 | 2.68 | 4.46 |  |  |  |  |
|  |  | E3 | NAU2175 | SWU17336 |  |  |  |  | 4.57 | 2.17 | 8.41 |  |  |  |  |  |  |  |  |
|  | ***qLY-Chr26-3*** | E3 | NAU5072 | BNL2495 |  |  |  |  |  |  |  |  | 2.46 | 2.83 | 4.61 |  |  |  |  |
|  |  | E3 | SWU17336 | NAU5072 | 3.25 | 2.03 | 6.59 |  |  |  |  |  |  |  |  |  |  |  |  |
|  |  | E3 | SWU17336 | NAU5072 |  |  |  |  | 3.45 | 1.97 | 6.91 |  |  |  |  |  |  |  |  |
|  | *qLY-Chr26-4* | E3 | CGR6930 | SWU17241 |  |  |  |  |  |  |  |  |  |  |  |  | 3.40 | -2.39 | 7.21 |
|  | ***qLY-Chr26-5*** | E3 | C2_0135 | PGML2321 |  |  |  |  |  |  |  |  | 2.00 | 2.40 | 4.00 |  |  |  |  |
|  |  | E2 | C2_0135 | PGML2321 |  |  |  |  |  |  |  |  | 2.08 | -1.71 | 4.71 |  |  |  |  |
| BNP | *qBNP-Chr1-1* | E2 | SWU10986 | NAU2218 |  |  |  |  | 3.25 | -0.80 | 7.31 |  |  |  |  |  |  |  |  |
|  | *qBNP-Chr1-2* | E1 | SWU10994 | HAU1001 |  |  |  |  | 3.02 | -0.79 | 6.27 |  |  |  |  |  |  |  |  |
|  | ***qBNP-Chr1-3*** | E3 | ICR11883 | CGR6356 |  |  |  |  |  |  |  |  | 2.87 | -1.37 | 6.32 |  |  |  |  |
|  |  | E1 | CGR6356 | SWU11632 |  |  |  |  |  |  |  |  | 2.80 | -1.06 | 6.03 |  |  |  |  |
|  | ***qBNP-Chr11-1*** | E2 | CER0098 | CGR5421 |  |  |  |  |  |  |  |  | 2.18 | -1.21 | 6.45 |  |  |  |  |
|  |  | E3 | CGR5421 | ICR08245 |  |  |  |  |  |  |  |  | 2.74 | -1.52 | 7.84 |  |  |  |  |
|  |  | E2 | CER0098 | CGR5421 |  |  |  |  |  |  |  |  |  |  |  |  | 2.53 | -1.41 | 10.19 |
|  |  | E3 | CER0098 | CGR5421 |  |  |  |  |  |  |  |  |  |  |  |  | 2.51 | -1.45 | 7.70 |
|  | ***qBNP-Chr11-2*** | E2 | SWU15972 | TMB0628 |  |  |  |  |  |  |  |  |  |  |  |  | 3.12 | -1.49 | 11.44 |
|  |  | E3 | SWU15972 | TMB0628 |  |  |  |  | 2.52 | -1.04 | 10.06 |  |  |  |  |  |  |  |  |
|  | ***qBNP-Chr12-1*** | E1 | Gh631 | HAU1321 |  |  |  |  |  |  |  |  | 2.50 | -1.34 | 9.92 |  |  |  |  |
|  |  | E1 | Gh631 | HAU1321 | 2.48 | -0.79 | 8.48 |  |  |  |  |  |  |  |  |  |  |  |  |
|  |  | E2 | Gh631 | HAU1321 | 2.61 | -0.76 | 6.90 |  |  |  |  |  |  |  |  |  |  |  |  |
|  |  | E2 | HAU1316 | NAU3519 |  |  |  |  | 4.02 | -0.97 | 10.81 |  |  |  |  |  |  |  |  |
|  | *qBNP-Chr14-1* | E1 | HAU1057 | PGML0989 | 4.54 | 0.82 | 9.00 |  |  |  |  |  |  |  |  |  |  |  |  |
|  | *qBNP-Chr14-2* | E1 | Gh120 | PGML1884 |  |  |  |  |  |  |  |  | 3.13 | 1.10 | 6.55 |  |  |  |  |
|  | ***qBNP-Chr21-1*** | E1 | CGR5808 | HAU0423 | 3.21 | -0.68 | 6.27 |  |  |  |  |  |  |  |  |  |  |  |  |
|  |  | E1 | HAU0423 | CGR5806 |  |  |  |  | 2.10 | -0.65 | 3.98 |  |  |  |  |  |  |  |  |
|  | ***qBNP-Chr24-1*** | E3 | Gh268 | SWU13268 |  |  |  |  |  |  |  |  | 3.68 | 1.48 | 7.49 |  |  |  |  |
|  |  | E3 | PGML4657 | Gh454 | 4.40 | 1.13 | 9.86 |  |  |  |  |  |  |  |  |  |  |  |  |
|  | ***qBNP-Chr24-2*** | E2 | SWU13758 | CGR5423 | 2.46 | 0.89 | 9.61 |  |  |  |  |  |  |  |  |  |  |  |  |
|  |  | E2 | SWU13758 | CGR5423 |  |  |  |  | 4.57 | 0.87 | 8.97 |  |  |  |  |  |  |  |  |
| BW | ***qBW-Chr2-1*** | E1 | SWU11889 | SWU11887 | 2.27 | 0.10 | 4.13 |  |  |  |  |  |  |  |  |  |  |  |  |
|  |  | E3 | SWU11976 | SWU11950 | 2.29 | 0.11 | 4.34 |  |  |  |  |  |  |  |  |  |  |  |  |
|  |  | E1 | SWU11889 | SWU11887 |  |  |  |  | 2.05 | 0.10 | 3.74 |  |  |  |  |  |  |  |  |
|  |  | E2 | SWU11887 | SWU11976 |  |  |  |  | 3.81 | 0.14 | 10.34 |  |  |  |  |  |  |  |  |
|  | ***qBW-Chr2-2*** | E3 | MGHES24 | ICR11064 |  |  |  |  |  |  |  |  | 2.91 | 0.13 | 5.80 |  |  |  |  |
|  |  | E2 | MGHES24 | ICR11064 | 4.17 | 0.12 | 7.73 |  |  |  |  |  |  |  |  |  |  |  |  |
|  |  | E1 | MGHES24 | ICR11064 |  |  |  |  | 2.69 | 0.11 | 5.21 |  |  |  |  |  |  |  |  |
|  | *qBW-Chr4-1* | E1 | BNL1167 | SWU21415 |  |  |  |  |  |  |  |  | 3.33 | 0.14 | 6.92 |  |  |  |  |
|  | ***qBW-Chr4-2*** | E3 | SWU21485 | ICR01729 |  |  |  |  |  |  |  |  | 3.15 | 0.13 | 6.15 |  |  |  |  |
|  |  | E3 | SWU21485 | ICR01729 |  |  |  |  | 3.85 | 0.12 | 7.31 |  |  |  |  |  |  |  |  |
|  | ***qBW-Chr5-1*** | E2 | SWU20913 | Gh260 | 3.53 | 0.13 | 8.94 |  |  |  |  |  |  |  |  |  |  |  |  |
|  |  | E3 | PGML0120 | SWU20914 |  |  |  |  | 5.94 | 0.15 | 11.48 |  |  |  |  |  |  |  |  |
|  | ***qBW-Chr5-2*** | E3 | SWU20917 | NAU6240 |  |  |  |  |  |  |  |  | 3.50 | 0.16 | 9.34 |  |  |  |  |
|  |  | E3 | SWU20917 | NAU6240 | 6.81 | 0.21 | 18.19 |  |  |  |  |  |  |  |  |  |  |  |  |
|  |  | E3 | SWU20917 | NAU6240 |  |  |  |  | 6.12 | 0.17 | 16.02 |  |  |  |  |  |  |  |  |
|  |  | E2 | SWU20917 | NAU6240 |  |  |  |  | 6.28 | 0.17 | 15.73 |  |  |  |  |  |  |  |  |
|  | ***qBW-Chr5-3*** | E3 | NAU6240 | PGML1671 |  |  |  |  |  |  |  |  | 3.33 | 0.17 | 9.99 |  |  |  |  |
|  |  | E2 | NAU6240 | PGML1671 | 3.35 | 0.13 | 10.18 |  |  |  |  |  |  |  |  |  |  |  |  |
|  |  | E1 | PGML1671 | PGML1917 | 4.19 | 0.15 | 9.43 |  |  |  |  |  |  |  |  |  |  |  |  |
|  | ***qBW-Chr5-4*** | E2 | PGML1671 | PGML1917 | 4.05 | 0.12 | 8.45 |  |  |  |  |  |  |  |  |  |  |  |  |
|  |  | E1 | PGML1917 | SWU17715 |  |  |  |  | 2.07 | 0.11 | 5.21 |  |  |  |  |  |  |  |  |
|  | ***qBW-Chr5-5*** | E1 | TMB1296 | HAU1603 |  |  |  |  |  |  |  |  |  |  |  |  | 2.52 | -0.12 | 5.54 |
|  |  | E1 | NAU4034 | SWU17713 |  |  |  |  | 3.55 | 0.13 | 7.03 |  |  |  |  |  |  |  |  |
|  | ***qBW-Chr5-6*** | E2 | PGML4350 | SWU17781 | 3.25 | 0.10 | 6.02 |  |  |  |  |  |  |  |  |  |  |  |  |
|  |  | E1 | PGML4350 | SWU17781 | 3.54 | 0.14 | 8.05 |  |  |  |  |  |  |  |  |  |  |  |  |
|  |  | E1 | PGML4350 | SWU17781 |  |  |  |  | 4.03 | 0.14 | 7.95 |  |  |  |  |  |  |  |  |
|  |  | E3 | PGML4350 | SWU17781 |  |  |  |  | 2.25 | 0.09 | 4.13 |  |  |  |  |  |  |  |  |
|  | ***qBW-Chr5-7*** | E3 | CGR5025 | NBRI0694 |  |  |  |  |  |  |  |  | 2.14 | 0.11 | 4.11 |  |  |  |  |
|  |  | E1 | NBRI0694 | DPL0022 | 2.28 | 0.10 | 4.53 |  |  |  |  |  |  |  |  |  |  |  |  |
|  |  | E3 | CGR5025 | NBRI0694 |  |  |  |  |  |  |  |  |  |  |  |  | 2.31 | 0.13 | 4.91 |
|  | ***qBW-Chr5-8*** | E2 | DPL0022 | SWU17787 | 2.52 | 0.09 | 5.09 |  |  |  |  |  |  |  |  |  |  |  |  |
|  |  | E1 | DPL0022 | SWU17787 |  |  |  |  | 2.16 | 0.11 | 4.77 |  |  |  |  |  |  |  |  |
|  |  | E2 | SWU13378 | SWU17846 |  |  |  |  | 2.24 | 0.09 | 3.79 |  |  |  |  |  |  |  |  |
|  | *qBW-Chr14-1* | E2 | CGR6683 | NAU3308 | 3.87 | -0.11 | 7.14 |  |  |  |  |  |  |  |  |  |  |  |  |
|  | ***qBW-Chr14-2*** | E2 | DPL0565 | Gh120 |  |  |  |  |  |  |  |  | 5.67 | -0.20 | 11.85 |  |  |  |  |
|  |  | E2 | SWU14224 | DPL0565 | 3.75 | -0.12 | 8.29 |  |  |  |  |  |  |  |  |  |  |  |  |
|  | *qBW-Chr16-1* | E3 | ICR00010 | SWU10038 |  |  |  |  |  |  |  |  |  |  |  |  | 5.15 | 0.25 | 13.11 |
|  | *qBW-Chr17-1* | E1 | ICR03391 | SWU12838a |  |  |  |  |  |  |  |  | 3.62 | 0.15 | 7.20 |  |  |  |  |
|  | ***qBW-Chr18-1*** | E3 | SWU22290 | Gh501 | 3.65 | -0.13 | 6.98 |  |  |  |  |  |  |  |  |  |  |  |  |
|  |  | E2 | SWU22192 | DPL0864 |  |  |  |  | 4.44 | -0.12 | 8.20 |  |  |  |  |  |  |  |  |
|  | *qBW-Chr19-1* | E3 | HAU3069 | PGML4342 | 4.18 | -0.14 | 8.05 |  |  |  |  |  |  |  |  |  |  |  |  |
|  | ***qBW-Chr21-1*** | E1 | BNL3171 | CGR5808 | 2.98 | -0.12 | 5.86 |  |  |  |  |  |  |  |  |  |  |  |  |
|  |  | E2 | BNL3171 | CGR5808 | 2.34 | -0.08 | 4.02 |  |  |  |  |  |  |  |  |  |  |  |  |
|  |  | E2 | BNL3171 | CGR5808 |  |  |  |  | 3.15 | -0.10 | 5.72 |  |  |  |  |  |  |  |  |
|  | ***qBW-Chr23-1*** | E1 | SWU14807 | PGML4185 |  |  |  |  | 2.14 | 0.11 | 4.48 |  |  |  |  |  |  |  |  |
|  |  | E2 | SWU14807 | PGML4185 |  |  |  |  | 3.10 | 0.11 | 6.00 |  |  |  |  |  |  |  |  |
|  | *qBW-Chr24-1* | E3 | BNL1521 | HAU2504 |  |  |  |  | 2.68 | -0.11 | 6.40 |  |  |  |  |  |  |  |  |
|  | *qBW-Chr25-1* | E1 | SWU19430 | PGML1219 |  |  |  |  |  |  |  |  |  |  |  |  | 3.50 | 0.16 | 8.55 |
| LP | ***qLP-Chr1-1*** | E1 | BNL2827a | NAU6367 |  |  |  |  |  |  |  |  | 2.15 | -0.57 | 3.96 |  |  |  |  |
|  |  | E1 | BNL2827a | NAU6367 | 2.40 | -0.48 | 4.73 |  |  |  |  |  |  |  |  |  |  |  |  |
|  | ***qLP-Chr2-1*** | E2 | SWU12025 | SWU11889 | 2.13 | 0.62 | 7.93 |  |  |  |  |  |  |  |  |  |  |  |  |
|  |  | E3 | SWU11887 | SWU11976 | 3.00 | 0.50 | 4.75 |  |  |  |  |  |  |  |  |  |  |  |  |
|  |  | E3 | SWU11889 | SWU11887 |  |  |  |  | 3.87 | 0.64 | 6.93 |  |  |  |  |  |  |  |  |
|  | ***qLP-Chr4-1*** | E1 | NAU3868 | SWU21617 |  |  |  |  |  |  |  |  | 2.93 | 0.66 | 5.60 |  |  |  |  |
|  |  | E1 | SWU16783 | NAU3868 | 3.23 | 0.53 | 5.99 |  |  |  |  |  |  |  |  |  |  |  |  |
|  |  | E2 | NAU3868 | SWU21617 | 2.39 | 0.45 | 4.19 |  |  |  |  |  |  |  |  |  |  |  |  |
|  | ***qLP-Chr5-1*** | E1 | SWU20913 | Gh260 |  |  |  |  |  |  |  |  | 6.05 | -1.05 | 14.26 |  |  |  |  |
|  |  | E3 | SWU20913 | Gh260 |  |  |  |  |  |  |  |  | 6.36 | -0.89 | 13.67 |  |  |  |  |
|  |  | E1 | SWU20913 | Gh260 | 7.96 | -0.88 | 16.29 |  |  |  |  |  |  |  |  |  |  |  |  |
|  |  | E2 | SWU20913 | Gh260 | 9.39 | -1.03 | 21.98 |  |  |  |  |  |  |  |  |  |  |  |  |
|  |  | E3 | PGML0120 | SWU20914 | 12.83 | -1.13 | 23.26 |  |  |  |  |  |  |  |  |  |  |  |  |
|  |  | E3 | SWU20913 | Gh260 |  |  |  |  | 8.03 | -1.07 | 19.78 |  |  |  |  |  |  |  |  |
|  |  | E1 | SWU20913 | Gh260 |  |  |  |  | 11.60 | -1.11 | 23.21 |  |  |  |  |  |  |  |  |
|  |  | E2 | SWU20913 | Gh260 |  |  |  |  | 10.93 | -0.94 | 21.62 |  |  |  |  |  |  |  |  |
|  | ***qLP-Chr5-2*** | E1 | SWU20917 | NAU6240 |  |  |  |  |  |  |  |  | 6.10 | -1.09 | 15.51 |  |  |  |  |
|  |  | E2 | SWU20917 | NAU6240 |  |  |  |  |  |  |  |  | 6.26 | -1.24 | 16.93 |  |  |  |  |
|  |  | E1 | SWU20917 | NAU6240 | 8.06 | -0.93 | 18.10 |  |  |  |  |  |  |  |  |  |  |  |  |
|  |  | E2 | SWU20917 | NAU6240 | 9.03 | -1.05 | 22.75 |  |  |  |  |  |  |  |  |  |  |  |  |
|  |  | E3 | SWU20917 | NAU6240 | 14.82 | -1.41 | 37.72 |  |  |  |  |  |  |  |  |  |  |  |  |
|  |  | E3 | SWU20917 | NAU6240 |  |  |  |  |  |  |  |  |  |  |  |  | 3.10 | 0.54 | 7.59 |
|  |  | E3 | SWU20917 | NAU6240 |  |  |  |  | 10.03 | -1.24 | 26.42 |  |  |  |  |  |  |  |  |
|  | *qLP-Chr5-3* | E2 | NAU6240 | PGML1671 |  |  |  |  |  |  |  |  | 5.60 | -1.21 | 15.97 |  |  |  |  |
|  | *qLP-Chr6-1* | E2 | ICR10602 | SWU19656 |  |  |  |  | 3.68 | -0.58 | 6.46 |  |  |  |  |  |  |  |  |
|  | *qLP-Chr6-2* | E2 | HAU1371 | CGR6749 |  |  |  |  | 3.04 | 0.54 | 5.10 |  |  |  |  |  |  |  |  |
|  | ***qLP-Chr7-1*** | E1 | SWU10067 | SWU10064 | 2.72 | -0.47 | 4.53 |  |  |  |  |  |  |  |  |  |  |  |  |
|  |  | E2 | NAU1357 | SWU10067 |  |  |  |  | 2.14 | -0.46 | 5.23 |  |  |  |  |  |  |  |  |
|  | *qLP-Chr9-1* | E2 | NAU1282 | CGR6771 |  |  |  |  |  |  |  |  | 3.33 | -0.90 | 8.71 |  |  |  |  |
|  | ***qLP-Chr13-1*** | E3 | PGML0014 | CGR6732 |  |  |  |  |  |  |  |  |  |  |  |  | 2.54 | 1.46 | 9.38 |
|  |  | E2 | DPL0572 | HAU2558 |  |  |  |  |  |  |  |  |  |  |  |  | 2.53 | -0.56 | 6.73 |
|  |  | E3 | DPL0572 | HAU2558 |  |  |  |  | 2.22 | -0.51 | 4.41 |  |  |  |  |  |  |  |  |
|  | ***qLP-Chr13-2*** | E3 | Gh157 | BNL1495 |  |  |  |  |  |  |  |  | 3.57 | -0.64 | 6.75 |  |  |  |  |
|  |  | E3 | Gh157 | BNL1495 | 5.28 | -0.68 | 8.61 |  |  |  |  |  |  |  |  |  |  |  |  |
|  | ***qLP-Chr13-3*** | E3 | BNL1495 | CGR5390 |  |  |  |  |  |  |  |  | 2.91 | -0.65 | 7.12 |  |  |  |  |
|  |  | E1 | DPL0894 | SWU10800 |  |  |  |  |  |  |  |  | 2.03 | -0.69 | 5.58 |  |  |  |  |
|  |  | E3 | BNL1495 | CGR5390 | 5.38 | -0.77 | 11.10 |  |  |  |  |  |  |  |  |  |  |  |  |
|  |  | E1 | DPL0894 | SWU10800 | 2.35 | -0.66 | 9.29 |  |  |  |  |  |  |  |  |  |  |  |  |
|  |  | E3 | BNL1495 | CGR5390 |  |  |  |  | 4.10 | -0.73 | 9.13 |  |  |  |  |  |  |  |  |
|  | *qLP-Chr13-4* | E1 | DPL0894 | SWU10800 |  |  |  |  | 4.01 | -0.81 | 12.42 |  |  |  |  |  |  |  |  |
|  | *qLP-Chr18-1* | E3 | DC40150 | ICR02849 |  |  |  |  |  |  |  |  |  |  |  |  | 3.21 | -1.52 | 16.20 |
|  | ***qLP-Chr19-1*** | E2 | NAU3437 | NAU2894 |  |  |  |  |  |  |  |  | 4.01 | 1.02 | 11.36 |  |  |  |  |
|  |  | E1 | NAU3437 | NAU2894 |  |  |  |  |  |  |  |  | 3.18 | 0.69 | 6.15 |  |  |  |  |
|  |  | E2 | NAU3437 | NAU2894 | 3.57 | 0.56 | 6.47 |  |  |  |  |  |  |  |  |  |  |  |  |
|  |  | E1 | HAU3069 | PGML4342 | 4.04 | 0.57 | 6.79 |  |  |  |  |  |  |  |  |  |  |  |  |
|  | ***qLP-Chr22-1*** | E3 | DPL0562 | CAU0161 |  |  |  |  | 2.23 | 0.49 | 3.86 |  |  |  |  |  |  |  |  |
|  |  | E1 | DPL0562 | CAU0161 |  |  |  |  | 2.45 | 0.72 | 9.67 |  |  |  |  |  |  |  |  |
|  | *qLP-Chr26-1* | E3 | NAU2175 | SWU17336 | 4.60 | 0.66 | 8.02 |  |  |  |  |  |  |  |  |  |  |  |  |
|  | *qLP-Chr26-2* | E3 | SWU16676 | SWU16755 |  |  |  |  |  |  |  |  | 3.07 | -0.60 | 5.78 |  |  |  |  |
| XZV hybrid | | | | | | | | | | | | | | | | | | | |
| SY | ***qSY-Chr1-1*** | E2 | SWU10912 | HAU2489 | 3.78 | -5.98 | 10.19 |  |  |  |  |  |  |  |  |  |  |  |  |
|  |  | E2 | HAU2489 | DPL0790 |  |  |  |  |  |  |  |  |  |  |  |  | 2.15 | 2.08 | 4.70 |
|  | *qSY-Chr9-1* | E2 | Gh111 | Gh27 | 3.75 | 4.56 | 8.05 |  |  |  |  |  |  |  |  |  |  |  |  |
|  | ***qSY-Chr10-1*** | E1 | CAU0234 | SWU13030 | 2.60 | -2.99 | 5.61 |  |  |  |  |  |  |  |  |  |  |  |  |
|  |  | E1 | SWU13030 | NAU4967 |  |  |  |  | 2.53 | -2.53 | 4.98 |  |  |  |  |  |  |  |  |
|  |  | E2 | SWU13030 | NAU4967 |  |  |  |  | 2.08 | -3.31 | 4.24 |  |  |  |  |  |  |  |  |
|  | ***qSY-Chr16-1*** | E3 | SWU18366 | SWU18579 | 2.65 | -9.04 | 5.50 |  |  |  |  |  |  |  |  |  |  |  |  |
|  |  | E3 | SWU18366 | SWU18579 |  |  |  |  | 2.54 | -8.70 | 5.66 |  |  |  |  |  |  |  |  |
|  | *qSY-Chr23-1* | E3 | SHIN1076 | BNL3482 |  |  |  |  |  |  |  |  | 3.43 | -5.01 | 12.55 |  |  |  |  |
|  | ***qSY-Chr23-2*** | E3 | BNL3482 | HAU0244 |  |  |  |  |  |  |  |  | 3.88 | -5.29 | 13.17 |  |  |  |  |
|  |  | E2 | BNL3482 | HAU0244 |  |  |  |  | 2.32 | -4.27 | 6.52 |  |  |  |  |  |  |  |  |
|  | *qSY-Chr23-3* | E1 | SWU0506 | SHIN0272 |  |  |  |  | 3.26 | -3.60 | 8.04 |  |  |  |  |  |  |  |  |
|  | ***qSY-Chr23-4*** | E1 | MUSB994 | NAU2238 | 2.13 | -3.00 | 4.92 |  |  |  |  |  |  |  |  |  |  |  |  |
|  |  | E2 | MUSB994 | NAU2238 | 4.15 | -5.60 | 13.00 |  |  |  |  |  |  |  |  |  |  |  |  |
|  |  | E1 | NAU2238 | NAU3588 |  |  |  |  | 4.67 | -3.68 | 10.65 |  |  |  |  |  |  |  |  |
|  |  | E2 | NAU2238 | NAU3588 | 4.54 | -5.05 | 10.64 |  |  |  |  |  |  |  |  |  |  |  |  |
|  |  | E1 | NAU3588 | NAU5373a |  |  |  |  |  |  |  |  | 3.33 | -2.79 | 6.95 |  |  |  |  |
|  | ***qSY-Chr23-5*** | E2 | NAU5373b | HAU2648 | 2.62 | -4.05 | 5.79 |  |  |  |  |  |  |  |  |  |  |  |  |
|  |  | E1 | NAU5373b | HAU2648 |  |  |  |  | 2.94 | -3.59 | 10.12 |  |  |  |  |  |  |  |  |
|  | *qSY-Chr24-1* | E2 | Gh54 | Gh454 |  |  |  |  |  |  |  |  |  |  |  |  | 3.34 | 3.46 | 10.92 |
|  | *qSY-Chr24-2* | E2 | HAU3076 | SWU13121 |  |  |  |  |  |  |  |  |  |  |  |  | 3.01 | -2.77 | 6.76 |
|  | ***qSY-Chr26-1*** | E3 | NAU3109 | CGR6772 |  |  |  |  |  |  |  |  |  |  |  |  | 2.90 | 7.36 | 11.66 |
|  |  | E3 | NAU3109 | CGR6772 | 2.60 | -11.74 | 8.66 |  |  |  |  |  |  |  |  |  |  |  |  |
|  |  | E3 | NAU3109 | CGR6772 |  |  |  |  | 2.01 | -9.74 | 7.02 |  |  |  |  |  |  |  |  |
|  | ***qSY-Chr26-2*** | E3 | SWU16777 | SWU16780 |  |  |  |  |  |  |  |  |  |  |  |  | 3.13 | -5.65 | 6.70 |
|  |  | E3 | SWU16777 | SWU16780 |  |  |  |  | 3.68 | 10.55 | 7.84 |  |  |  |  |  |  |  |  |
|  |  | E3 | SWU16777 | SWU16780 | 4.95 | 13.17 | 10.10 |  |  |  |  |  |  |  |  |  |  |  |  |
| LY | *qLY-Chr9-1* | E2 | Gh111 | Gh27 | 3.02 | 1.59 | 6.15 |  |  |  |  |  |  |  |  |  |  |  |  |
|  | ***qLY-Chr10-1*** | E1 | CAU0234 | SWU13030 | 3.91 | -1.40 | 8.12 |  |  |  |  |  |  |  |  |  |  |  |  |
|  |  | E2 | SWU13030 | NAU4967 |  |  |  |  | 2.92 | -1.52 | 5.62 |  |  |  |  |  |  |  |  |
|  | ***qLY-Chr15-1*** | E1 | NAU3736 | SWU11691 |  |  |  |  | 3.35 | 1.21 | 6.54 |  |  |  |  |  |  |  |  |
|  |  | E1 | NAU3736 | SWU11691 | 2.22 | 1.07 | 4.89 |  |  |  |  |  |  |  |  |  |  |  |  |
|  | ***qLY-Chr16-1*** | E3 | SWU18366 | SWU18579 | 2.70 | -3.78 | 5.62 |  |  |  |  |  |  |  |  |  |  |  |  |
|  |  | E3 | SWU18366 | SWU18579 |  |  |  |  | 2.18 | -3.17 | 4.80 |  |  |  |  |  |  |  |  |
|  | *qLY-Chr23-1* | E3 | BNL3482 | HAU0244 |  |  |  |  |  |  |  |  | 4.00 | -2.26 | 12.99 |  |  |  |  |
|  | *qLY-Chr23-2* | E1 | DC40286 | PGML1434 |  |  |  |  |  |  |  |  | 4.23 | -1.23 | 8.76 |  |  |  |  |
|  | ***qLY-Chr23-3*** | E1 | NAU2238 | NAU3588 |  |  |  |  | 7.00 | -1.75 | 13.49 |  |  |  |  |  |  |  |  |
|  |  | E2 | NAU2238 | NAU3588 |  |  |  |  | 5.00 | -2.16 | 11.40 |  |  |  |  |  |  |  |  |
|  |  | E2 | NAU2238 | NAU3588 | 5.61 | -2.30 | 13.05 |  |  |  |  |  |  |  |  |  |  |  |  |
|  |  | E1 | NAU3588 | NAU5373a |  |  |  |  |  |  |  |  | 4.39 | -1.23 | 8.94 |  |  |  |  |
|  | ***qLY-Chr23-4*** | E2 | NAU5373b | HAU2648 |  |  |  |  | 3.68 | -1.81 | 7.65 |  |  |  |  |  |  |  |  |
|  |  | E1 | NAU5373b | HAU2648 |  |  |  |  | 4.30 | -2.16 | 20.79 |  |  |  |  |  |  |  |  |
|  | *qLY-Chr25-1* | E2 | SWU19413 | SWU19431 |  |  |  |  |  |  |  |  | 3.28 | 1.01 | 7.06 |  |  |  |  |
|  | ***qLY-Chr26-1*** | E3 | SWU16777 | SWU16780 |  |  |  |  |  |  |  |  |  |  |  |  | 3.18 | -2.44 | 6.75 |
|  |  | E3 | SWU16777 | SWU16780 |  |  |  |  | 2.65 | 3.67 | 5.59 |  |  |  |  |  |  |  |  |
|  |  | E3 | SWU16777 | SWU16780 | 4.68 | 5.31 | 9.60 |  |  |  |  |  |  |  |  |  |  |  |  |
| BNP | ***qBNP-Chr2-1*** | E2 | SWU11976 | SWU12001 |  |  |  |  |  |  |  |  |  |  |  |  | 2.32 | -0.66 | 6.08 |
|  |  | E2 | SWU11976 | SWU12001 |  |  |  |  |  |  |  |  | 2.98 | -0.68 | 7.53 |  |  |  |  |
|  | *qBNP-Chr10-1* | E3 | NAU2139 | TMB1152 |  |  |  |  |  |  |  |  | 3.71 | -1.00 | 16.51 |  |  |  |  |
|  | *qBNP-Chr16-1* | E1 | SWU18366 | SWU18579 | 3.40 | -0.90 | 8.25 |  |  |  |  |  |  |  |  |  |  |  |  |
|  | ***qBNP-Chr23-2*** | E1 | MUSB994 | NAU2238 |  |  |  |  |  |  |  |  | 3.36 | -0.68 | 10.60 |  |  |  |  |
|  |  | E1 | MUSB994 | NAU2238 | 5.09 | -1.21 | 14.90 |  |  |  |  |  |  |  |  |  |  |  |  |
|  | ***qBNP-Chr23-3*** | E1 | NAU2238 | NAU3588 |  |  |  |  | 4.30 | -0.83 | 10.05 |  |  |  |  |  |  |  |  |
|  |  | E2 | NAU3588 | NAU5373a |  |  |  |  | 2.81 | -1.19 | 5.94 |  |  |  |  |  |  |  |  |
|  |  | E1 | NAU3588 | NAU5373a |  |  |  |  |  |  |  |  | 4.52 | -0.64 | 9.64 |  |  |  |  |
|  |  | E2 | NAU3588 | NAU5373a | 2.20 | -1.03 | 4.78 |  |  |  |  |  |  |  |  |  |  |  |  |
|  |  | E1 | NAU3588 | NAU5373a | 8.02 | -1.21 | 14.82 |  |  |  |  |  |  |  |  |  |  |  |  |
|  | ***qBNP-Chr26-1*** | E1 | DPL0057 | NAU3109 |  |  |  |  |  |  |  |  | 2.78 | -0.49 | 5.98 |  |  |  |  |
|  |  | E3 | NAU3109 | CGR6772 | 2.30 | -2.35 | 8.84 |  |  |  |  |  |  |  |  |  |  |  |  |
|  | ***qBNP-Chr26-2*** | E3 | SWU16777 | SWU16780 |  |  |  |  |  |  |  |  |  |  |  |  | 2.88 | -1.03 | 6.30 |
|  |  | E3 | SWU16777 | SWU16780 |  |  |  |  | 3.81 | 1.94 | 8.30 |  |  |  |  |  |  |  |  |
|  |  | E3 | SWU16777 | SWU16780 | 5.18 | 2.74 | 11.00 |  |  |  |  |  |  |  |  |  |  |  |  |
| BW | *qBW-Chr1-1* | E2 | PGML2498 | SWU14490 | 5.51 | -0.19 | 19.04 |  |  |  |  |  |  |  |  |  |  |  |  |
|  | ***qBW-Chr1-2*** | E1 | SWU14514 | Gh120 |  |  |  |  | 2.52 | -0.09 | 4.61 |  |  |  |  |  |  |  |  |
|  |  | E2 | SWU14514 | Gh120 |  |  |  |  |  |  |  |  | 2.72 | -0.06 | 5.73 |  |  |  |  |
|  |  | E2 | SWU14514 | Gh120 | 6.00 | -0.16 | 13.08 |  |  |  |  |  |  |  |  |  |  |  |  |
|  | *qBW-Chr6-1* | E3 | DPL0590 | NAU2971 | 3.08 | -0.16 | 7.12 |  |  |  |  |  |  |  |  |  |  |  |  |
|  | ***qBW-Chr14-1*** | E2 | TMB0071 | HAU1000 |  |  |  |  | 7.02 | -0.18 | 14.23 |  |  |  |  |  |  |  |  |
|  |  | E3 | TMB0071 | HAU1000 |  |  |  |  |  |  |  |  | 3.07 | -0.09 | 6.40 |  |  |  |  |
|  |  | E1 | TMB0071 | HAU1000 | 3.56 | -0.13 | 7.11 |  |  |  |  |  |  |  |  |  |  |  |  |
|  |  | E3 | TMB0071 | HAU1000 |  |  |  |  | 2.85 | -0.13 | 5.78 |  |  |  |  |  |  |  |  |
|  |  | E3 | TMB0071 | HAU1000 | 2.26 | -0.13 | 4.82 |  |  |  |  |  |  |  |  |  |  |  |  |
|  |  | E1 | TMB0071 | HAU1000 |  |  |  |  | 5.42 | -0.14 | 10.59 |  |  |  |  |  |  |  |  |
|  |  | E2 | HAU1000 | TMB1931 | 3.71 | -0.12 | 7.73 |  |  |  |  |  |  |  |  |  |  |  |  |
|  |  | E1 | HAU1000 | TMB1931 |  |  |  |  |  |  |  |  | 3.51 | -0.08 | 7.26 |  |  |  |  |
|  | *qBW-Chr19-1* | E3 | HAU3069 | SWU17789 |  |  |  |  | 4.97 | 0.27 | 13.68 |  |  |  |  |  |  |  |  |
|  | ***qBW-Chr20-1*** | E1 | SWU20700 | CER0167 | 2.41 | -0.11 | 4.67 |  |  |  |  |  |  |  |  |  |  |  |  |
|  |  | E3 | SWU20700 | CER0167 |  |  |  |  |  |  |  |  | 2.67 | -0.08 | 5.53 |  |  |  |  |
|  | ***qBW-Chr23-1*** | E1 | MUSB994 | NAU2238 |  |  |  |  | 4.02 | -0.12 | 8.05 |  |  |  |  |  |  |  |  |
|  |  | E2 | NAU2238 | NAU3588 | 3.16 | -0.11 | 6.50 |  |  |  |  |  |  |  |  |  |  |  |  |
|  | ***qBW-Chr23-2*** | E1 | NAU5373b | HAU2648 | 2.64 | -0.11 | 5.37 |  |  |  |  |  |  |  |  |  |  |  |  |
|  |  | E1 | NAU5373b | HAU2648 |  |  |  |  | 3.04 | -0.13 | 9.64 |  |  |  |  |  |  |  |  |
|  | *qBW-Chr26-1* | E1 | HAU1571 | CGR6477 |  |  |  |  |  |  |  |  | 3.15 | -0.09 | 9.44 |  |  |  |  |
| LP | ***qLP-Chr4-1*** | E3 | SWU16783 | SWU18876 |  |  |  |  |  |  |  |  | 2.20 | -0.33 | 10.47 |  |  |  |  |
|  |  | E1 | NAU2701 | DPL0573 |  |  |  |  |  |  |  |  | 3.08 | -0.38 | 5.93 |  |  |  |  |
|  | ***qLP-Chr6-1*** | E1 | BNL3650 | TMB2940 | 2.59 | -0.61 | 6.34 |  |  |  |  |  |  |  |  |  |  |  |  |
|  |  | E1 | TMB2940 | SWU19606 |  |  |  |  | 2.74 | -0.49 | 5.16 |  |  |  |  |  |  |  |  |
|  | ***qLP-Chr6-2*** | E1 | CGR5801 | CIR291 |  |  |  |  |  |  |  |  | 2.17 | -0.32 | 4.30 |  |  |  |  |
|  |  | E1 | CIR291 | CGR5883 | 3.19 | -0.65 | 7.12 |  |  |  |  |  |  |  |  |  |  |  |  |
|  | ***qLP-Chr6-3*** | E1 | HAU2768 | HAU0483 |  |  |  |  |  |  |  |  | 3.64 | -0.41 | 7.32 |  |  |  |  |
|  |  | E1 | HAU2768 | HAU0483 |  |  |  |  | 2.48 | -0.53 | 5.86 |  |  |  |  |  |  |  |  |
|  | ***qLP-Chr13-1*** | E3 | SWU13032 | HAU2850 |  |  |  |  |  |  |  |  | 3.03 | -0.31 | 8.95 |  |  |  |  |
|  |  | E1 | HAU2850 | HAU1908 | 2.57 | -0.53 | 4.83 |  |  |  |  |  |  |  |  |  |  |  |  |
|  |  | E3 | HAU1908 | NAU3398 | 2.79 | -1.01 | 6.22 |  |  |  |  |  |  |  |  |  |  |  |  |
|  |  | E3 | NAU3398 | CGR5331 |  |  |  |  | 2.12 | -0.43 | 4.24 |  |  |  |  |  |  |  |  |
|  | *qLP-Chr14-1* | E3 | ICR00401 | ICR03105 |  |  |  |  |  |  |  |  |  |  |  |  | 3.60 | 0.40 | 12.07 |
|  | *qLP-Chr16-1* | E1 | HAU1129 | C2_0011B |  |  |  |  |  |  |  |  |  |  |  |  | 3.07 | -0.35 | 6.93 |
|  | *qLP-Chr19-1* | E3 | SWU17897 | TMB0107 |  |  |  |  |  |  |  |  |  |  |  |  | 3.31 | -0.27 | 6.91 |
|  | ***qLP-Chr20-1*** | E2 | HAU1314 | SWU20035 |  |  |  |  |  |  |  |  |  |  |  |  | 2.22 | -0.29 | 5.64 |
|  |  | E1 | SWU20035 | SWU20012 |  |  |  |  |  |  |  |  | 2.60 | 0.34 | 4.97 |  |  |  |  |
|  |  | E2 | SWU20027 | Gh187 | 2.41 | 0.58 | 5.36 |  |  |  |  |  |  |  |  |  |  |  |  |
|  | ***qLP-Chr22-1*** | E3 | NAU2450 | PGML1942 |  |  |  |  |  |  |  |  | 2.75 | -0.27 | 6.78 |  |  |  |  |
|  |  | E3 | NAU2450 | PGML1942 |  |  |  |  | 2.09 | -0.54 | 6.87 |  |  |  |  |  |  |  |  |
|  |  | E2 | NAU2450 | PGML1942 |  |  |  |  |  |  |  |  | 2.29 | -0.38 | 7.84 |  |  |  |  |
|  | ***qLP-Chr23-1*** | E1 | MUSB994 | NAU2238 |  |  |  |  |  |  |  |  | 5.89 | -0.64 | 17.54 |  |  |  |  |
|  |  | E2 | MUSB994 | NAU2238 |  |  |  |  |  |  |  |  | 2.77 | -0.43 | 9.59 |  |  |  |  |
|  | ***qLP-Chr23-2*** | E2 | NAU2238 | NAU3588 |  |  |  |  | 3.43 | -0.68 | 6.88 |  |  |  |  |  |  |  |  |
|  |  | E2 | NAU2238 | NAU3588 | 4.29 | -0.77 | 9.35 |  |  |  |  |  |  |  |  |  |  |  |  |
|  |  | E1 | NAU2238 | NAU3588 |  |  |  |  | 5.17 | -0.73 | 11.11 |  |  |  |  |  |  |  |  |
|  |  | E2 | NAU2238 | NAU3588 |  |  |  |  |  |  |  |  | 3.65 | -0.42 | 9.34 |  |  |  |  |
|  |  | E1 | NAU2238 | NAU3588 | 6.63 | -0.95 | 15.02 |  |  |  |  |  |  |  |  |  |  |  |  |
|  |  | E1 | NAU2238 | NAU3588 |  |  |  |  |  |  |  |  | 5.67 | -0.56 | 13.39 |  |  |  |  |
|  | ***qLP-Chr23-3*** | E1 | NAU5373b | HAU2648 | 6.09 | -0.94 | 12.46 |  |  |  |  |  |  |  |  |  |  |  |  |
|  |  | E2 | NAU5373b | HAU2648 |  |  |  |  |  |  |  |  | 1.70 | -0.33 | 3.99 |  |  |  |  |
|  |  | E1 | NAU5373b | HAU2648 |  |  |  |  | 4.74 | -0.86 | 15.22 |  |  |  |  |  |  |  |  |
|  |  | E2 | NAU5373b | HAU2648 | 2.80 | -0.85 | 11.67 |  |  |  |  |  |  |  |  |  |  |  |  |
|  |  | E2 | NAU5373b | HAU2648 |  |  |  |  | 2.08 | -0.83 | 10.95 |  |  |  |  |  |  |  |  |
|  | *qLP-Chr24-1* | E2 | SWU13121 | CGR6079 |  |  |  |  | 3.32 | -0.67 | 6.65 |  |  |  |  |  |  |  |  |
|  | ***qLP-Chr26-1*** | E1 | CGR6477 | PGML2562 |  |  |  |  | 3.58 | 0.71 | 10.63 |  |  |  |  |  |  |  |  |
|  |  | E3 | CGR6477 | PGML2562 |  |  |  |  | 3.14 | 0.70 | 11.59 |  |  |  |  |  |  |  |  |
|  |  | E1 | CGR6477 | PGML2562 | 3.51 | 0.85 | 12.36 |  |  |  |  |  |  |  |  |  |  |  |  |

Bold figures indicate the QTL was detected in more than two environments or populations simultaneously

Env., Environment, E1: Handan; E2: Cangzhou; E3: Xiangyang

Effect, the genetic expectation of a QTL effect obtained is the additive effect (A) when estimated from the RILs and RIL′s, the additive and dominance effects (A+D) from the BCF_1_ mean values, and the dominance effect (D) from the MPH values

Var%, Phenotypic variation explained by a single QTL
