# Supplementary material for: Partial Dominance, Overdominance, Epistasis and QTL by Environment Interactions Contribute to Heterosis in Two Upland Cotton Hybrids
Source: G3 (Bethesda). 2015 Dec 29;6(3):499–507. doi: 10.1534/g3.115.025809 (PMC4777113; doi:10.1534/g3.115.025809)
Supplement: Supporting Information [file supp_g3.115.025809_TableS2.doc]

Table S2 Main effects and environmental interactions detected for yield and yield components in RIL and RILV populations by inclusive composite interval mapping

| Trait | Chr. | Position | Flanking markers | | LOD | V(A) | V(AE) | A | AE1 | AE2 | AE3 |
| --- | --- | --- | --- | --- | --- | --- | --- | --- | --- | --- | --- |
| RIL population | | | | | | | | | | | |
| SY | 1 | 48 | SWU10986 | NAU2218 | 6.39 | 4.22 | 0.27 | -2.85 | 1.01 | -0.62 | -0.38 |
|  | 2 | 40 | SWU11889 | SWU11887 | 7.19 | 4.41 | 2.68 | 2.90 | -0.93 | -2.19 | 3.11 |
|  | 3 | 83 | SWU12819 | SWU12765 | 2.75 | 1.81 | 0.18 | 1.85 | -0.53 | -0.28 | 0.80 |
|  | 18 | 118 | SWU22192 | DPL0864 | 2.54 | 1.64 | 0.01 | -1.76 | -0.03 | -0.11 | 0.14 |
|  | 19 | 116 | PGML4342 | SWU14431b | 3.12 | 1.80 | 0.06 | -1.85 | 0.04 | -0.42 | 0.37 |
|  | 20 | 114 | SWU1259 | SWU20033 | 3.75 | 1.98 | 0.20 | -1.94 | -0.17 | -0.66 | 0.83 |
|  | 21 | 157 | SWU15915 | SWU0189 | 4.54 | 2.95 | 1.19 | -2.39 | 1.17 | 0.98 | -2.15 |
|  | 21 | 177 | CGR5808 | HAU0423 | 3.74 | 0.74 | 1.34 | -1.19 | -0.96 | -1.29 | 2.25 |
|  | 24 | 22 | SWU13150 | PGML4657 | 2.65 | 0.42 | 1.16 | -0.90 | -1.49 | -0.55 | 2.04 |
|  | 26 | 28 | NAU2175 | SWU17336 | 5.85 | 4.11 | 1.17 | 2.80 | -1.40 | -0.67 | 2.07 |
|  | 26 | 204 | PGML1289 | SWU18919 | 5.10 | 3.40 | 0.02 | 2.56 | 0.30 | -0.18 | -0.12 |
|  | 27 | 0 | SWU10994 | HAU1001 | 3.45 | 2.24 | 0.00 | -2.07 | -0.05 | -0.08 | 0.13 |
|  | 27 | 56 | ICR11883 | CGR6356 | 4.68 | 0.35 | 2.68 | 0.82 | 1.67 | 1.53 | -3.21 |
| LY | 1 | 24 | SWU10930 | ICR03295 | 2.67 | 1.06 | 1.47 | -0.58 | 0.46 | 0.51 | -0.97 |
|  | 1 | 49 | SWU10986 | NAU2218 | 5.18 | 2.69 | 0.29 | -0.93 | 0.20 | -0.43 | 0.23 |
|  | 2 | 41 | SWU11889 | SWU11887 | 10.72 | 6.57 | 3.30 | 1.45 | -0.40 | -1.01 | 1.41 |
|  | 3 | 114 | SWU12765 | NAU3839 | 3.09 | 1.91 | 0.25 | 0.78 | -0.06 | -0.31 | 0.37 |
|  | 17 | 83 | SWU14627 | CGR5871 | 2.63 | 1.27 | 0.59 | 0.64 | 0.14 | -0.59 | 0.45 |
|  | 18 | 118 | SWU22192 | DPL0864 | 2.62 | 1.59 | 0.01 | -0.71 | -0.06 | 0.01 | 0.04 |
|  | 19 | 119 | PGML4342 | SWU14431b | 4.02 | 2.43 | 0.00 | -0.88 | 0.00 | -0.05 | 0.05 |
|  | 20 | 114 | SWU1259 | SWU20033 | 2.82 | 1.33 | 0.19 | -0.65 | -0.04 | -0.28 | 0.32 |
|  | 21 | 156 | SWU14431a | SWU15915 | 3.76 | 2.17 | 1.43 | -0.84 | 0.54 | 0.42 | -0.97 |
|  | 21 | 179 | CGR5808 | HAU0423 | 2.76 | 0.40 | 1.03 | -0.36 | -0.28 | -0.52 | 0.80 |
|  | 24 | 21 | SWU13150 | PGML4657 | 3.11 | 0.26 | 1.62 | -0.29 | -0.72 | -0.27 | 0.99 |
|  | 26 | 28 | NAU2175 | SWU17336 | 6.56 | 4.35 | 1.31 | 1.18 | -0.60 | -0.30 | 0.90 |
|  | 26 | 203 | PGML1289 | SWU18919 | 4.01 | 2.34 | 0.03 | 0.87 | 0.11 | 0.03 | -0.14 |
|  | 27 | 0 | SWU10994 | HAU1001 | 2.71 | 1.73 | 0.01 | -0.74 | -0.03 | 0.08 | -0.05 |
|  | 27 | 56 | ICR11883 | CGR6356 | 4.96 | 0.36 | 2.60 | 0.34 | 0.63 | 0.66 | -1.29 |
| BNP | 1 | 48 | SWU10986 | NAU2218 | 6.56 | 4.67 | 0.15 | -0.67 | 0.16 | -0.12 | -0.04 |
|  | 2 | 41 | SWU11889 | SWU11887 | 3.56 | 1.19 | 1.72 | 0.34 | 0.08 | -0.53 | 0.45 |
|  | 3 | 119 | SWU12765 | NAU3839 | 2.51 | 1.73 | 0.17 | 0.41 | 0.17 | -0.13 | -0.04 |
|  | 5 | 115 | TMB1296 | HAU1603 | 2.56 | 1.22 | 1.08 | -0.34 | 0.03 | 0.38 | -0.41 |
|  | 5 | 136 | SWU17787 | SWU13378 | 2.87 | 2.04 | 0.27 | -0.44 | -0.13 | 0.23 | -0.10 |
|  | 11 | 199 | NAU1014 | ICR10344 | 3.19 | 2.03 | 0.58 | -0.44 | 0.19 | 0.14 | -0.33 |
|  | 12 | 59 | Gh631 | HAU1321 | 3.29 | 1.52 | 0.57 | -0.38 | 0.13 | -0.33 | 0.19 |
|  | 19 | 116 | PGML4342 | SWU14431b | 2.93 | 2.02 | 0.00 | -0.44 | -0.02 | -0.01 | 0.03 |
|  | 20 | 138 | DPL0319 | HAU1378 | 2.90 | 1.80 | 0.32 | -0.41 | -0.25 | 0.12 | 0.13 |
|  | 22 | 2 | SWU21646 | SWU21585 | 4.71 | 3.49 | 0.11 | -0.58 | 0.10 | 0.04 | -0.14 |
|  | 24 | 86 | SWU13745 | Gh273 | 3.98 | 1.80 | 0.86 | 0.41 | -0.35 | 0.35 | -0.01 |
|  | 25 | 55 | BNL3594 | DPL0282 | 2.60 | 1.84 | 0.01 | -0.42 | 0.04 | -0.03 | -0.02 |
|  | 27 | 0 | SWU10994 | HAU1001 | 5.15 | 3.53 | 0.51 | -0.58 | -0.31 | 0.16 | 0.15 |
| BW | 2 | 40 | SWU11889 | SWU11887 | 4.87 | 3.04 | 0.10 | 0.08 | 0.00 | 0.02 | -0.02 |
|  | 4 | 46 | SWU21415 | BNL530 | 4.53 | 2.50 | 0.59 | 0.07 | 0.00 | -0.04 | 0.04 |
|  | 5 | 14 | SWU20913 | Gh260 | 7.34 | 2.66 | 1.82 | 0.07 | 0.00 | 0.07 | -0.07 |
|  | 5 | 38 | NAU6240 | PGML1671 | 3.46 | 2.21 | 0.03 | 0.07 | 0.01 | -0.01 | 0.00 |
|  | 5 | 68 | PGML1917 | SWU17715 | 3.98 | 2.59 | 0.18 | 0.07 | 0.03 | -0.02 | -0.01 |
|  | 5 | 124 | PGML4350 | SWU17781 | 6.74 | 4.43 | 0.44 | 0.09 | 0.04 | -0.01 | -0.03 |
|  | 5 | 151 | SWU17787 | SWU13378 | 2.66 | 1.15 | 0.34 | 0.05 | -0.01 | 0.03 | -0.03 |
|  | 6 | 48 | ICR03206 | NAU896 | 2.85 | 1.42 | 0.19 | 0.05 | -0.02 | 0.03 | -0.01 |
|  | 12 | 4 | NAU943 | DPL0303 | 2.90 | 1.49 | 0.18 | -0.06 | 0.03 | -0.02 | -0.01 |
|  | 14 | 62 | SWU14224 | DPL0565 | 3.95 | 2.53 | 0.08 | -0.07 | -0.02 | 0.01 | 0.01 |
|  | 16 | 45 | DPL0048 | SWU10266 | 2.55 | 1.67 | 0.02 | -0.06 | -0.01 | 0.01 | 0.00 |
|  | 18 | 116 | NAU748 | SWU22192 | 5.47 | 2.86 | 0.40 | -0.08 | 0.02 | -0.04 | 0.02 |
|  | 21 | 170 | BNL3171 | CGR5808 | 3.89 | 2.03 | 0.31 | -0.06 | 0.01 | -0.03 | 0.02 |
|  | 22 | 32 | DPL0562 | CAU0161 | 3.00 | 1.46 | 0.99 | 0.06 | 0.06 | -0.04 | -0.02 |
|  | 23 | 9 | Gh327 | SWU14770 | 6.42 | 3.19 | 1.03 | 0.08 | 0.03 | 0.04 | -0.07 |
|  | 28 | 93 | MGHES24 | ICR11064 | 3.92 | 2.60 | 0.11 | 0.07 | 0.02 | -0.02 | 0.00 |
| LP | 1 | 103 | SWU11191 | BNL2827b | 2.84 | 1.70 | 0.19 | -0.29 | -0.02 | 0.13 | -0.11 |
|  | 1 | 126 | NAU3384 | CGR5663 | 3.55 | 2.30 | 0.00 | -0.34 | -0.01 | 0.01 | 0.01 |
|  | 2 | 41 | SWU11889 | SWU11887 | 4.82 | 2.64 | 0.75 | 0.36 | -0.04 | -0.21 | 0.25 |
|  | 4 | 91 | SWU16783 | NAU3868 | 2.98 | 1.69 | 0.33 | 0.29 | 0.18 | -0.10 | -0.07 |
|  | 5 | 14 | SWU20913 | Gh260 | 18.60 | 8.91 | 4.12 | -0.66 | -0.34 | -0.30 | 0.64 |
|  | 5 | 115 | TMB1296 | HAU1603 | 4.62 | 2.90 | 0.02 | -0.38 | 0.01 | 0.04 | -0.05 |
|  | 6 | 79 | BNL3650 | ICR10602 | 2.68 | 1.70 | 0.05 | -0.29 | -0.04 | -0.03 | 0.07 |
|  | 7 | 24 | SWU10064 | NAU3181 | 4.23 | 2.66 | 0.14 | -0.36 | -0.04 | 0.11 | -0.08 |
|  | 11 | 116 | ICR01810 | CGR6525 | 3.66 | 2.31 | 0.06 | 0.34 | 0.07 | -0.07 | 0.00 |
|  | 13 | 72 | Gh157 | BNL1495 | 5.21 | 2.76 | 0.92 | -0.37 | -0.06 | 0.29 | -0.23 |
|  | 13 | 123 | DPL0894 | SWU10800 | 2.66 | 1.00 | 0.76 | -0.22 | -0.24 | 0.23 | 0.01 |
|  | 14 | 58 | NAU3308 | HAU1057 | 3.84 | 2.26 | 0.06 | 0.34 | 0.01 | 0.06 | -0.07 |
|  | 14 | 73 | Gh120 | PGML1884 | 2.89 | 1.48 | 0.30 | 0.27 | 0.09 | 0.09 | -0.17 |
|  | 14 | 121 | PGML1568 | Gh529 | 2.58 | 1.53 | 0.09 | 0.27 | 0.05 | 0.05 | -0.09 |
|  | 15 | 0 | DC40183 | DC40175 | 2.85 | 1.75 | 0.04 | -0.29 | -0.05 | -0.01 | 0.06 |
|  | 22 | 32 | DPL0562 | CAU0161 | 5.42 | 3.45 | 0.23 | 0.43 | 0.05 | -0.15 | 0.10 |
|  | 23 | 9 | Gh327 | SWU14770 | 3.79 | 2.30 | 0.02 | -0.34 | 0.04 | -0.02 | -0.02 |
|  | 26 | 48 | BNL2495 | DPL0491 | 2.54 | 1.33 | 0.31 | 0.26 | -0.09 | -0.08 | 0.17 |
| RILV population | | | | | | | | | | | |
| SY | 10 | 19 | CAU0234 | SWU13030 | 2.70 | 0.40 | 0.33 | -1.45 | -0.53 | -1.29 | 1.82 |
|  | 15 | 0 | NAU3736 | SWU11691 | 4.19 | 1.76 | 0.07 | 3.04 | -0.31 | -0.56 | 0.87 |
|  | 23 | 91 | BNL3482 | HAU0244 | 2.87 | 0.84 | 0.31 | -2.20 | 1.25 | -1.85 | 0.60 |
|  | 23 | 275 | NAU2238 | NAU3588 | 5.42 | 0.84 | 0.51 | -2.09 | -1.05 | -1.25 | 2.30 |
|  | 26 | 52 | DC30107 | DPL0070 | 3.35 | 2.52 | 2.87 | 3.62 | -3.54 | -1.83 | 5.38 |
|  | 31 | 67 | HAU0355 | SWU16777 | 3.06 | 1.28 | 2.48 | 2.58 | -3.85 | -0.95 | 4.80 |
| LY | 15 | 0 | NAU3736 | SWU11691 | 6.07 | 2.36 | 0.07 | 1.44 | -0.23 | -0.13 | 0.36 |
|  | 16 | 100 | SWU18366 | SWU18579 | 2.50 | 1.74 | 0.91 | -1.24 | 0.90 | 0.32 | -1.22 |
|  | 19 | 50 | SWU17897 | TMB0107 | 3.24 | 1.44 | 0.14 | 1.13 | -0.30 | -0.20 | 0.50 |
|  | 23 | 151 | SWU0506 | SHIN0272 | 2.94 | 0.41 | 0.22 | -0.68 | -0.48 | 0.68 | -0.20 |
|  | 23 | 275 | NAU2238 | NAU3588 | 9.87 | 1.48 | 0.89 | -1.14 | -0.49 | -0.75 | 1.24 |
|  | 24 | 84 | CGR6079 | SWU13100 | 3.11 | 0.00 | 1.34 | 0.06 | -1.04 | -0.45 | 1.49 |
|  | 26 | 52 | DC30107 | DPL0070 | 4.16 | 3.23 | 2.58 | 1.68 | -1.23 | -0.88 | 2.11 |
| BNP | 15 | 0 | NAU3736 | SWU11691 | 4.39 | 2.52 | 0.10 | 0.76 | -0.19 | 0.18 | 0.01 |
|  | 16 | 100 | SWU18366 | SWU18579 | 3.22 | 2.31 | 0.41 | -0.73 | 0.43 | -0.25 | -0.18 |
|  | 23 | 91 | BNL3482 | HAU0244 | 2.95 | 1.23 | 0.23 | -0.55 | 0.02 | -0.30 | 0.28 |
|  | 23 | 278 | NAU2238 | NAU3588 | 6.40 | 2.07 | 0.93 | -0.69 | -0.12 | -0.49 | 0.61 |
|  | 26 | 52 | DC30107 | DPL0070 | 3.96 | 2.97 | 0.48 | 0.82 | -0.42 | 0.03 | 0.39 |
|  | 31 | 67 | HAU0355 | SWU16777 | 3.63 | 2.15 | 2.10 | 0.70 | -0.85 | 0.02 | 0.84 |
|  | 31 | 87 | SWU16735 | SWU16755 | 2.53 | 1.94 | 0.45 | -0.66 | 0.39 | 0.00 | -0.39 |
| BW | 1 | 83 | SWU14514 | Gh120 | 6.47 | 3.89 | 0.13 | -0.09 | -0.01 | -0.01 | 0.02 |
|  | 4 | 55 | SWU16783 | SWU18876 | 2.74 | 1.72 | 0.03 | 0.06 | 0.01 | -0.01 | 0.00 |
|  | 9 | 30 | PGML2830 | DC30015 | 2.55 | 1.67 | 0.02 | 0.06 | -0.01 | 0.01 | 0.00 |
|  | 14 | 91 | HAU2482 | NAU4045 | 3.14 | 1.06 | 1.88 | -0.06 | 0.08 | 0.02 | -0.10 |
|  | 19 | 18 | HAU3069 | SWU17789 | 4.54 | 3.16 | 0.15 | 0.08 | -0.02 | 0.00 | 0.02 |
|  | 23 | 1 | CGR5158 | HAU1758 | 2.56 | 1.31 | 0.18 | -0.06 | -0.02 | 0.03 | 0.00 |
|  | 23 | 270 | MUSB994 | NAU2238 | 6.40 | 2.28 | 1.10 | -0.07 | -0.05 | -0.01 | 0.06 |
|  | 24 | 83 | SWU13121 | CGR6079 | 5.30 | 2.60 | 0.46 | -0.08 | -0.04 | 0.03 | 0.02 |
|  | 26 | 27 | CGR6477 | PGML2562 | 4.35 | 1.57 | 0.81 | -0.06 | -0.02 | -0.04 | 0.06 |
|  | 29 | 12 | SHIN0830 | Gh111 | 4.64 | 3.02 | 0.01 | 0.08 | -0.01 | 0.00 | 0.00 |
|  | 31 | 115 | SWU16730 | SWU16721 | 3.44 | 1.84 | 0.53 | -0.06 | 0.05 | -0.04 | -0.01 |
|  | 32 | 1 | TMB0071 | HAU1000 | 10.66 | 7.19 | 0.01 | -0.13 | -0.01 | 0.00 | 0.01 |
|  | 38 | 16 | NAU2450 | PGML1942 | 2.99 | 2.02 | 0.04 | 0.07 | -0.01 | 0.00 | 0.01 |
| LP | 1 | 262 | SWU11632 | SWU21958 | 2.89 | 2.09 | 0.09 | -0.33 | -0.03 | -0.06 | 0.09 |
|  | 1 | 330 | NAU2697 | SWU0320 | 3.52 | 2.63 | 0.11 | -0.36 | 0.04 | -0.11 | 0.06 |
|  | 4 | 92 | NAU2701 | DPL0573 | 3.05 | 2.23 | 0.05 | -0.33 | 0.02 | -0.07 | 0.05 |
|  | 6 | 68 | CGR5801 | CIR291 | 5.05 | 3.29 | 0.15 | -0.41 | -0.12 | 0.09 | 0.03 |
|  | 13 | 33 | NAU3398 | CGR5331 | 3.57 | 1.84 | 0.54 | -0.30 | 0.01 | 0.20 | -0.21 |
|  | 15 | 0 | NAU3736 | SWU11691 | 3.57 | 2.22 | 0.18 | 0.33 | 0.13 | -0.04 | -0.09 |
|  | 19 | 21 | HAU3069 | SWU17789 | 2.90 | 1.89 | 0.05 | -0.31 | 0.01 | 0.05 | -0.06 |
|  | 23 | 75 | BNL3482 | HAU0244 | 3.02 | 1.71 | 0.26 | -0.31 | -0.01 | 0.15 | -0.14 |
|  | 23 | 274 | NAU2238 | NAU3588 | 9.61 | 4.11 | 3.44 | -0.45 | -0.24 | -0.35 | 0.58 |
|  | 24 | 85 | CGR6079 | SWU13100 | 4.00 | 2.78 | 0.37 | -0.37 | 0.02 | -0.18 | 0.15 |
|  | 26 | 34 | CGR6477 | PGML2562 | 5.93 | 3.69 | 0.33 | 0.43 | 0.16 | -0.16 | -0.01 |
|  | 31 | 0 | DPL0057 | NAU3109 | 2.97 | 2.00 | 0.04 | -0.32 | -0.06 | 0.04 | 0.02 |

Position: The scanning position in cM on the linkage group.

V(A): Phenotypic variation explained by additive and dominance effect at the current scanning position.

V(AE): Phenotypic variation explained by additive and dominance by environment effect at the current scanning position.

AE effect, the additive and dominance effect.

AE effect, the additive and dominance × environment effect.

AE1 to AE3 represent AE effects detected in Handan, Cangzhou and Xiangyang, respectively.
